# Tailored Functionalization of Natural Phenols to Improve Biological Activity

**DOI:** 10.3390/biom11091325

**Published:** 2021-09-07

**Authors:** Barbara Floris, Pierluca Galloni, Valeria Conte, Federica Sabuzi

**Affiliations:** Department of Chemical Science and Technologies, University of Rome Tor Vergata, Via della Ricerca Scientifica, snc, 00133 Roma, Italy; floris@uniroma2.it (B.F.); galloni@scienze.uniroma2.it (P.G.); valeria.conte@uniroma2.it (V.C.)

**Keywords:** carvacrol, thymol, eugenol, resveratrol, hispolon, hydroxytyrosol, lipidic phenols, phenolic acids, polyphenols, curcumin

## Abstract

Phenols are widespread in nature, being the major components of several plants and essential oils. Natural phenols’ anti-microbial, anti-bacterial, anti-oxidant, pharmacological and nutritional properties are, nowadays, well established. Hence, given their peculiar biological role, numerous studies are currently ongoing to overcome their limitations, as well as to enhance their activity. In this review, the functionalization of selected natural phenols is critically examined, mainly highlighting their improved bioactivity after the proper chemical transformations. In particular, functionalization of the most abundant naturally occurring monophenols, diphenols, lipidic phenols, phenolic acids, polyphenols and curcumin derivatives is explored.

## 1. Introduction

Natural phenols, mainly of vegetable origin, are receiving increasing attention, as insight into their biological activity increases.

In recent years, many reviews appeared about phenolic profiles of plants and/or essential oils, evidencing anti-microbial, anti-bacterial [1,2,3,4], antioxidant [5,6,7,8,9,10], as well as pharmacological [11,12,13,14,15,16,17,18] and nutritional [19,20,21] properties, together with a very informing book [22]. In view of their importance, studies were aimed at breeding plants able to increase the content of bioactive phenols [23]. The research in the field continues, and more and more plants are investigated for their phenolic content and related bioactivity [24,25,26,27,28,29,30,31,32,33,34,35,36,37,38,39,40,41]. The antioxidant activity of natural phenols has been related to their scavenger ability towards free radicals [42]. Particularly interesting is the possibility to encapsulate phenols—as well as other natural compounds—in chitosan biopolymers [43], or in β-cyclodextrin [44].

It must be noticed that the application of modern extractive techniques [45,46,47,48,49,50,51,52] makes the determination of phenolic compounds in plant matrices more accessible and complete.

New applications of natural phenols in different fields are reported in fish aquaculture [53], sport performances [54], fish gelatin and gelatin from bovine skin modification by cross-linking with natural phenolic acids [55,56]. Advanced extraction technologies allowed the use of phenolic extracts from some plants for food preservation [57,58,59,60]. Moreover, technological applications are becoming available, such as anti-bacterial films based on cellulose/phenolic species [61], antimicrobials packaging films based on nano-encapsulation of bioactive oils through emulsion polymerization [62], fire-resistant phenolic foams [63] and natural fiber-reinforced composites with lignin phenol binder [64].

Though outside the scope of the present review, it is worth signaling the use of natural phenolic compounds as building blocks to obtain functional materials [65] or as antioxidants for biodiesel [66].

With so much information collected and available, the next step was the effort to understand the structural factors responsible for bioactivity, examining the structure–activity relationship of antioxidant phenolic compounds [67,68].

From the chemical point of view, it may be interesting to look for chemical derivatization of natural phenols leading to eventually enhanced biological activity. As a matter of fact, treatment with diazomethane of phenolic extracts led to derivatives more suitable as antioxidants for lipophilic foods [69]. Considering the importance for human health, representative methods to chemically modify the natural phenols were discussed [70], as well as reviews of enzymatic modification [71] and of metabolic engineering for microbial biosynthesis of natural compounds, among which phenols, were reported [72].

In the present review, we aim to give a general picture of the situation, reporting chemically modified natural phenols and comparing their performances with those of the parent compounds. The number of isolated and bioactive natural phenols is huge and ever-increasing, so our attention is mainly focused on the most abundant ones in nature. Moreover, phenolic polymers are not discussed since they deserve a separate review, considering their growing importance. Literature published from 2000 to the beginning of 2021 is considered.

## 2. Monophenols

Monophenol functionalization is attracting the interest of a growing number of researchers, since the synthesis of new biologically active derivatives starting from natural compounds is a proficient tool to improve their properties. In fact, tailored functionalization is a valuable strategy to overcome natural phenol weaknesses such as toxicity, low water solubility, as well as to mild their strong fragrances, that often limit their application [73,74,75,76,77,78].

**Scheme 1:**
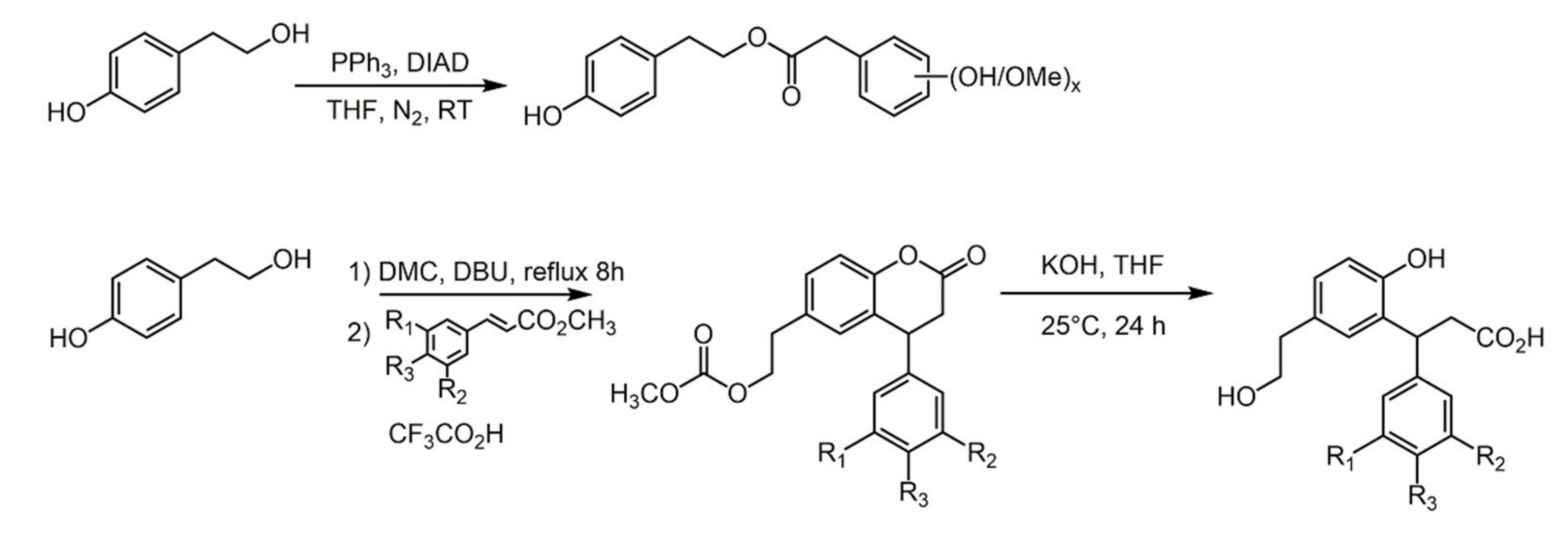
Tyrosol esterification with phenolic acids (top) [80]; tyrosol hydroarylation with cinnamic esters (bottom) [81]. Abbreviations: DIAD = diisopropyl azodicarboxylate; DMC = dimethyl carbonate; DBU = 1,8-diazabicyclo(5.4.0)undec-7-ene.

As an example, the antioxidant activity of tyrosol (2-(4-hydroxyphenyl)-ethanol), which is an abundant phenol in olive oil, responsible for oil beneficial properties [79], can be sensibly enhanced through esterification of the alcoholic hydroxyl group with different phenolic acids (Scheme 1) [80]. Analogously, hydroarylation with cinnamic esters improves the antioxidant properties of tyrosol, especially in the presence of additional hydroxyl group in the aromatic ring of the acidic moiety (Scheme 1) [81].

Nevertheless, considering their abundance in nature, we examine in detail the functionalization of carvacrol, thymol and eugenol, since they are amongst the most widespread phenols in nature, usually responsible for beneficial plant properties.

### 2.1. Carvacrol

Carvacrol (5-isopropyl-2-methylphenol) is a phenolic monoterpenoid compound, and it is a major component of oregano and thyme essential oils. Together with its isomer, thymol (2-isopropyl-5-methylphenol), it is the main active ingredient responsible for essential oils’ biological activity [82,83,84]. In fact, carvacrol’s peculiar antibacterial, anti-fungal, anti-inflammatory, anxiolytic and anticancer activities are currently well established, and the FDA (Food and Drug Administration) has approved its use as an additive in food products.

Nonetheless, the research of new carvacrol analogues is currently inspiring several research groups, with the aim to extend the potential application of the compound [85]. Carvacrol functionalization usually occurs at the -OH moiety; indeed, a wide variety of synthetic carvacryl esters can be found in the literature. Obviously, through phenol esterification, variegated functionalized products can be accessed [86], to be explored in several areas. As an example, carvacryl acetate showed significant anti-inflammatory [87], anti-nociceptive [87], anti-oxidant [88] and anti-fungal [89] effects. It can also be used in the treatment of anxiety disorders [90] and as an acaricidal agent against *Rhipicephalus microplus*, a dangerous cattle tick that is causing important economic losses in the cattle industry [91,92]. Similarly, carvacryl propionate, obtained by carvacrol esterification with propionyl chloride in the presence of triethylamine (TEA), showed higher analgesic, anti-inflammatory and anti-hyperalgesic effects compared to pure carvacrol [93]. Interestingly, esterification with the Boc-protected γ-aminobutanoic acid (GABA), which is the primary inhibitory neurotransmitter of the central nervous system, has been performed with *N*,*N*′-dicyclohexylcarbodiimide (DCC) and 4-dimethylaminopyridine (DMAP) in dichloromethane (DCM) [94,95]. The corresponding ester, obtained upon Boc removal in acid conditions, is a suitable drug for different pharmacological applications. In fact, it can modulate the transient receptor potential (TRP) channels and bind GABA receptors, thus exerting high analgesic and anti-inflammatory effects. In addition, carvacryl esters having hydroxy substituted cinnamic acids are efficient tyrosinase inhibitors [96].

However, it is worth mentioning that esterification is not always a successful strategy to obtain highly effective derivatives. In fact, the antibacterial activity of carvacrol against *S. mutans*, *S. aureus*, *B. subtilis*, *S. epidermidis* and *E. coli* was reduced upon esterification with different alkyl- or aryl-based acyl chlorides [97]. Similarly, several attempts have been performed to further improve carvacrol activity against *Mycobacterium tuberculosis* chorismate mutase enzyme: acetylation or etherification of the -OH group, or introduction of different substituents (-Cl, -Br, -NO_2_) on carvacrol aromatic rings led to unsatisfactory antitubercular activity [98].

**Scheme 2:**
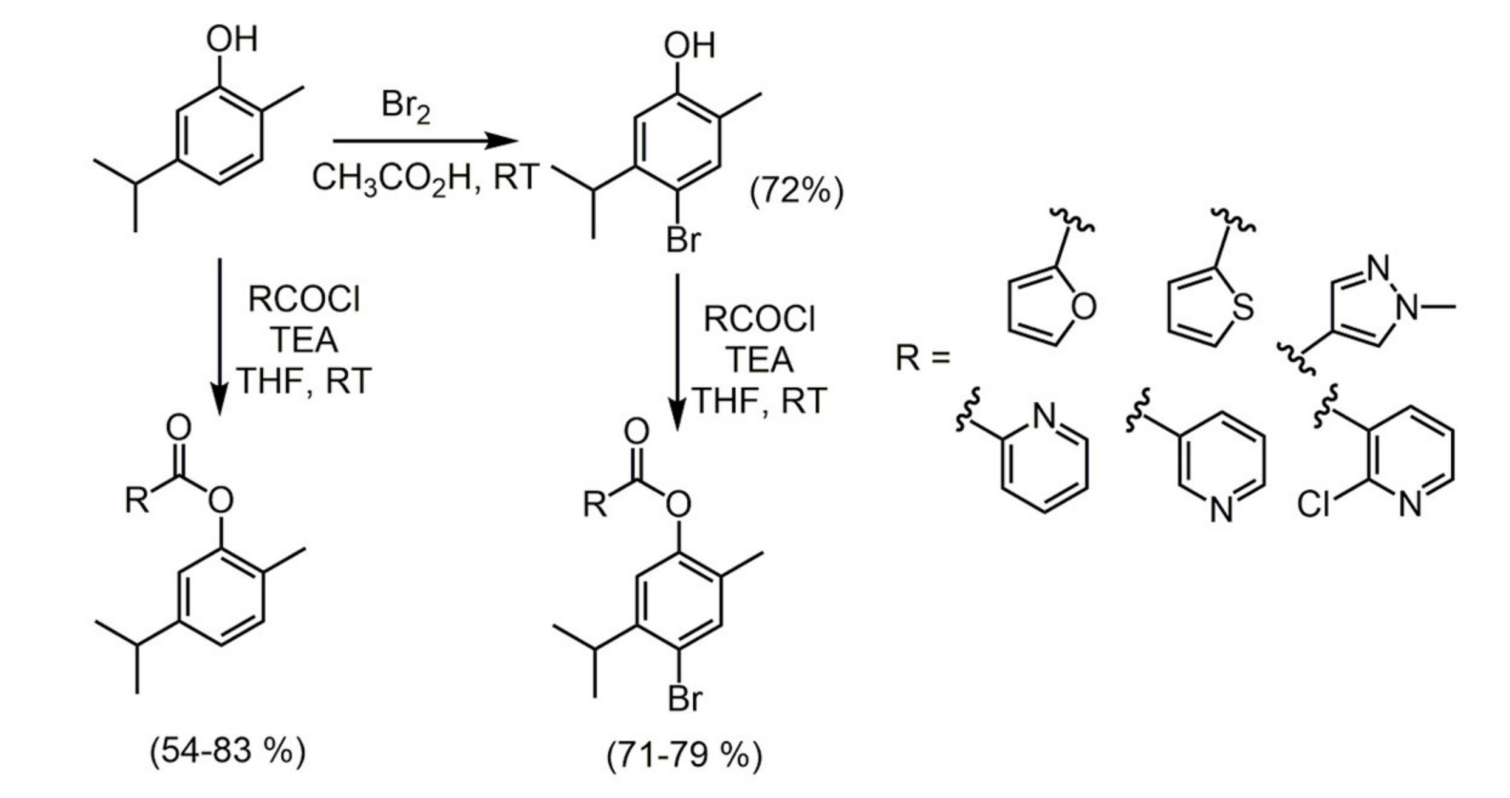
Carvacrol and 4-bromocarvacrol esterification with heterocyclic acyl halides [99].

On the contrary, a number of carvacrol and 4-bromocarvacrol esters with furan, thiophene and pyridine have been synthesized and screened as antifungal agents (Scheme 2) [99].

The different heterocyclic units sensibly influence carvacrol activity: esters with acids of furan and thiophene are more active than carvacrol against *R. solani*, while pyridine esters of 4-bromocarvacrol exhibited an enhanced antifungal activity vs. *P. oryzae*.

Carvacrol sulfenate esters, obtained by treating carvacrol with trichloromethyl hypochlorothionite (ClSCCl_3_) in the presence of TEA, are remarkable antibacterial agents, being 40 times more effective than carvacrol toward *S. epidermidis*, and 8 times more active toward *P. aeruginosa* [100]. Moreover, 4-chlorocarvacrol, obtained through the oxychlorination of carvacrol in acetic acid, with LiCl and CuCl_2_ catalyst, under O_2_ atmosphere, showed a good activity against several bacterial strains. In particular, it is much more effective than its precursor against *P. aeruginosa* [101].

**Scheme 3:**
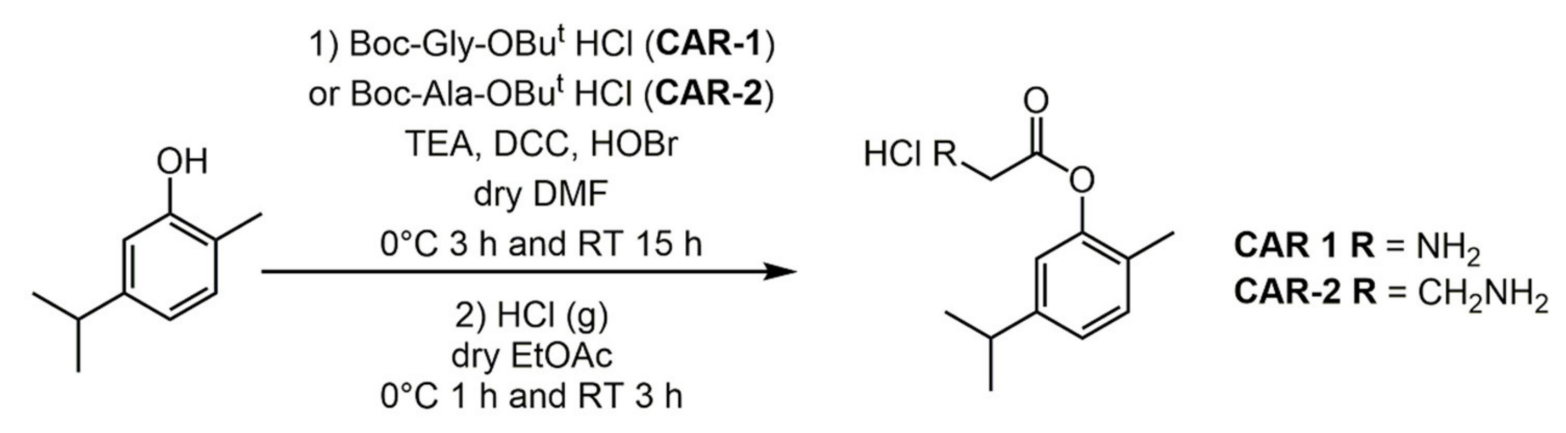
Synthesis of carvacrol amino acid ester prodrugs **CAR-1** and **CAR-2** [102].

Recently, twenty different amino acid ester prodrugs of carvacrol were synthesized, with the aim to improve carvacrol’s solubility in water while preserving its antimicrobial properties [102]. **CAR-1** is highly effective to inhibit *C. albicans* growth, while *C. tropicalis* and *C. glabrata* were successfully inhibited by **CAR-2** (Scheme 3). Importantly, **CAR-1** and **CAR-2** did not prove to be cytotoxic at the adopted concentrations.

**Scheme 4:**
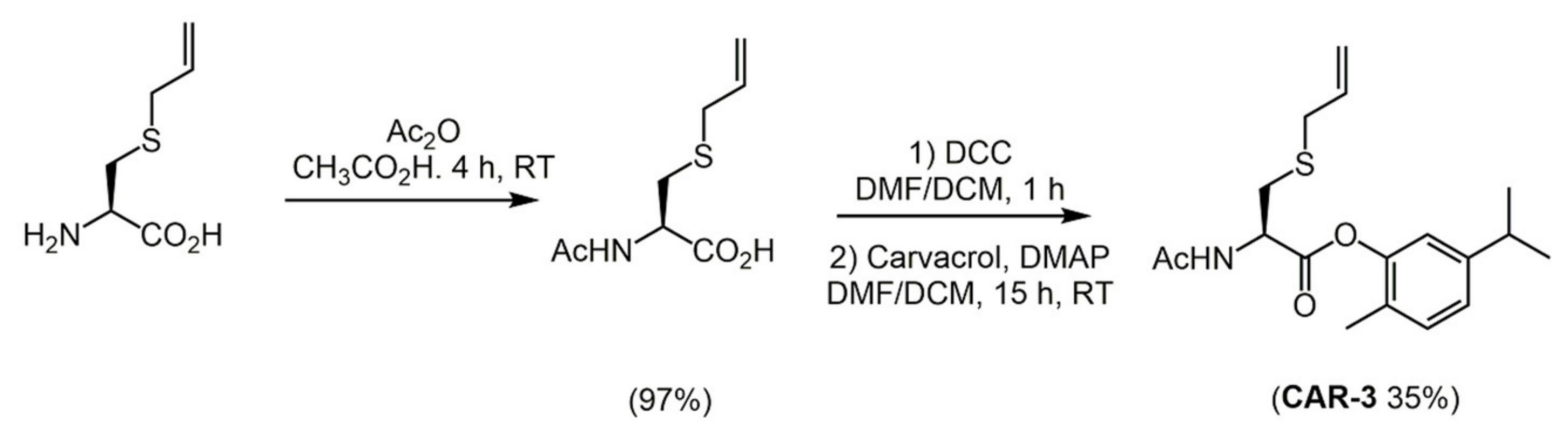
Synthesis of sulfur containing amino acid ester prodrug **CAR-3** [103]. Abbreviations: Ac_2_O = acetic anhydride.

Similarly, ten carvacrol codrugs, obtained through carvacrol esterification with sulfur-containing amino acids, have been synthesized [103]. Even though such compounds showed a reduced toxicity with respect to carvacrol, their antimicrobial activity was poorer. However, **CAR-3** (Scheme 4) is more effective than the corresponding free phenol in affecting *E. coli* mature biofilm. In fact, carvacrol conjugation with Ac-Cys(Allyl)-OH is crucial to promote the permeabilization and the destabilization of the bacterial membrane, thus ensuring reduced biofilm formation. Pharmacokinetic studies also revealed a good stability of **CAR-3** at stomach pH, in the presence of pepsin and pancreatin, suggesting that after oral administration, **CAR-3** can cross the stomach, and it can be absorbed from the intestine, releasing carvacrol after enzymatic hydrolysis.

**Scheme 5:**
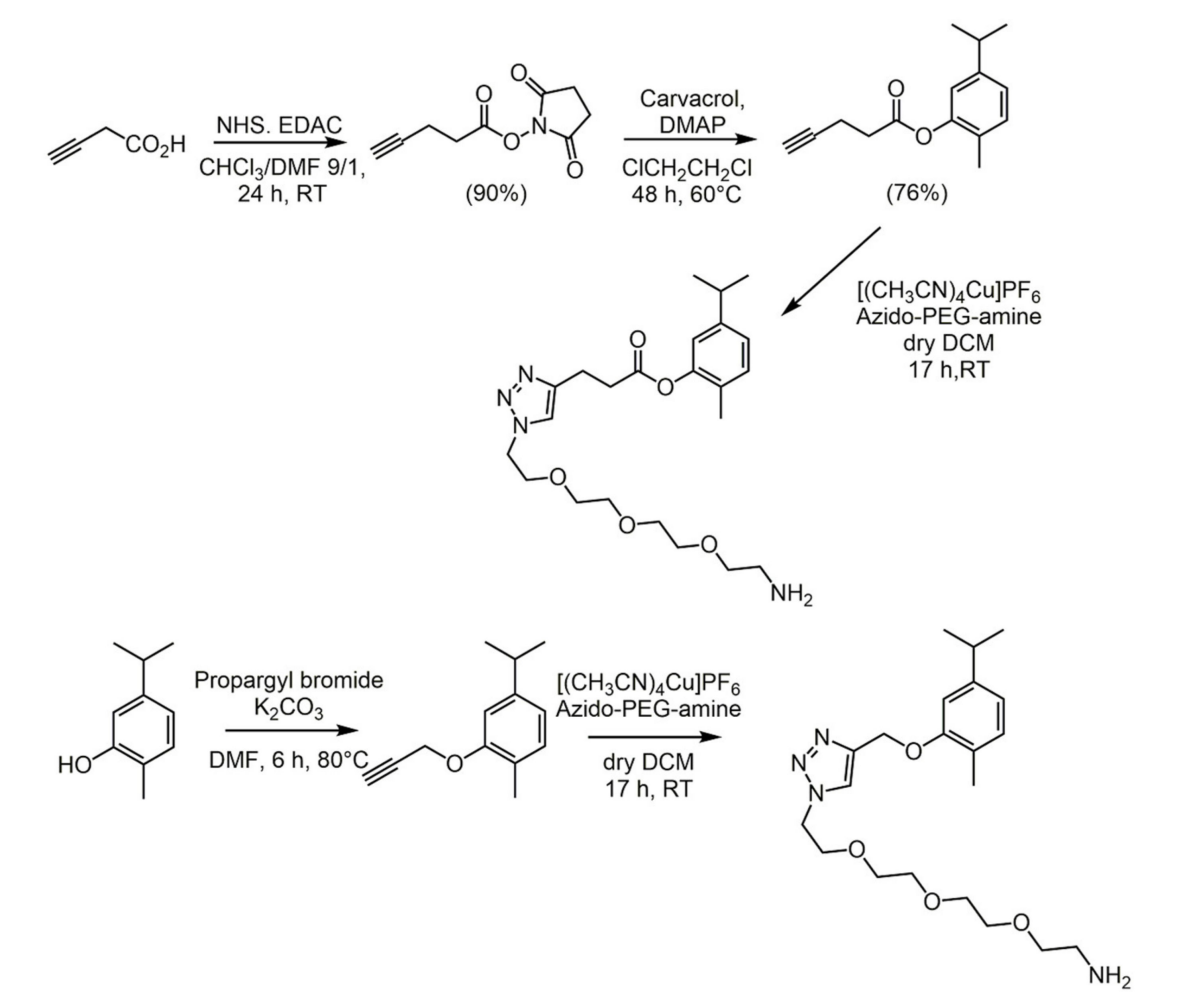
Synthesis of carvacrol ester and ether with a -NH_2_ terminal group [104]. Abbreviations: NHS = *N*-hydroxysuccinimide; EDAC = *N*-(3-dimethylaminopropyl)-*N*′-ethylcarbodiimide hydrochloride; Azido-PEG-amine = *O*-(2-aminoethyl)-*O*′-(2-azidoethyl)triethylene glycol.

A more advanced approach was related to the carvacrol anchoring on a gold surface, to develop antimicrobial coats [104]. Indeed, carvacrol functionalization at the phenolic group was performed to obtain a carvacrol ester and an ether, with a -NH_2_ terminal group (Scheme 5). The latter could be covalently attached to a properly modified gold surface. Thus, the antifungal activity of carvacrol functionalized Au surfaces was evaluated against *C. albicans* and more than 75% of inhibition was observed for the ester derivative, while 65% inhibition was reached with the ether one. Noteworthy, the fungicidal activity was maintained after being stored for one month at 4 °C.

**Scheme 6:**
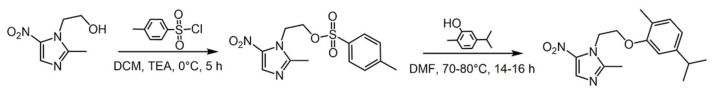
Synthesis of a metronidazole carvacrol ether derivative [108].

Next to carvacrol ester derivatives, ethers have also been extensively studied over the years to implement carvacrol applications [105,106]. In particular, several carvacrol ethers have been explored in the treatment of *H. pylori* bacterial infection and as antiproliferative agents against human gastric adenocarcinoma cell lines, with promising results [107]. Similarly, a metronidazole carvacrol ether derivative has shown remarkable activity against two strains of *H. pylori* and one strain of *Clostridium perfringens* (Scheme 6) [108].

**Scheme 7:**
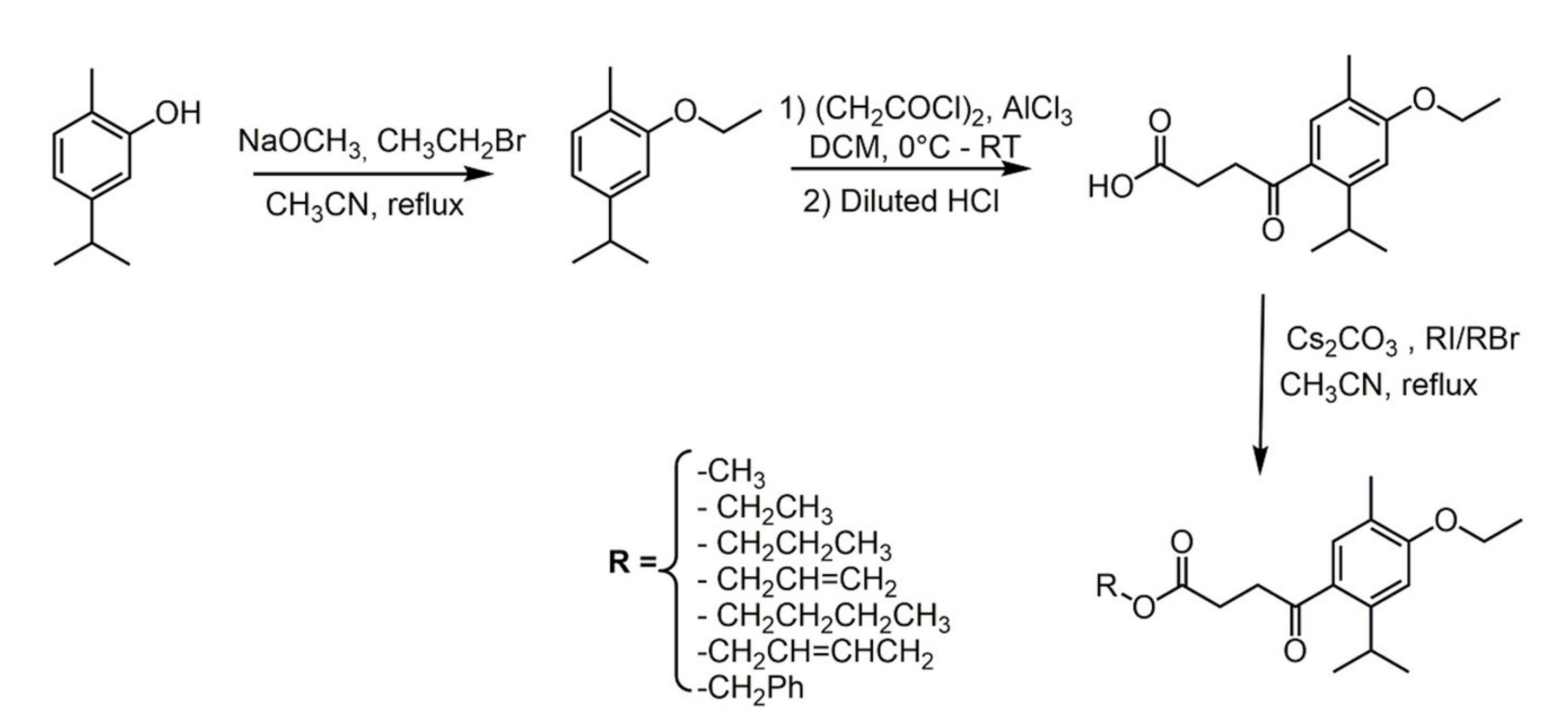
Synthesis of alkyl 4-oxobutanoate *p*-substituted carvacryl ethyl ethers [110].

Carvacrol propyl, butyl, octyl and benzyl ethers demonstrated the ability to reduce fertility and viability of the fruit fly *Drosophila melanogaster* after oral administration or inhalation exposure [109]. Moreover, different alkyl 4-oxobutanoate *p*-substituted carvacryl ethyl ethers have been synthesized (Scheme 7) and screened as tyrosinase inhibitors, which are valuable molecules in medicine, agriculture and cosmetics because of their ability to control melanin overproduction [110]. Data showed that synthetic ethers were more effective in inhibiting tyrosinase with respect to the parent compound.

**Scheme 8:**
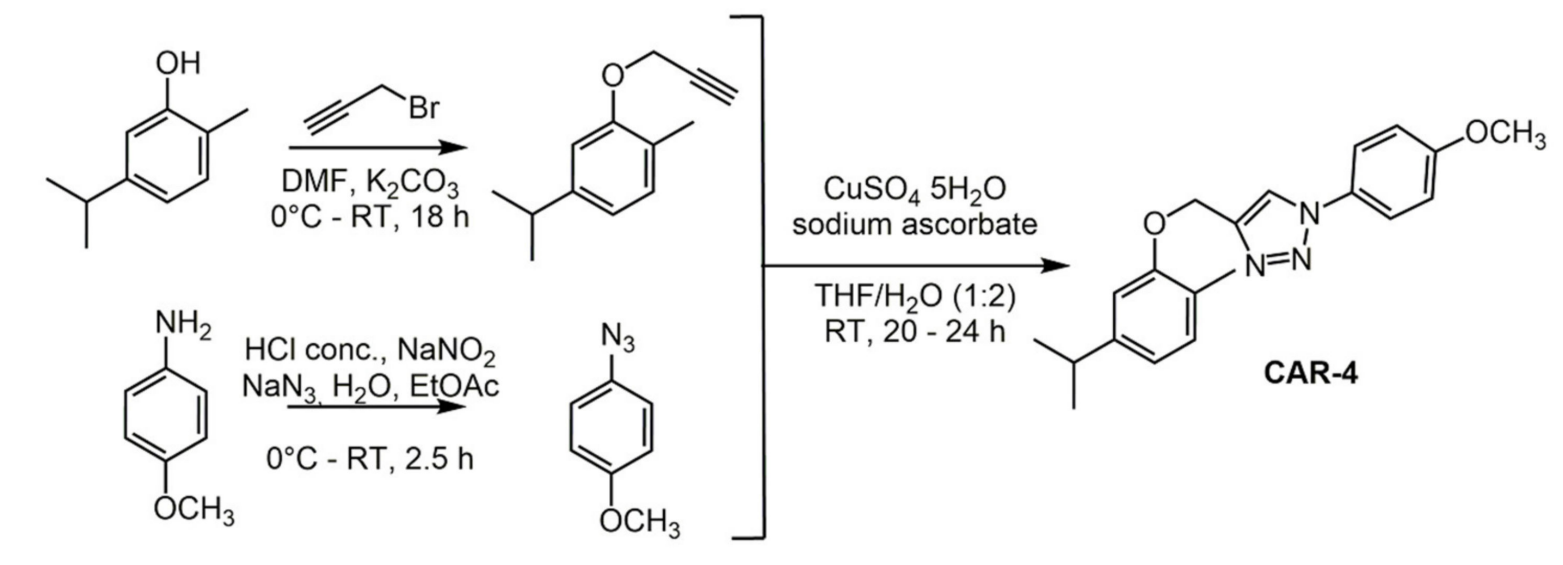
Synthesis of **CAR-4** [111,112].

Docking studies demonstrated that carvacrol derivative **CAR-4** (Scheme 8) is a promising anti-malaria agent [111]. In particular, **CAR-4** interacts with amino acid residues in the binding pocket of the protease of *P. falciparum* parasite, a common target for anti-malarian drugs. Therefore, **CAR-4** was synthesized starting from carvacrol and propargyl bromide in the presence of K_2_CO_3_. The resulting alkyne was reacted with *p*-methoxyphenylazide in the presence of a Cu(I)-salt and sodium ascorbate in THF/H_2_O, to form the [3 + 2] cycloaddition product (Scheme 8). **CAR-4** showed high anti-malarian activity, with IC_50_ value of 8.8 μM. In vivo tests showed significant parasite reduction up to 8 days, making **CAR-4** a potential lead against the target protease.

**Scheme 9:**
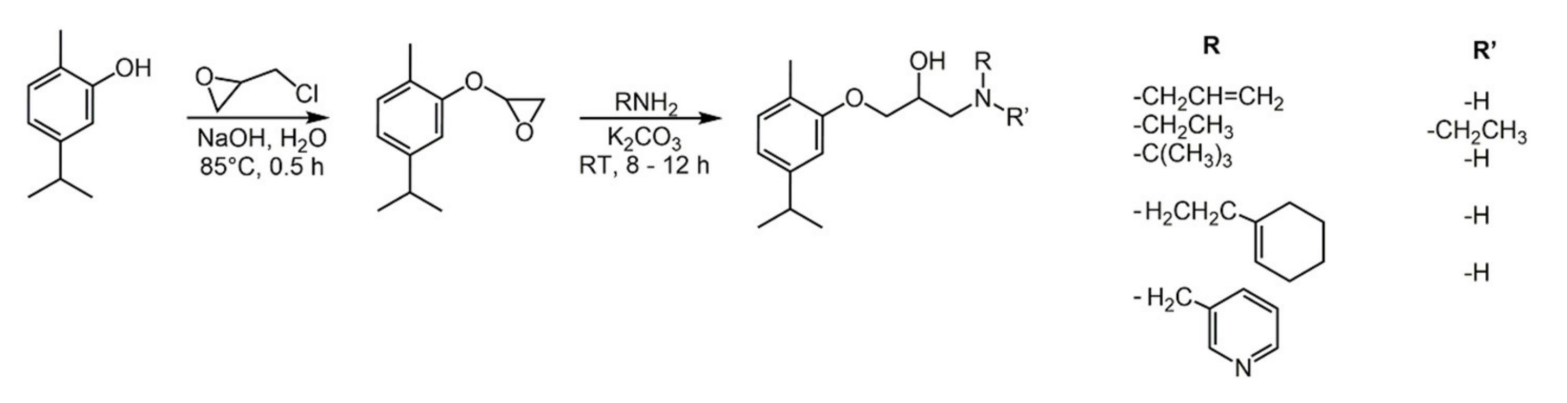
Synthesis of oxypropanolamine carvacrol derivatives [113].

Oxypropanolamine derivatives of carvacrol were tested to evaluate their application in different diseases (Scheme 9) [113]. In particular, their inhibitory effects towards different type of carbonic anhydrase, *α*-glycosidase and acetylcholinesterase enzymes have been evaluated and results showed a very good inhibition effect, even higher than that of reference compounds. Therefore, such synthetic carvacrol derivatives can be further exploited as diuretics, antiepileptics, anti-glaucoma, anti-diabetic and anti-inflammatory agents in the treatment of gastric and duodenal ulcers and in neurological disorders, such as Alzheimer’s disease.

**Scheme 10:**
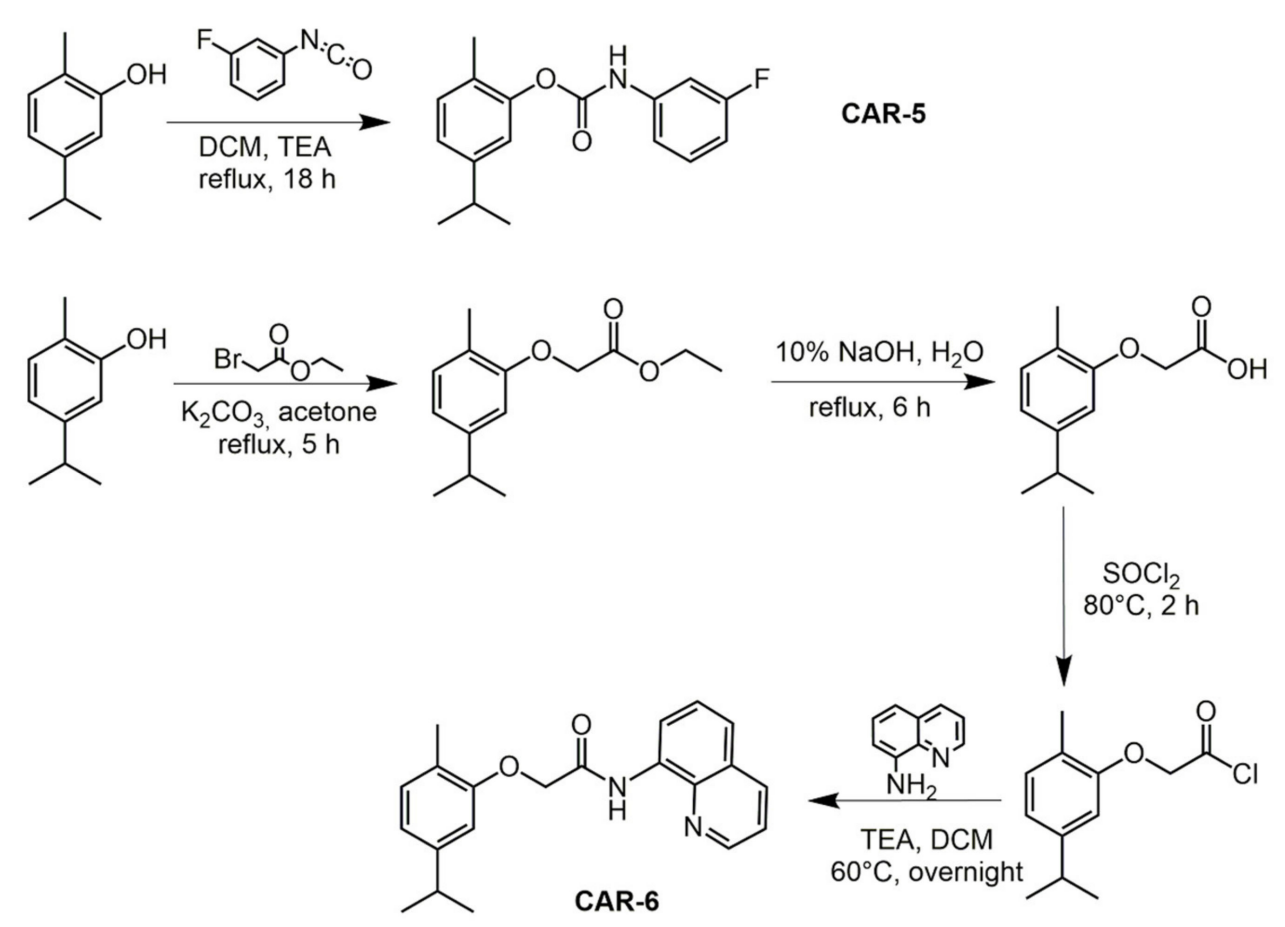
Synthesis of **CAR-5** [114] and **CAR-6** [115].

3-Fluorophenyl carbamate derivative of carvacrol (**CAR-5**, synthesized from reaction between carvacrol with 3-fluorophenyl isocyanate in DCM, Scheme 10) is 130-fold more active compared to carvacrol in acetylcholinesterase inhibition and 400-fold more efficient in inhibiting butyrylcholinesterase, with negligible cell death [114]. More interestingly, a series of carvacrol amide derivatives have been screened against acetylcholinesterase and butyrylcholinesterase enzymes [115]. The carvacrol derivative modified with a quinoline moiety (**CAR-6, Scheme 10**) is 149-fold more effective than carvacrol in inhibiting acetylcholinesterase and more than 8000-fold more efficient for butyrylcholinesterase inhibition. The higher activity compared to carvacrol was related to the presence of the heterocyclic aromatic quinoline core that can interact with the amino acid residues in the active site of the enzyme through π–π interactions.

Sulfonic acid functionalized carvacrol, synthesized through the electrophilic aromatic sulfonation reaction with concentrated H_2_SO_4_, and the corresponding potassium salt, are less effective anti-bacterial agents with respect to carvacrol, but their remarkable water solubility and reduced odor allow their use in the food industry for preservation of foodstuffs and for shelf life increase [116]. A series of interesting differently substituted carvacrol analogues, such as sulfonate esters [117] (obtained by reaction with ethane sulfonyl chloride or aryl sulfonyl chloride in dichloromethane, in the presence of TEA), dihydroxy- [118], acetohydrazone- [119], hydrazone-, sulfonyl hydrazone- [120], and hydrazide-based sulfonamide- [121] carvacrol derivatives have been synthesized and screened for their antimicrobial, antioxidant, and anti-cancer activities. Results suggest that the synthesized compounds exhibit very promising biological properties in the studied fields, even though their efficiency was not directly compared with that of carvacrol.

**Scheme 11:**
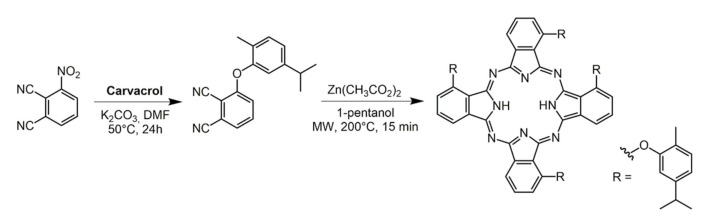
Synthesis of **CAR-7** [122].

In a recent paper, carvacrol has been successfully coupled with phthalocyanines [122]: 3-nitrobenzene-1,2-dicarbonitrile was initially reacted with carvacrol and then the resulting compound was subjected to macrocyclization under MW irradiation, to obtain the corresponding phthalocyanine, **CAR-7** (Scheme 11).

The photodynamic antibacterial activity of the synthesized phthalocyanine was evaluated: upon excitation with light, the carvacrol-substituted phthalocyanine showed an increased photoinactivation at 100 μM with respect to the sole zinc(II) phthalocyanine. A lower dark toxicity was observed in comparison to pure carvacrol, likely because of the lower bacterial membrane penetration of bulk phthalocyanine with respect to carvacrol. Nonetheless, lower photostability of the conjugate was observed [122].

### 2.2. Thymol

Next to carvacrol, its isomer, thymol, is widely used as an antibacterial, antifungal, antioxidant and anti-inflammatory active ingredient in several products, as well as a food preservative [84,123].

Indeed, several natural and synthetic thymol derivatives have been proposed over the years to further broaden its application at the industrial level [124,125,126].

Quite a few thymol derivatives have been synthesized and evaluated for different biological purposes [86,127,128,129,130]. Thymol functionalization through esterification or etherification reactions constitutes one of the most useful approaches to access a wide library of different bio-active molecules. Thymol esterification usually occurs in the classical conditions, reacting thymol with the appropriate anhydride or acyl chloride in the presence of a base. MW-assisted procedures in aqueous medium have also been proposed, to perform reactions in reduced times and with improved yields [131].

Thymol acetylation has been widely studied, since the product, i.e., thymyl acetate, is more effective than thymol against plant pathogenic fungi, such as *A. solani*, *B. cinerea*, *P. grisea* and *R. solani* [89] and Gram-positive bacterial strains such as *S. mutans*, *B. subtilis* and *S. epidermidis* [97]. Higher or equal activity with respect to thymol was assessed for Gram-negative *E. coli*, *S. typhimurium*, *P. aeruginosa* and *K. pneumonia* [132]. Similar enhancements in anti-bacterial action have been achieved with thymyl propanoate and methylpropanoate derivatives [97], while thymol esterification with heteroaromatic carboxylic acids led to efficient anti-fungal compounds [99]. Moreover, in a screening of different synthetic thymyl esters and ethers, thymyl benzoate exhibited the highest larvicidal potency on *Aedes aegypti*, which is a dangerous mosquito and vector of dengue fever and other diseases in the world [133]. Importantly, thymol protection through esterification proved to be effective to reduce thymol toxicity. In fact, thymol acetate and benzoate are promising candidates as antileishmanial drugs, being less toxic and more active than thymol against the parasite *Leishmania infantumchagasi* [134]. Thymol acetylation has been considered effective also in the treatment of gastrointestinal nematode infection of small ruminants because of the reduced toxicity of the ester compared to the parent compound, even though thymol acetate was actually less effective than thymol in vitro studies [135].

**Scheme 12:**
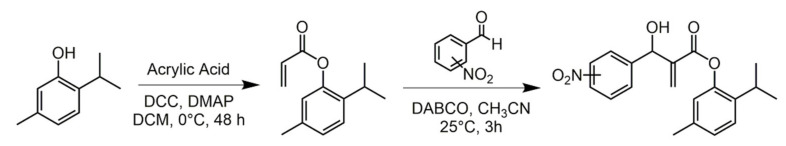
Synthesis of thymol acrylate derivatives [136].

Acrylate derivatives of thymol have been synthesized according to a multi-step process (Scheme 12) [136].

Thymol esterification with acrylic acid in the presence of DCC and catalytic amount of DMAP in DCM was performed. The obtained product was reacted with nitro-substituted benzaldehyde in dry acetonitrile, with the nucleophilic catalyst 1,4-diazabicyclo[2.2.2]octane (DABCO). Reactions proceeded with good yields, and product with higher antileishmanial activity than thymol against *Leishmania amazonensis* was obtained [136].

**Scheme 13:**
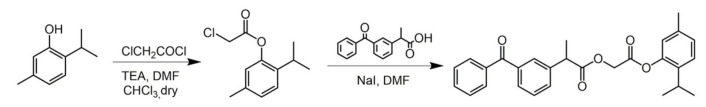
Synthesis of thymol ketoprofen derivative [139].

Promising results have also been achieved with the conjugation of nonsteroidal anti-inflammatory drugs (NSAIDs) to thymol, to prevent adverse gastrointestinal mucosal reactions, which are typical side effects related with long-term use of NSAIDs [137,138,139,140]. The formation of gastric ulceration related with NSAID therapy is usually due to the local generation of reactive oxygen species (ROS); thus, the introduction of antioxidant components in NSAIDs’ structure can limit such undesired effects. Accordingly, thymol esterification product with indomethacin, etodolac and tolfenamic acid showed retention of pharmacological activity with respect to the parent drug and significant reduction in ulcerogenic side effects of the corresponding NSAID [137]. Similarly, thymol has been included in ketoprofen drug (2-(3-benzoylphenyl)propanoic acid), through a glycolic acid spacer (Scheme 13) [139].

Modified ketoprofen showed better analgesic and anti-inflammatory activities and reduced gastrointestinal toxicity, thus demonstrating the great advantage in using such prodrugs for chronic inflammatory disorders treatment.

Thymol conjugation with diacerein (1,8-diacetoxy-3-carboxyanthraquinone), an anthraquinone derivative used as antiarthritic, moderate anti-inflammatory, antipyretic and analgesic drug, has been exploited. Diacerein linkage to thymol through esterification with DCC improved lipophilicity and bioavailability of the drug, while decreasing gastric irritant effect and enhancing anti-inflammatory activity [140].

**Scheme 14:**
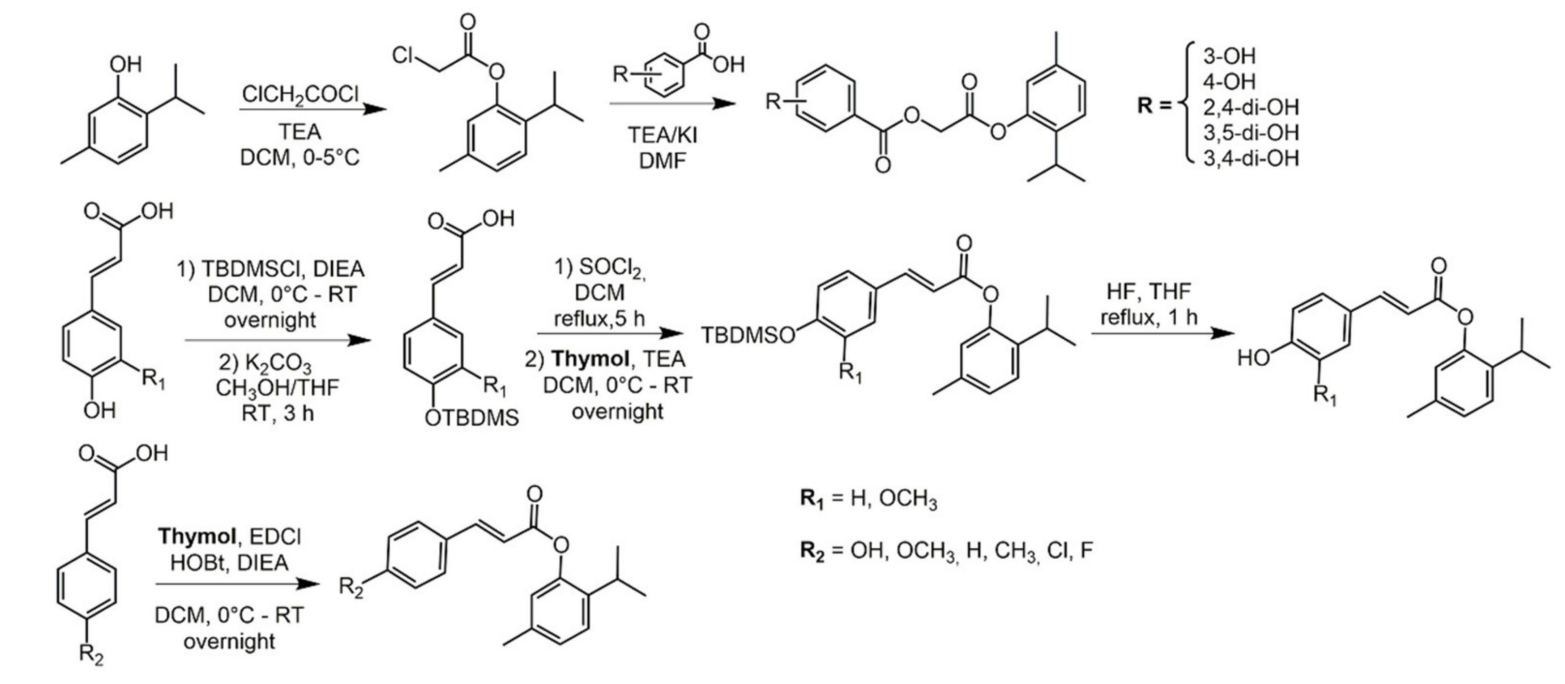
Synthesis of benzoic acids [141] and cinnamic acids [144], derivatives of thymol. Abbreviations: TBDMSCl = *tert*-butyldimethylsilyl chloride; DIEA = *N*,*N*-diisopropylethylamine; EDCI = 1-etil-3-(3-dimetilaminopropil)carbodiimide; HOBt = hydroxybenzotriazole.

Because of their already known antioxidant and mushroom tyrosinase inhibitory activity, substituted benzoic acids and cinnamic acids bearing a thymol moiety have been synthesized in order to discover new effective tyrosinase inhibitors [141,142,143,144]. To this aim, thymol esterification has been carried out, with properly substituted benzoic or cinnamic acids, in the presence of TEA (Scheme 14).

Among the tested compounds, derivatives possessing 4-hydroxyl substituted cinnamic acid are the most active ones, having the maximum binding affinity with the receptor protein [141,144]. Thus, the synthesized derivatives may serve as lead structures for developing even more effective tyrosinase inhibitors.

2-Isopropoxy-1-isopropyl-4-methylbenzene, obtained by thymol etherification with 2-chloropropane in the presence of TEA in diethyl ether, exhibited an enhanced antibacterial activity with respect to thymol against *E. coli*, *S. typhimurium*, *S. aureus*, *P. aeruginosa* and *K. pneumonia* [132]. Interesting thymol oxypropanolamine derivatives showed powerful antibacterial activity on different Gram-negative and Gram-positive bacteria, as well as good inhibition of some metabolic enzymes, such as human carbonic anhydrases isoenzymes I and II, *α*-glycosidase and acetylcholinesterase [145]. Enhancements of the thymol biological activity have also been detected with thymol glucosides.

**Figure 1:**
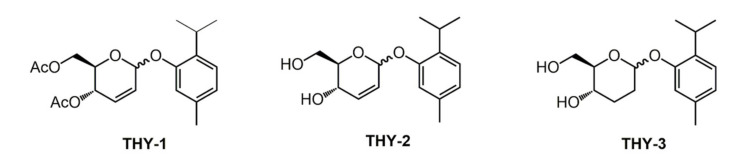
Structure of the glucosides thymol derivatives **THY-1**, **THY-2**, **THY-3** [149].

Glycosylation is a versatile method that allows us to improve the hydrophilicity of organic compounds, also widening their pharmacological applications. Several thymol glucosides derivatives have been synthesized and tested over the years [146,147,148]. Notably, the in vitro antifungal activity of 2-isopropyl-5-methylphenyl-4,6-di-*O*-acetyl-2,3-dideoxy-*α*-*D*-erythro-hex-2-enepyranoside (**THY-1**), 2-isopropyl-5-methylphenyl-2,3-dideoxy-*α*-*D*-erythrohex-2-enepyranoside (**THY-2**) and 2-isopropyl-5-methylphenyl-2,3-dideoxy-*α*-*D*-erythrohexanopyranoside (**THY-3**) (Figure 1) has been evaluated, and larger inhibition zones and lower MIC were achieved against *A. flavus*, *A. ochraceus* and *F. oxysporum* compared to thymol. Thus, because of the improved hydrophilicity, next to the biological activity, glucosides thymol derivatives can be suggested as antifungal agents in food systems [149].

**Scheme 15:**
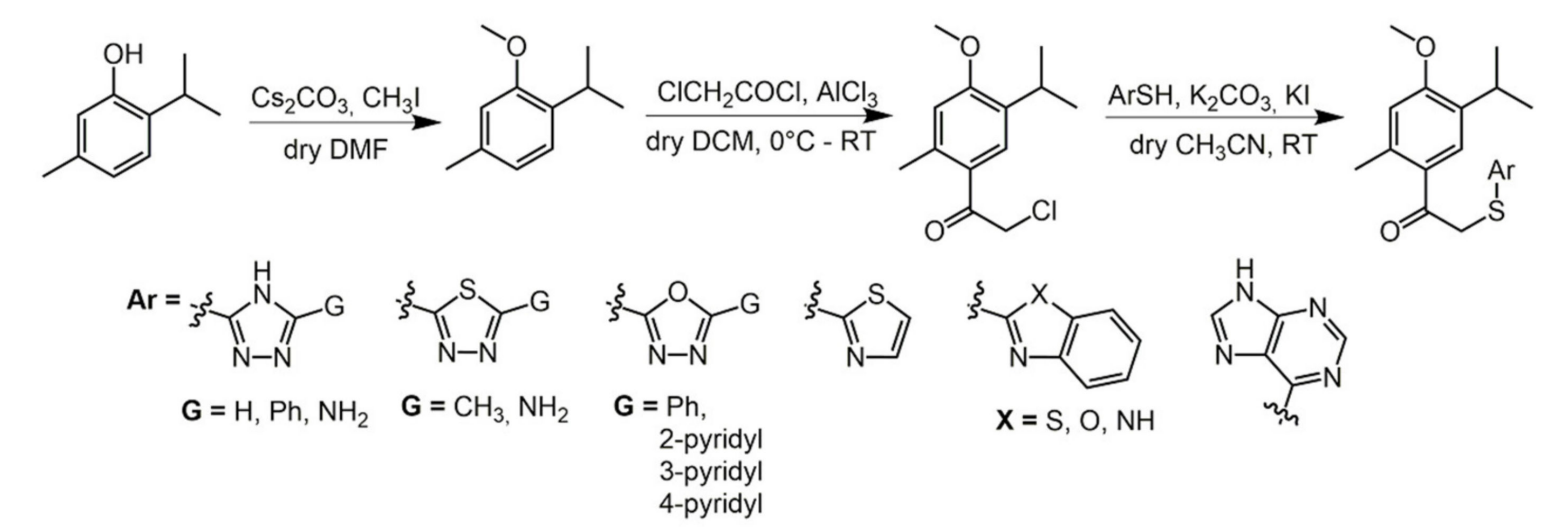
Synthesis of heterocyclic sulfide thymol derivatives [150].

The antioxidant activity of a series of heterocyclic sulfide derivatives of thymol has been recently exploited [150]; they were prepared following a multi-step procedure (Scheme 15), where the natural phenol was firstly subjected to methylation reaction with cesium carbonate and methyl iodide in DMF. To note, DMC can efficiently replace CH_3_I in thymol methylation reaction [151]. Next, Friedel–Crafts acylation of the methyl-protected thymol with chloroacetyl chloride led to 2-chloro-1-(5-isopropyl-4-methoxy-2-methylphenyl)ethan-1-one in 48% yield. Nucleophilic substitution of Cl- was then performed, with the proper heterocyclic aromatic thiols, in the presence of potassium carbonate and potassium iodide in acetonitrile, to obtain the desired thymol derivatives.

Synthesized compounds showed good anti-oxidant activity, and docking studies on tyrosinase revealed higher affinity with respect to thymol and the reference compound (kojic acid) toward the binding site of the enzyme. In particular, the oxadiazole derivatives presented the largest binding affinities with the enzyme because of favorable H-bond interactions with amino acid residues in the active site [150].

Recently, new thymol sulfonamide derivatives have been prepared. The synthetic pathway firstly requires diazonium salt synthesis from the aromatic amine, and then electrophilic substitution on thymol aromatic ring, in basic solution. The conjugate thymol-sulfadiazine derivative is the most antibacterial active one, showing inhibitory activity against *S. aureus* and *E. faecalis* [152]. Differently functionalized thymol derivatives have been obtained, such as thymol-based paracetamol analogues [153] or aryl-azo-substituted thymol [154], as well as *N*-methylcarbamate derivatives [155,156] and they showed good anti-oxidant and anti-microbial activities. A 1,3,5-triazine piperazines thymol derivative displayed very interesting therapeutic perspectives as a drug against memory and cognitive impairment, i.e., Alzheimer’s disease and dementia, having good pharmaceutical and safety profiles in vitro [157]. Increased antibacterial and antifungal activity has been observed for thymol pyridazinone [158] and thymol pyridine [159] derivatives, with respect to thymol, while 2-(4*H*-1,2,4-triazole-3-yl)thioacetamide thymol derivatives exhibited promising anti-cancer activity [160]. Mannich bases of thymol were investigated as carbonic anhydrase inhibitors, showing moderate activity [161].

**Scheme 16:**
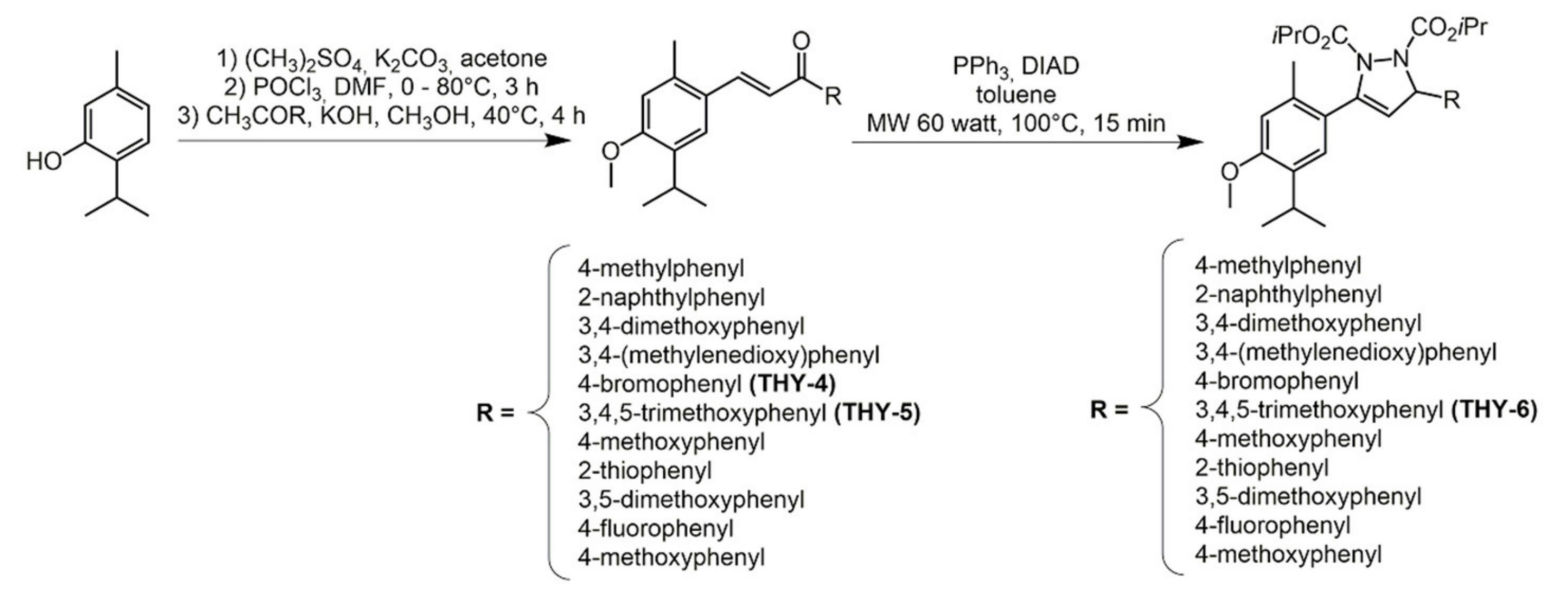
Synthesis of thymol-based substituted pyrazolines and chalcones [162].

Thymol-based substituted pyrazolines and chalcones have been tested against human malaria parasite strain *Plasmodium falciparum* activity [162]. The proposed synthetic pathway to access such bioactive compounds firstly requires the synthesis of 3-isopropyl-4-methoxy-6-methylbenzaldehyde. Chalcones are then obtained through Claisen–Schmidt condensation of the aldehyde with different acetophenones in methanol, with KOH excess. Reaction of the thymol-based chalcones with diisopropyl azodicarboxylate (DIAD) in the presence of PPh_3_ and toluene afforded the functionalized pyrazolines, under MW irradiation, with good yields (Scheme 16).

The synthesized compounds showed enhanced anti-malarian activity with respect to thymol, and in particular, chalcones **THY-4** and **THY-5** and pyrazoline **THY-6** exhibited highest activity against human malaria parasite *P. falciparum*, being much more effective than the parent compound [162].

Different studies also demonstrated that halogenation is a proficient strategy to enhance thymol biological activity. Yet, thymol chlorination affords 4-chlorothymol as a main product [163,164], which is up to six times more active than thymol against *S. aureus*, *S. epidermis* and different *C. albicans* strains [163]. More interestingly, thymol bromination in mild conditions leads to 4-bromothymol [165,166,167], which is a very effective antimicrobial active compound [168]. In fact, its activity is up to 15 times stronger than that of the parent compound, against several bacterial and fungal strains pathogenic for humans and animals. Thus, 4-bromothymol sustainable synthesis was the object of several studies [167], and biocompatible drug delivery methods have been also developed to study the potential application of such an interesting antimicrobial compound for topical applications in cosmetics [151].

### 2.3. Eugenol

**Figure 2:**
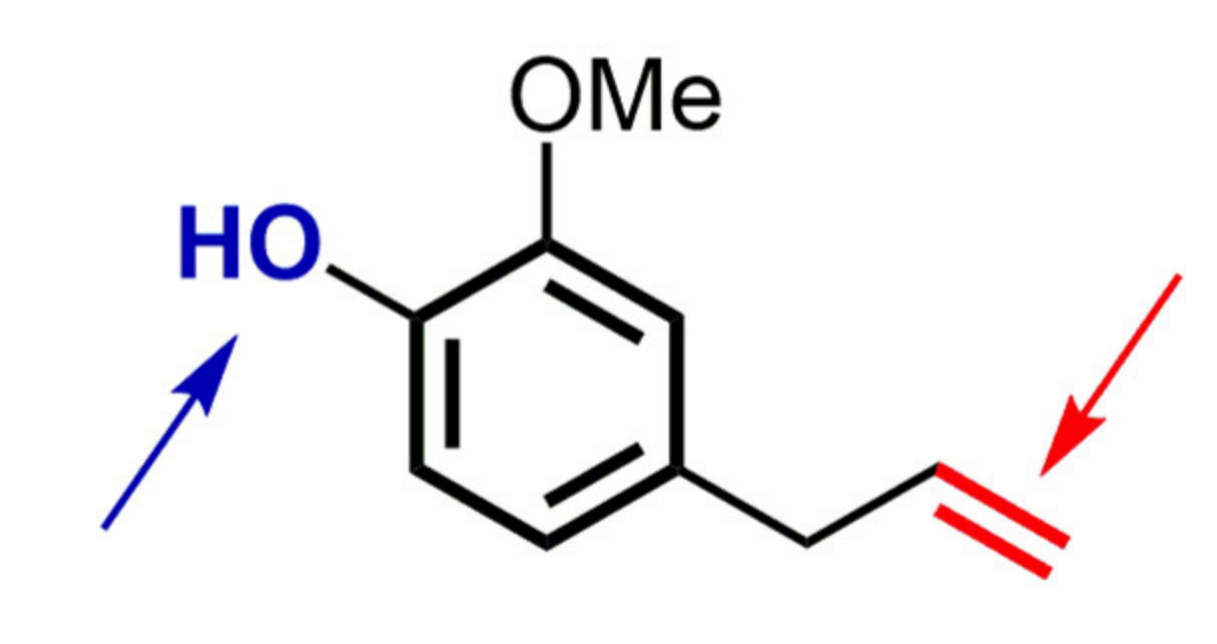
Eugenol structure.

Eugenol (4-allyl-2-methoxyphenol) is the major component of clove essential oils, but it can be also found in minor amounts in cinnamon, clover pepper and other plants. It is used in perfumeries for its pleasant fragrance, as a flavoring agent in foods, as antiseptic and disinfectant in dental products and in many other fields [169]. Eugenol can be readily functionalized through the chemical transformation of the phenolic -OH group (mainly via the classical etherification and esterification reactions) [170,171,172,173,174,175,176], on the aromatic ring (through nitration reaction or Mannich bases formation) [177,178,179,180], as well as on the allylic functionality, through epoxidation [175] (Figure 2).

**Figure 3:**
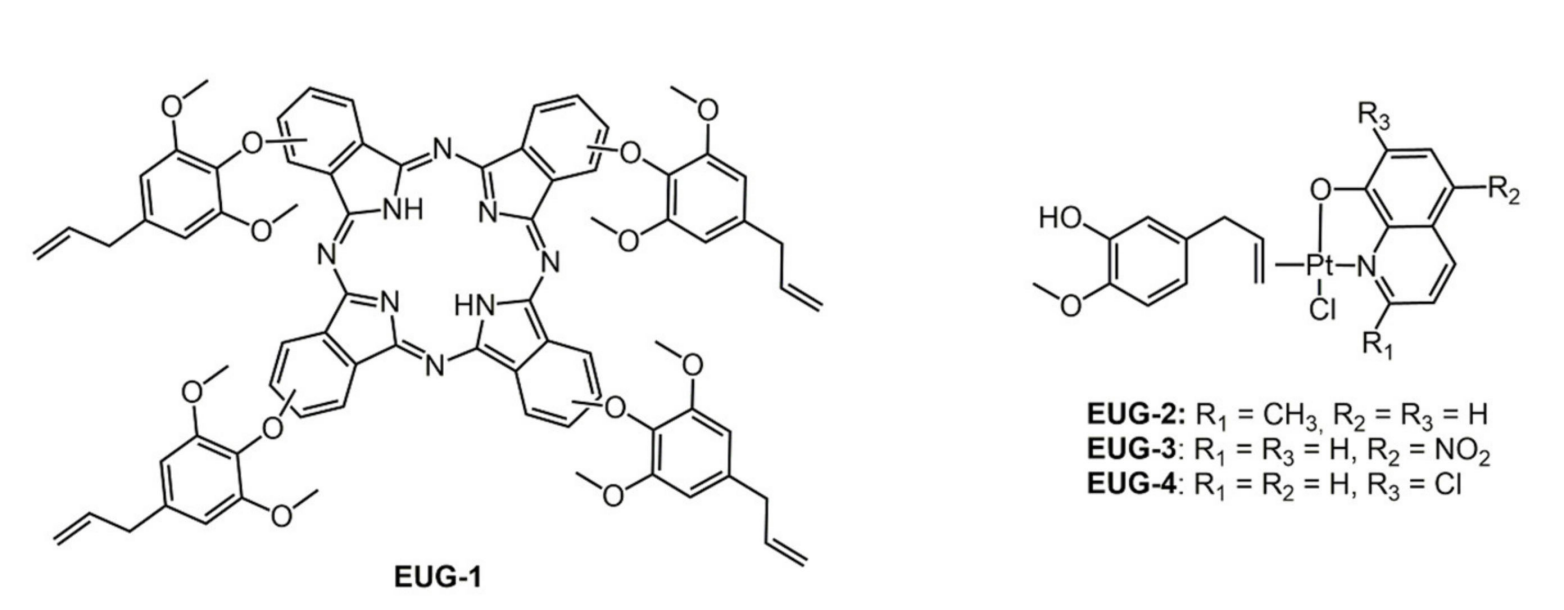
Structure of eugenol phthalocyanine derivative (**EUG-1**) [185] and eugenol Pt(II) complexes **EUG-2-4** [186].

Thanks to its highly versatile structure, several eugenol derivatives have been synthesized for different biological purposes in the past ten years [181,182]. Moreover, eugenol can be used as a scaffold to synthesize biologically active natural products [183,184]. The possibility to introduce eugenol skeleton in complex structures such as phthalocyanines [185], platinum(II) complexes [186], and organic antimicrobial polymers [187] has been explored, obtaining new interesting biologically active species (Figure 3).

Alkyl and aryl eugenol esters have promising anti-inflammatory agents for skin inflammation [188], anti-oxidants [189], as well as effective antibacterial and anti-fungal compounds [190]. In particular, different eugenol esters with high anti-oxidant activity have been synthesized for application in cosmetics. Results showed that after esterification, skin penetration of the active compounds was increased; therefore, eugenol ester derivatives could explicate their anti-oxidant activity in the deeper layers of the skin [191]. Eugenol tosylate derivatives have also been synthesized by reaction with different sulfonyl chlorides in the presence of pyridine. The obtained tosylates are effective inhibitors for *Candida albicans* [192,193,194].

Eugenol esterification has been also performed with aspirin (acetylsalicylic acid, previously activated with SOCl_2_ to form the corresponding acyl chloride). The obtained ester is a very promising compound, having fewer toxic effects than aspirin and eugenol [195] and showing interesting therapeutic effects [196,197,198]. In fact, it is an anti-inflammatory and antipyretic drug, with stronger and longer effects than its precursors, likely indicating a synergistic effect between the two moieties [195]. Moreover, eugenol esterification with ibuprofen led to a prodrug with retention of anti-inflammatory activity and minimized gastrointestinal toxicity [199].

**Scheme 17:**
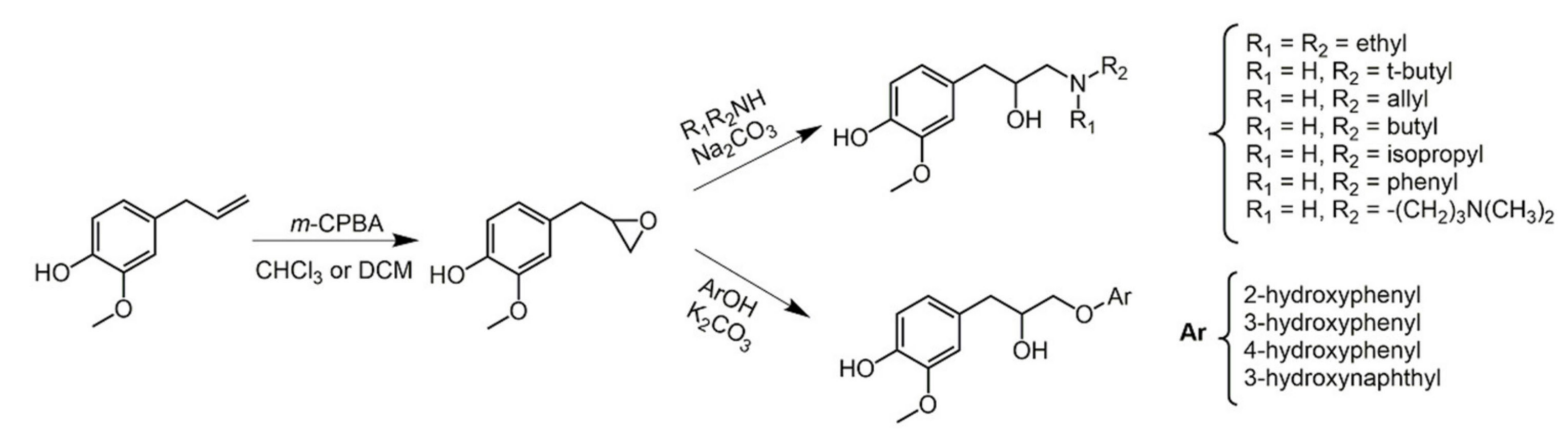
Eugenol derivatives obtained by epoxidation and ring opening reactions [200,201].

Eugenol epoxidation at the allylic position, followed by ring opening with different nucleophiles, gives access to a wide library of eugenol derivatives that have been tested as carbonic anhydrase, acetylcholinesterase and *α*-glycosidase inhibitors, with good results (Scheme 17) [200,201]. Oxypropanolamine derivatives, obtained by ring opening with amines, showed antibacterial activity on Gram-negative (*A. baumannii*, *P. aeruginosa* and *E. coli*) and Gram-positive (*S. aureus*) bacteria [202].

**Figure 4:**
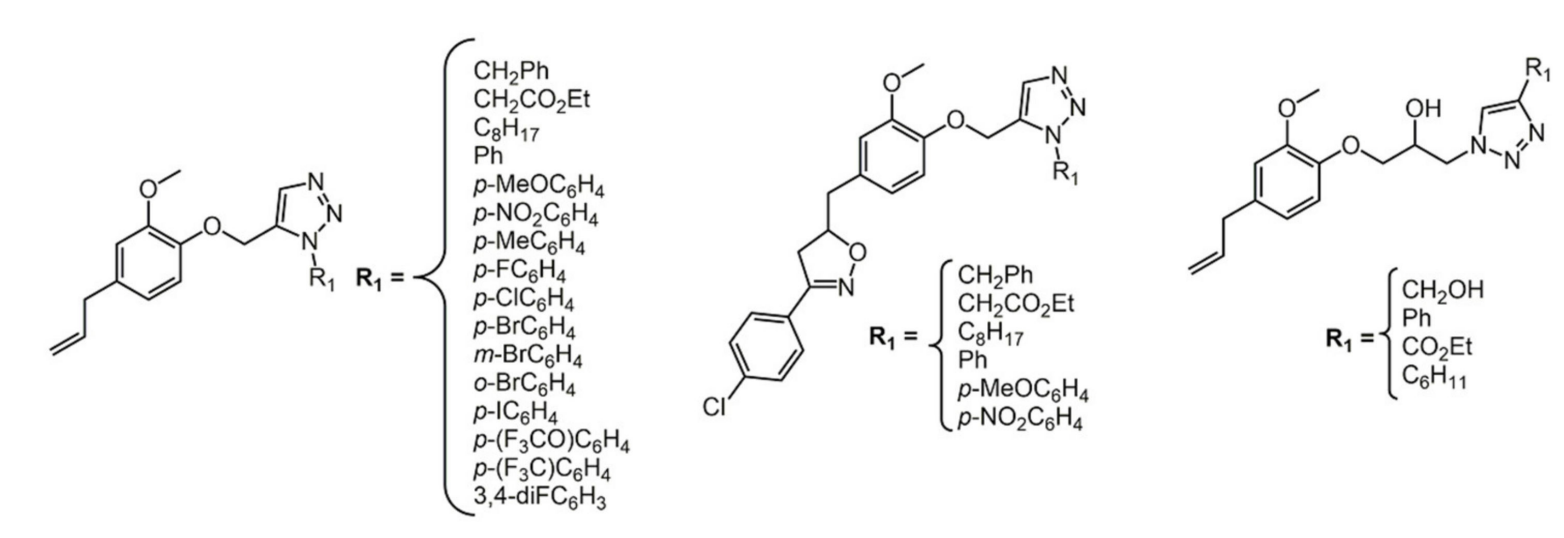
Structure of different eugenol triazole derivatives [203,204,205,206].

**Scheme 18:**
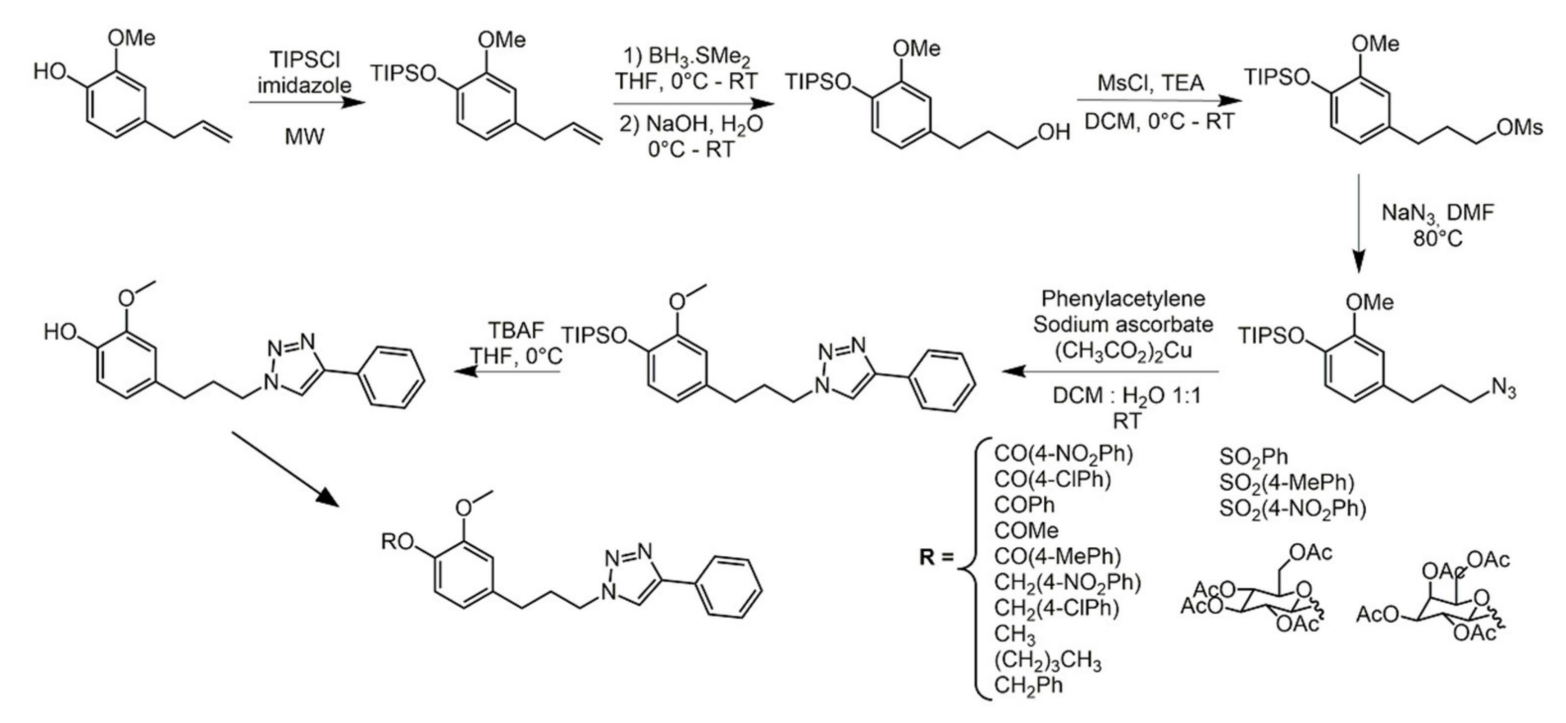
Synthesis of different eugenol triazole derivatives [207]. Abbreviations: TIPSCl = triisopropylsilyl chloride; TBAF = tetrabutylammonium fluoride.

Numerous eugenol derivatives bearing triazole functionalities have been successfully accessed through the “click chemistry” approach (Figure 4, Scheme 18). Several examples are present in the literature about (*i*) *O*-alkylation of eugenol with terminal alkynes, followed by reaction with different benzyl azides [203,204,205]; (*ii*) eugenol conversion in epoxide and ring opening to obtain the corresponding alkyl azides, followed by reaction with different alkynes [206]; (*iii*) hydroboration oxidation at the allylic position of eugenol, followed by mesylation and azidation reactions, to achieve the eugenol azide; then, reaction with phenylacetylene to afford the triazole. Importantly, the first step of this latter process requires -OH protection trough silylation, thus allowing the synthesis of variously substituted products (Scheme 18) [207].

Synthesized eugenol triazole derivatives showed leishmanicidal [205], antimycobacterial [207], trypanocidal [206], anticancer [203] as well as protease inhibitory [204] activity. Triazole eugenol glucosides also showed significant bactericidal activity and low toxicity to normal cells [208].

**Scheme 19:**
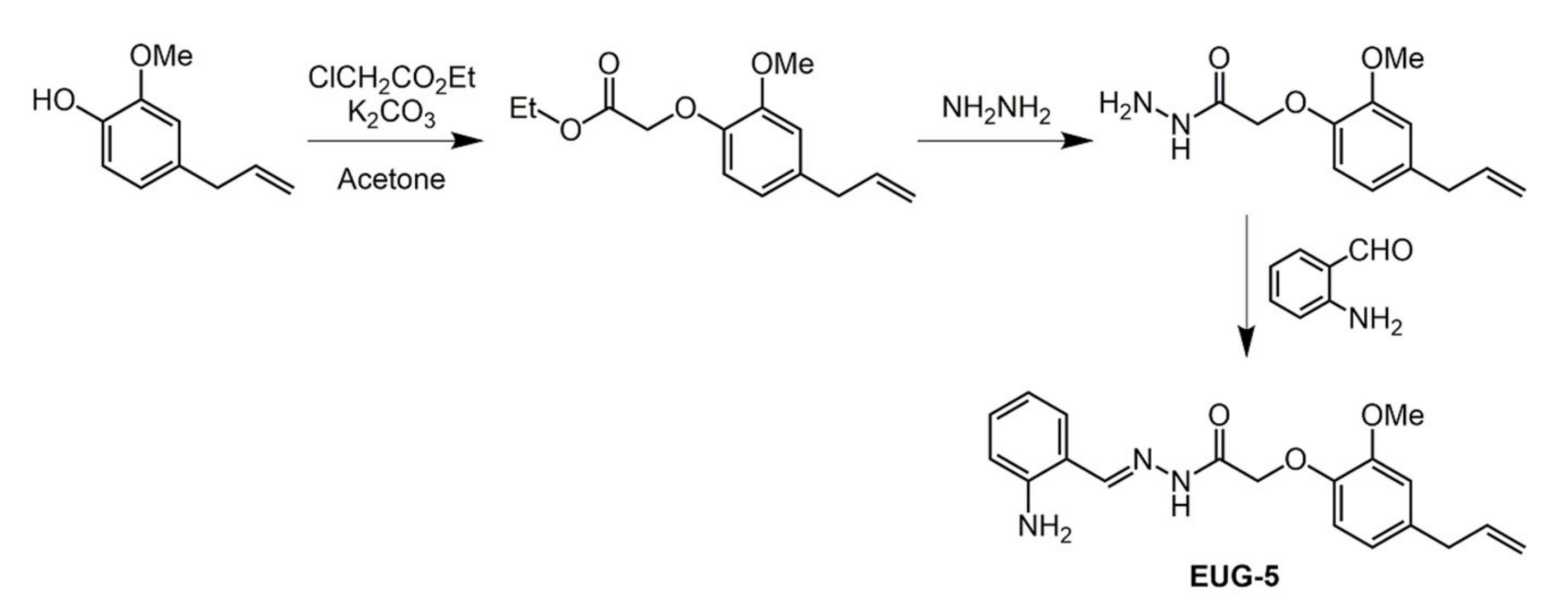
Synthesis of **EUG-5** [209].

A series of hydrazones of eugenol have been recently synthesized by condensation of a eugenol hydrazide with various aromatic aldehydes or ketones (Scheme 19) [209]. All the obtained hydrazones showed a promising antitubercular activity, measured by in vitro antimycobacterial activity test against *M. tuberculosis*. Docking studies revealed that the hydrazone eugenol derivative **EUG-5** interacts with the active site amino acid residues of the target enzyme, through the amino and phenyl functionalities.

Great attention has been recently devoted to new eugenol glucoside derivatives. Here, the synthesis is generally performed through a nucleophilic substitution reaction between the phenol group of eugenol and *α*-*D*-tetra-*O*-acetylglucopyranosyl bromide [210,211,212]. Some of the obtained derivatives showed strong anti-bacterial [211] and anti-fungal activity, mainly against different *Candida* species [210,212,213].

## 3. Diphenols

Natural diphenols, including catechol, resorcinol and hydroquinone derivatives, are widespread in nature, being commonly found in several vegetables and fruits. Such natural compounds are usually characterized by peculiar anti-oxidant and anti-inflammatory activity. Some of them have immunomodulatory and anticancer active ingredients. Therefore, natural diphenols are often used as scaffolds to prepare new efficient biologically active drugs. Although there is a widespread presence of bioactive diphenols in nature, in this review, the attention is dedicated to the tailored functionalization of resveratrol, hispolon and hydroxytyrosol, which constitute abundant and highly active natural phenolic compounds.

### 3.1. Resveratrol

Resveratrol (5-[(*E*)-2-(4-hydroxyphenyl)ethenyl]benzene-1,3-diol) is a natural phenolic stilbene, commonly found in several plants and fruits such as apples, berries, pomegranates, pistachios, as well as in the seed and skin of grapes. Thus, red wine is one of the highest sources of resveratrol [214]. Several studies are currently ongoing on resveratrol and its antibacterial [215], antiviral [216] and antitumoral [217,218,219] activities. Moreover, its applications in the treatment of diabetic nephropathy [220] and skin disorders [221] have been reviewed. The benefits associated with resveratrol, along with some adverse effects, mainly related to its potential cytotoxicity, have been recently highlighted [222]. Moreover, resveratrol’s rapid metabolism leads to low bioavailability of the active compound. Such issues, together with the low water solubility, constitutes an important limitation to be considered. Therefore, to improve bioavailability, several resveratrol delivery systems have been proposed, in order to extend the potential biomedical applications of such an interesting natural product [223].

Due to the significant attention on this active compound, several papers on its functionalization have been recently published; additionally, different reviews on the pharmacological and biological activity of resveratrol analogues and a dedicated Special Issue on its functionalization were published in 2017 [224].

**Figure 5:**
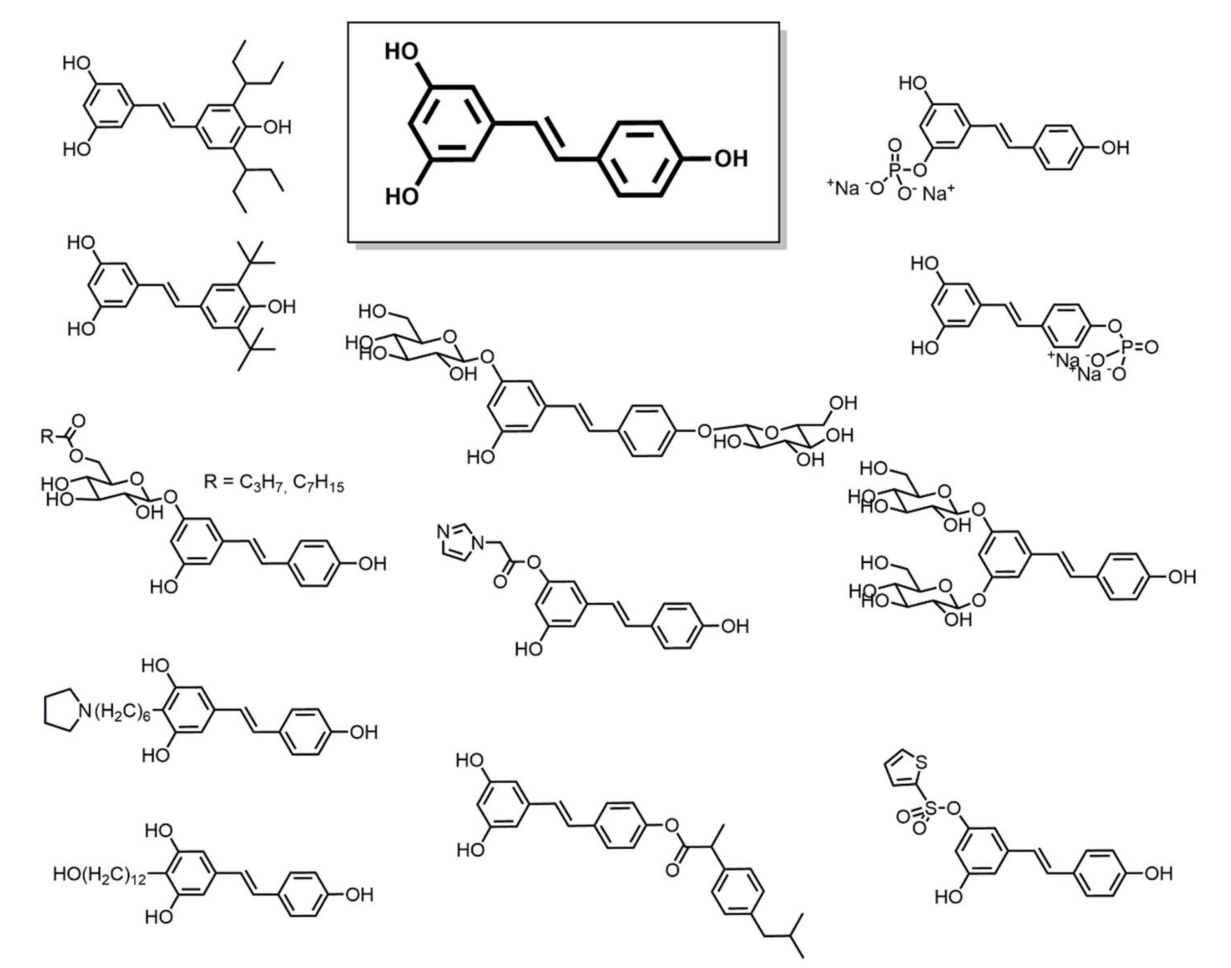
Structure of selected resveratrol derivatives.

Just to mention some examples (Figure 5), natural and synthetic resveratrol derivatives and oligomers are effective antibacterial [225] and antiviral [226] agents; di-, tetra- and hexahydroxy derivatives, as well as di-, tri-, tetra- and penta methoxy analogues exhibited higher bioavailability and biological activity with respect to resveratrol [219,225,226,227]; modification of resveratrol with carboxy ester, acetal, sulfonate, phosphate, carbonate, carbamate and alkyl groups allowed the modulation of resveratrol water solubility, bioavailability, absorption from the gastrointestinal tract and biological properties [228,229,230]. Resveratrol modification on the aromatic ring has also been investigated, leading to lipophilic derivatives, with anti-oxidant and neuroprotective activity [229]. To improve resveratrol anticancer activity, methoxy, hydroxyl derivatives, as well as other functional groups or heterocyclic esterification have been examined [231,232].

### 3.2. Hispolon

**Scheme 20:**
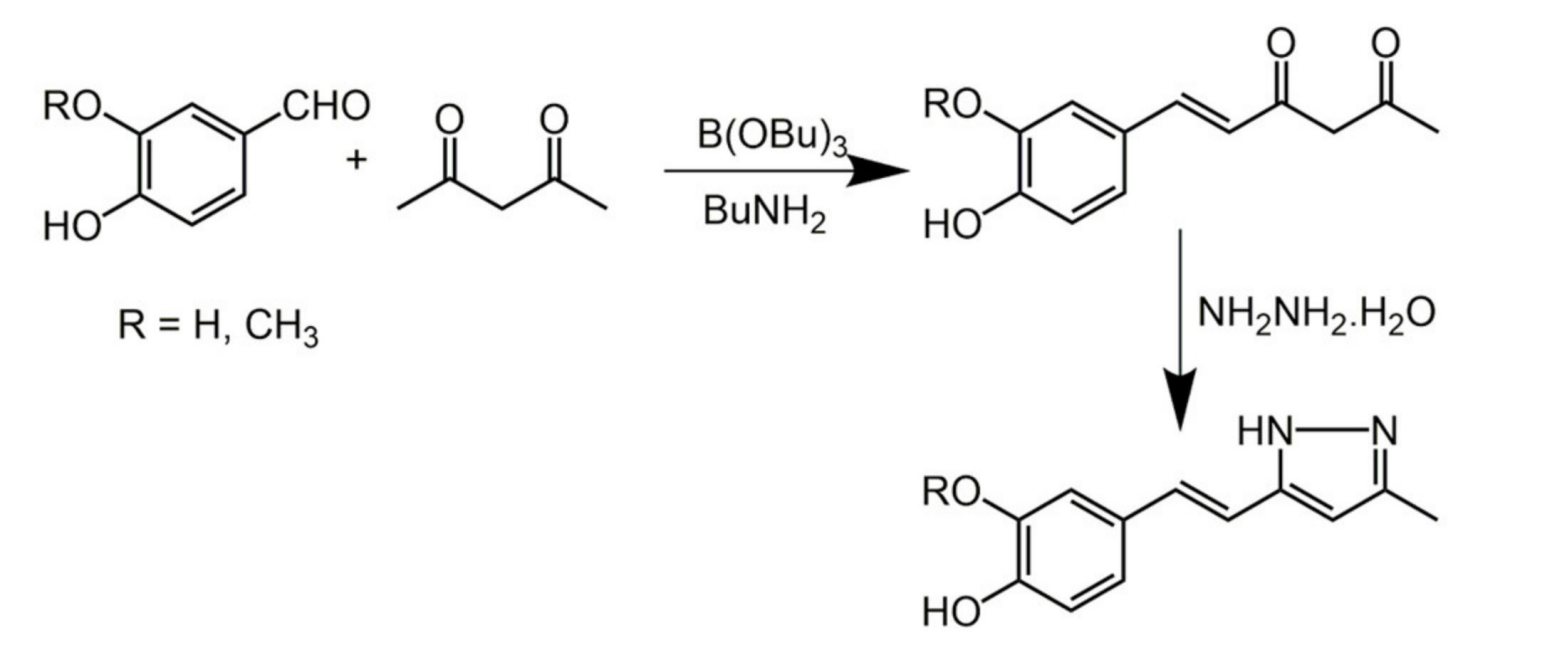
Synthesis of hispolon and hispolon methyl ether pyrazole derivatives [241].

Hispolon (6-(3,4-dihydroxyphenyl)-4-hydroxyhexa-3,5-diene-2-one) is a natural diphenolic compound, extracted from the medicinal mushroom *Phellinus linteus*. It is characterized by peculiar antioxidant activity as well as important pharmacological properties, being a promising anticancer, antidiabetic, antiviral and anti-inflammatory agent [233,234,235]. Recently, several hispolon derivatives have been submitted to in silico predictions to preliminarily evaluate their anticancer activity. Theoretical analysis confirmed that aromatic ring substitution with methoxy and hydroxy groups delivers new hispolon analogues with good antiproliferative activity, sometimes even higher than that of hispolon itself [236,237]. In fact, the hispolon derivatives dehydroxyhispolon methyl ether and hispolon methyl ether exhibited higher in vitro cytotoxicity than hispolon against colorectal cancer [238,239]. The latter was up to 5 times more effective toward colon and prostate cancer cell lines [239]. Dehydrohispolon is a promising antitubercular agent showing lower MIC against *M. tuberculosis* with respect to hispolon [240]. Hispolon and hispolon methyl ether pyrazole derivatives (Scheme 20) showed improved stability with respect to their precursors as well as antigenotoxic effects against radiation exposure [241].

**Scheme 21:**
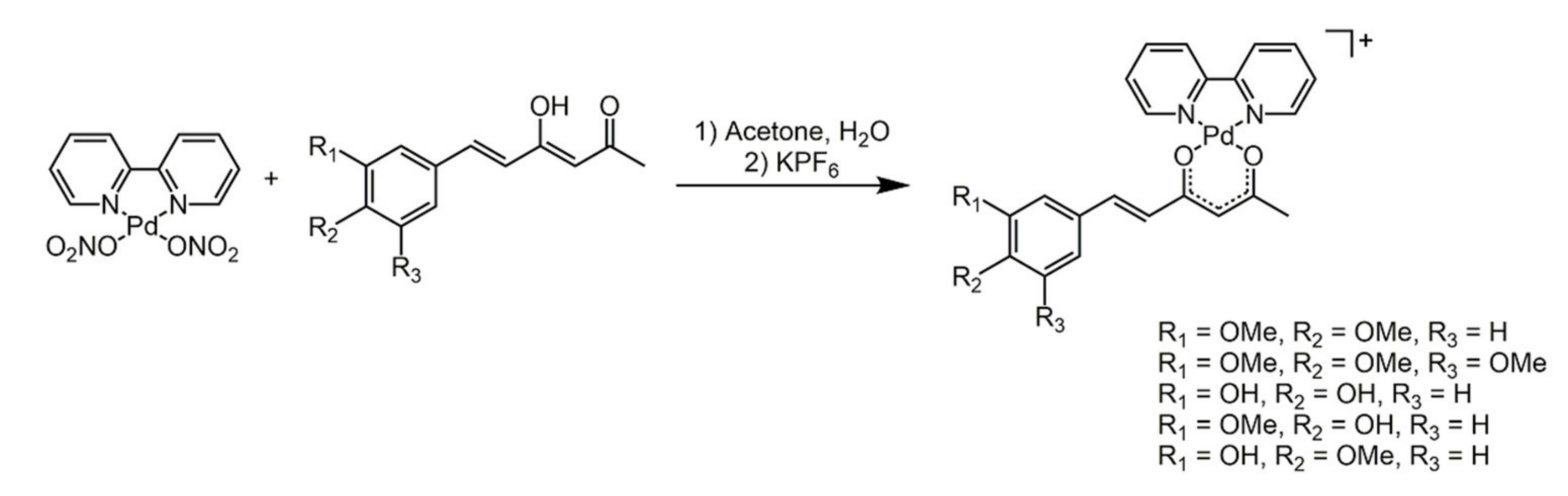
Synthesis of Pd complexes with hispolon derivatives [243].

The pyrazole derivative is also more effective than hispolon for scavenging N_3_• and CCl_3_O_2_• radicals [242]. A family of palladium(II) complexes with hispolon analogues have been recently synthesized and they showed higher in vitro cytotoxicity than the corresponding free ligands against different cancer cell lines. In particular, Pd-complexes with methoxy-substituted hispolon analogues (Scheme 21) showed improved activity compared to the corresponding hydroxyl compounds [243].

### 3.3. Hydroxytyrosol

Hydroxytyrosol (2-(3,4-dihydroxyphenyl)ethanol) is a secondary plant metabolite and it is present in many olive products, being responsible for most of their benefits for human health. The promising pharmacological activities of hydroxytyrosol (as cardioprotective, anticancer, neuroprotective and antimicrobial agent) have been recently reviewed [79,244]. Analogously, hydroxytyrosol acetate (2-(3,4-dihydroxyphenyl)ethylacetate), a diphenolic constituent of olive oil, is characterized by peculiar biological activities [245,246,247,248]. Several hydroxytyrosol derivatives have been explored over the past decades, to extend its biological applications [249,250,251,252]. However, the most common functionalization strategies involve esterification or etherification at the alcoholic -OH group, or substitution on the aromatic ring [253].

**Scheme 22:**
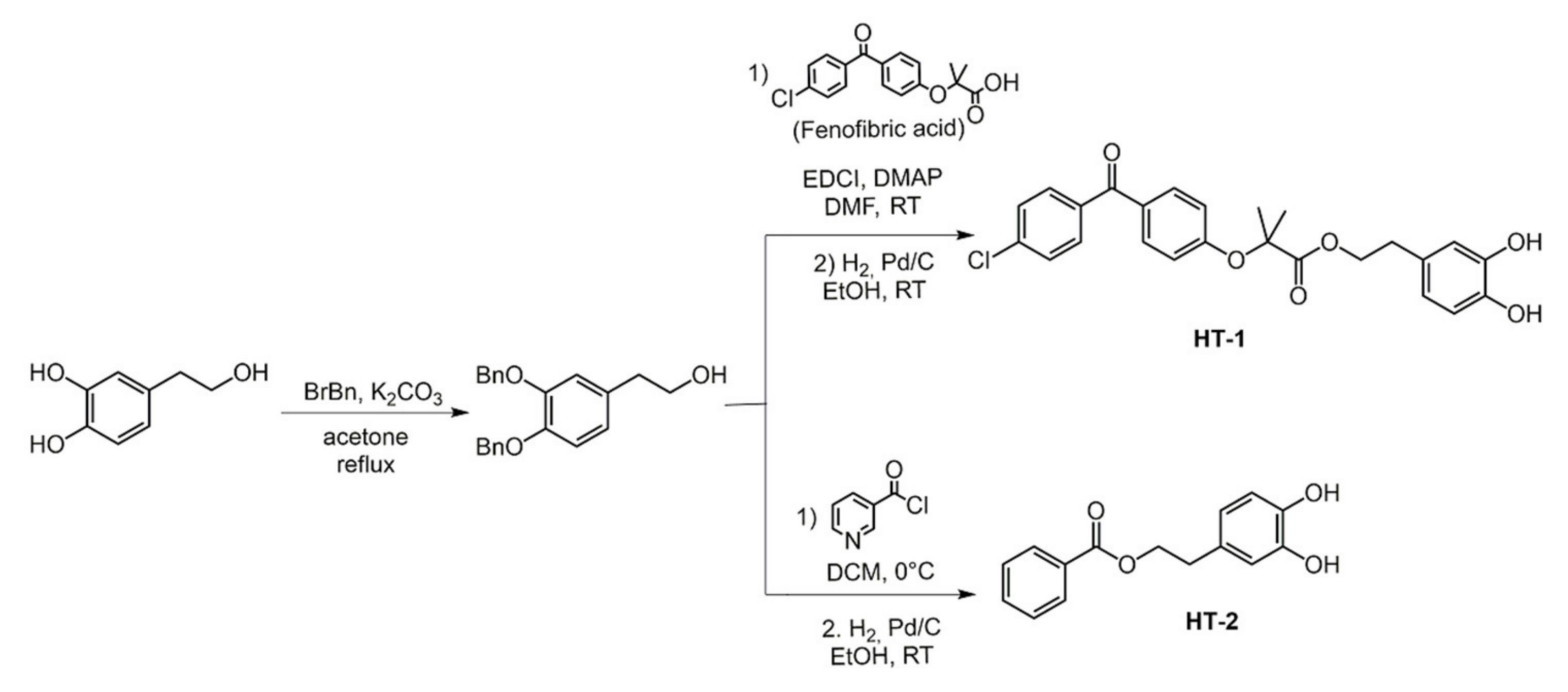
Synthesis of **HT-1** [261] and **HT-2** [262].

As an example, to broaden usage in foods and cosmetics, antioxidant lipophilic hydroxytyrosyl esters have been synthesized through esterification at the alcoholic -OH, with different fatty acids [254,255,256,257], or though the chemoselective transesterification procedure [258]. Interestingly, decanoate and dodecanoate hydroxytyrosyl esters resulted good anti-trypanosomal and anti-leishmanial agents, active against *T. brucei* and *L. donovani*, respectively [259]. Hydroxytyrosol antioxidant activity was enhanced upon esterification to the corresponding butyrate [260], fenofibrate [261] and nicotinate [262] esters (**HT-1** and **HT-2** respectively, Scheme 22), the latter also being a potent α-glucosidase inhibitor.

**Scheme 23:**
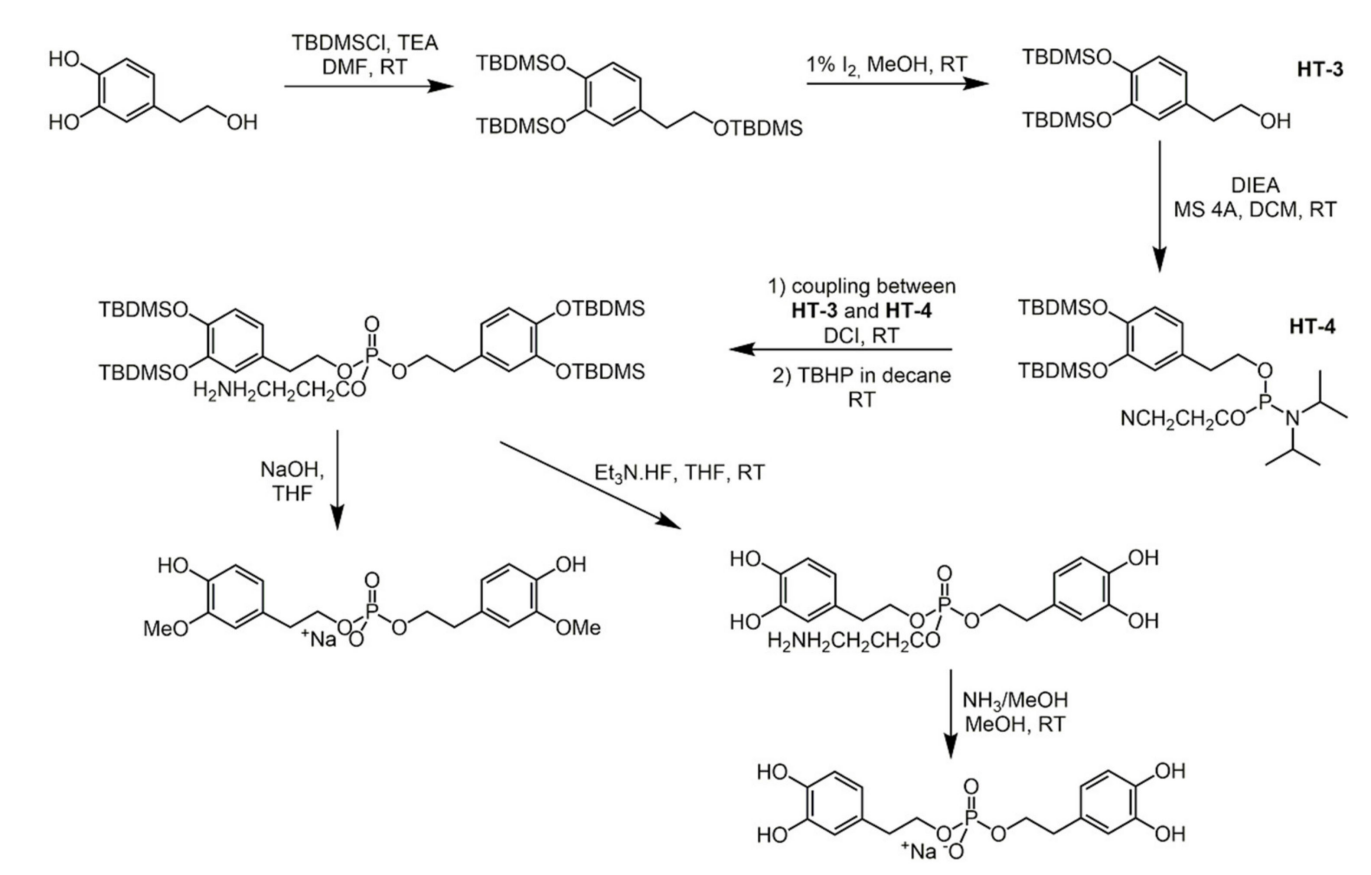
Synthesis hydroxytyrosyl phosphodiesters [264]. Abbreviations: MS = molecular sieves; DCI = 4,5-dicyanoimidazole; TBHP = *tert*-butyl hydroperoxide.

Similarly, a polyacrylate polymer bearing hydroxytyrosol in its side-chain exhibited antioxidant and antimicrobial activity against *Staphylococcus epidermidis* [263]. In addition, hydroxytyrosyl phosphodiesters (Scheme 23) are promising antioxidant agents, suitable for the prevention or therapy of Alzheimer’s disease [264].

Likewise, hydroxytyrosyl alkyl ethers are characterized by interesting biomedical properties. In fact, hydroxytyrosyl hexyl ether showed antiangiogenic [265] and anti-platelet [266] effects, while the ethyl ether exhibited intestinal anti-carcinogenic activity [267], as well as high anti-oxidant properties when added to commercial olive oils [268]. Moreover, the pharmacological potential activity of hydroxytyrosyl glycosides has been recently explored; hence, neuroprotective hydroxytyrosyl glycosides have been synthesized through a sustainable enzymatic reaction, using a fungal *β*-xylosidase as the catalyst to perform the regioselective trans-xylosylation reaction with xylobiose [269].

Nitrohydroxytyrosol, synthesized by the reaction of hydroxytyrosol with sodium nitrite in acetate buffer (pH 3.8), and nitrohydroxytyrosyl ester derivatives (namely acetate, butyrate, hexanoate, octanoate, decanoate, laurate, myristate and palmitate) have been successfully obtained in good yields [270]. NO_2_-hydroxytyrosol showed improved antioxidant activity with respect to the parent compound, while the activity of the ester derivatives was highly dependent on acyl chain length, but good effects were detected with acetate and butyrate. Similarly, the antioxidant activity of alkyl nitrohydroxytyrosyl ethers was maintained or even improved for ethyl and butyl ethers, while, with longer side chains, it was reduced with respect to hydroxytyrosol [271].

**Scheme 24:**
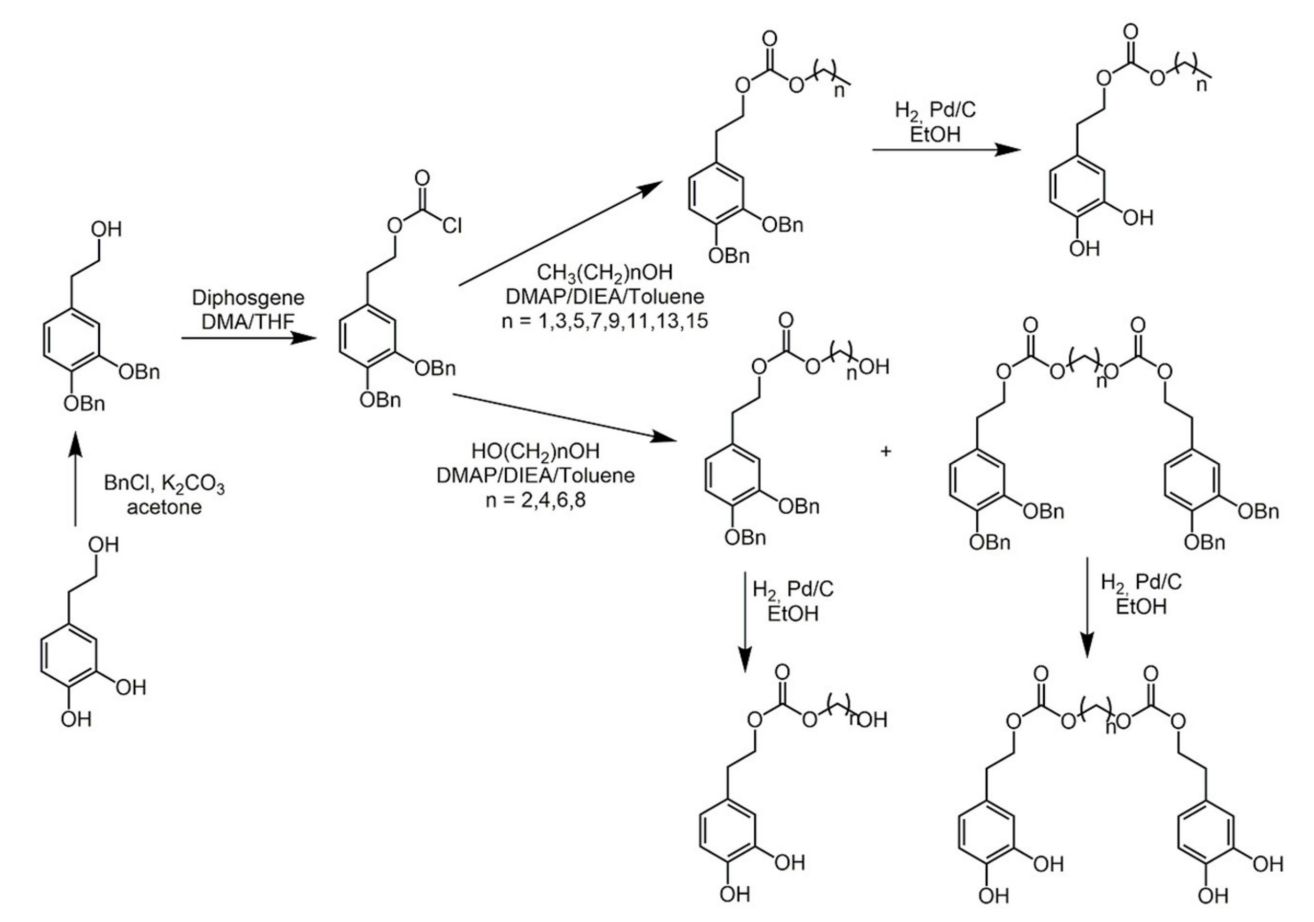
Synthesis of hydroxytyrosol carbonates [272,273].

The synthesis of alkyl carbonate derivatives of hydroxytyrosol is strategic to increase hydroxytyrosol antioxidant activity [272]. Carbonate derivatives have been achieved by a multi-step process in which the chloroformate hydrxytyrosol derivative (having protected phenolic groups) was reacted with alkyl alcohols or diols of different chain lengths [272,273] (Scheme 24). Hydroxytyrosyl carbonates also exhibited higher anti-trypanosome activity against *Trypanosoma brucei* with respect to the precursor [273].

## 4. Phenolic Acids

**Figure 6:**
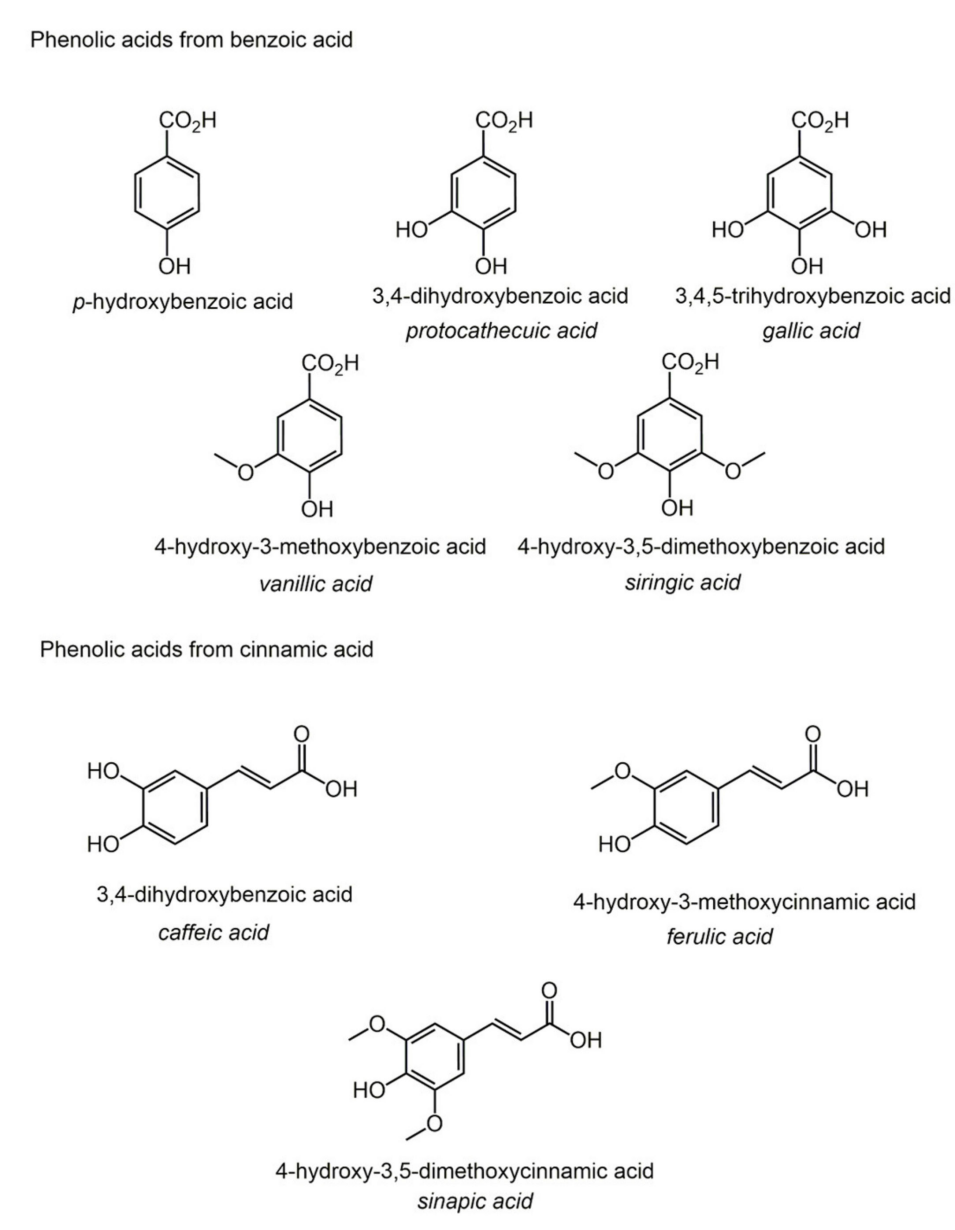
Bioactive phenolic acids widespread in plants.

Phenolic acids are hydroxy or methoxy derivatives of benzoic acid or of cinnamic acid (3-phenylpropenoic acid). They are diffused in many plants (for example, they are among the most abundant natural antioxidants of virgin olive oil [274]). The more frequent phenolic acids are summarized in Figure 6.

Occurrence, biological and pharmacological functions of monocyclic phenolic acids were reviewed, evidencing their wide distribution and variety of functions [275,276,277], together with their promising therapeutic applications [278,279,280,281,282]. As an example, development of gallic acid derivatives as pharmacological agents [283,284] was recently discussed. Similarly, features and potential application of ferulic acid derivatives [285,286,287,288,289] and natural and synthetic products derived from caffeic acid [290] were reviewed.

Therefore, the appealing properties of phenolic acid derivatives are unambiguously affirmed.

Nevertheless, in the following, the most interesting derivatives of natural phenolic acids are discussed.

### 4.1. Caffeic Acid

**Scheme 25:**
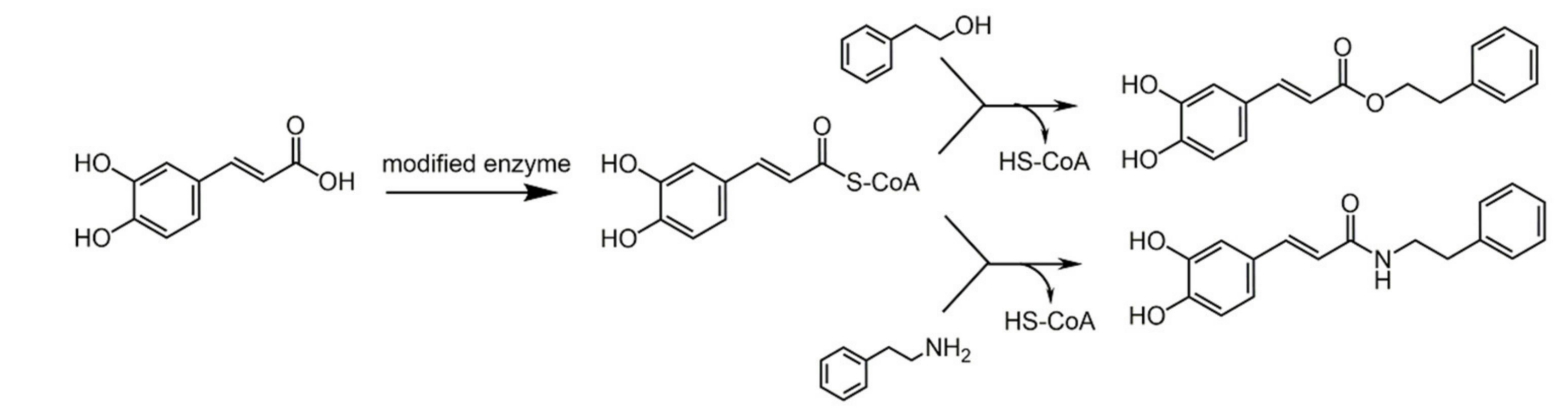
Synthesis of ester and amide derivatives of caffeic acid by artificial biosynthetic pathways [292].

Caffeic acid (3,4-dihydroxybenzoic acid) can be found in coffee, wine and tea. It is characterized by antioxidant, anti-inflammatory and anticarcinogenic activity. Esters of phenolic acids have been of significant interest, and the most interesting biological properties and synthetic methods of caffeic acid phenethyl ester and derivatives have been reviewed [291]. Indeed, caffeic acid phenethyl esters are among the small molecules with anti-inflammatory activity for the treatment of acute lung injury [292]. Non-natural biosynthetic pathways were tailored incorporating caffeic acid into various aromatic alcohol or amine. The new platform permitted the bacterial production of a library of caffeic acid derived phenethyl esters or amides in *Escherichia coli* [293] (Scheme 25).

Caffeic acid enzymatic esterification with phenethyl alcohol in the presence of Novozym 435 in isooctane at 70 °C has been exploited [294]. The enzyme maintained more than 90% of its original activity up to the third run. It is worth signaling that the enzymatic synthesis of caffeic acid phenethyl ester was successfully ultrasound-accelerated [295]. Similarly, the transesterification of methyl caffeate to caffeic acid phenethyl ester analogues was performed with *Candida antarctica* lipase B, using the ionic liquid 1-butyl-3-methylimidazolium bis(trifluoromethylsulfonyl)imide as the solvent [296]. 2-Cyclohexylethyl caffeate and 3-cyclohexylpropyl caffeate exhibited strong antiproliferative activities. Alkyl esters of caffeic acid with 2–8 C atoms were obtained from caffeic acid and the proper alcohol in the presence of DCC [297]. A screening for their antifungal activity was carried out against *Candida albicans*, with the best antifungal performances shown by propyl caffeate. Other alkyl esters of caffeic acid (alkyl = methyl, ethyl, butyl, octyl, benzyl and phenethyl) were prepared refluxing caffeic acid with alkanol in acetyl chloride [298]. In vitro and in vivo experiments confirmed the anti-inflammatory efficiency of the esters.

Twenty-one caffeic acid phenethyl ester derivatives were synthesized, characterized and investigated for their cytoprotective effects [299]. Some of them showed stronger cytoprotective activities than the parent phenethyl ester, so the authors suggested a potentiality as functional food ingredients for the prevention of neurodegenerative diseases.

Caffeic acid and its phenylpropyl ester were investigated for their ability to suppress the proliferation of human colorectal cancer cells both in vitro and in vivo [300], resulting in potent anti-cancer agents.

Twenty ester derivatives of caffeic acid were synthesized to investigate the inhibitory activities against the nitric oxide production induced by lipopolysaccharides [301]. All of them showed inhibitory activity.

**Scheme 26:**
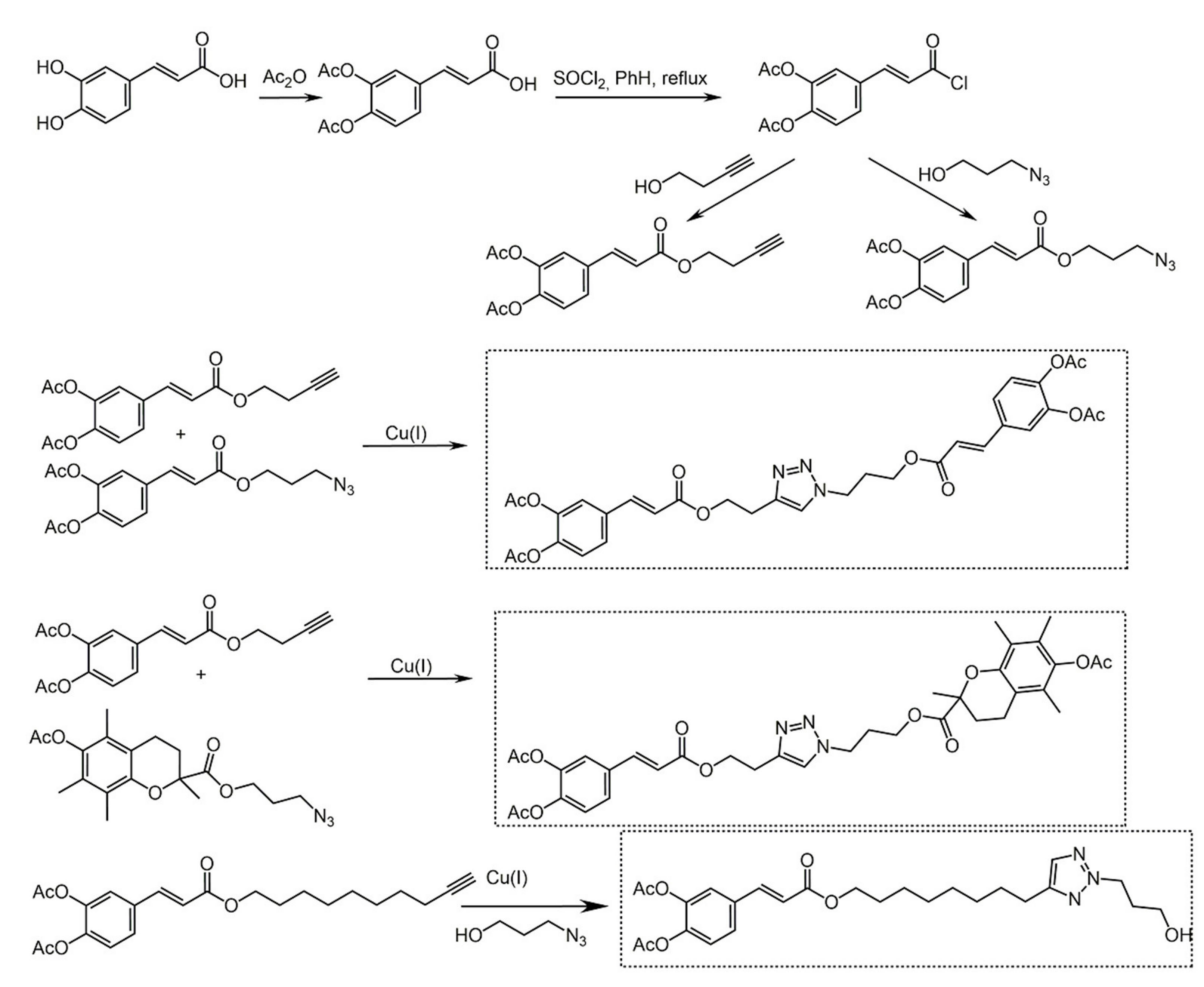
Derivatives of caffeic acid with a triazole moiety inhibitors of 5-lipoxygenase [302].

Semi-synthetic esters derivatives of caffeic acid with a triazole moiety have been designed as potential 5-lipoxygenase inhibitors [302]. 5-Lipoxygenase is involved in the biosynthesis of leukotrienes, the well-known inflammatory mediators with implications in different diseases (asthma, allergic rhinitis, cardiovascular diseases, and certain types of cancer). Structure-guided drug design delivered compounds with excellent 5-lipoxygenase inhibition, especially compounds reported in Scheme 26 that showed enhanced activity compared to caffeic acid.

Amides have been chosen as bioactive derivatives of caffeic acid: several alkyl and aryl amines were used to prepare antioxidant caffeic acid derivatives [303]. Anilides of caffeic acids were found to be efficient as lipid peroxidation inhibitors.

**Figure 7:**
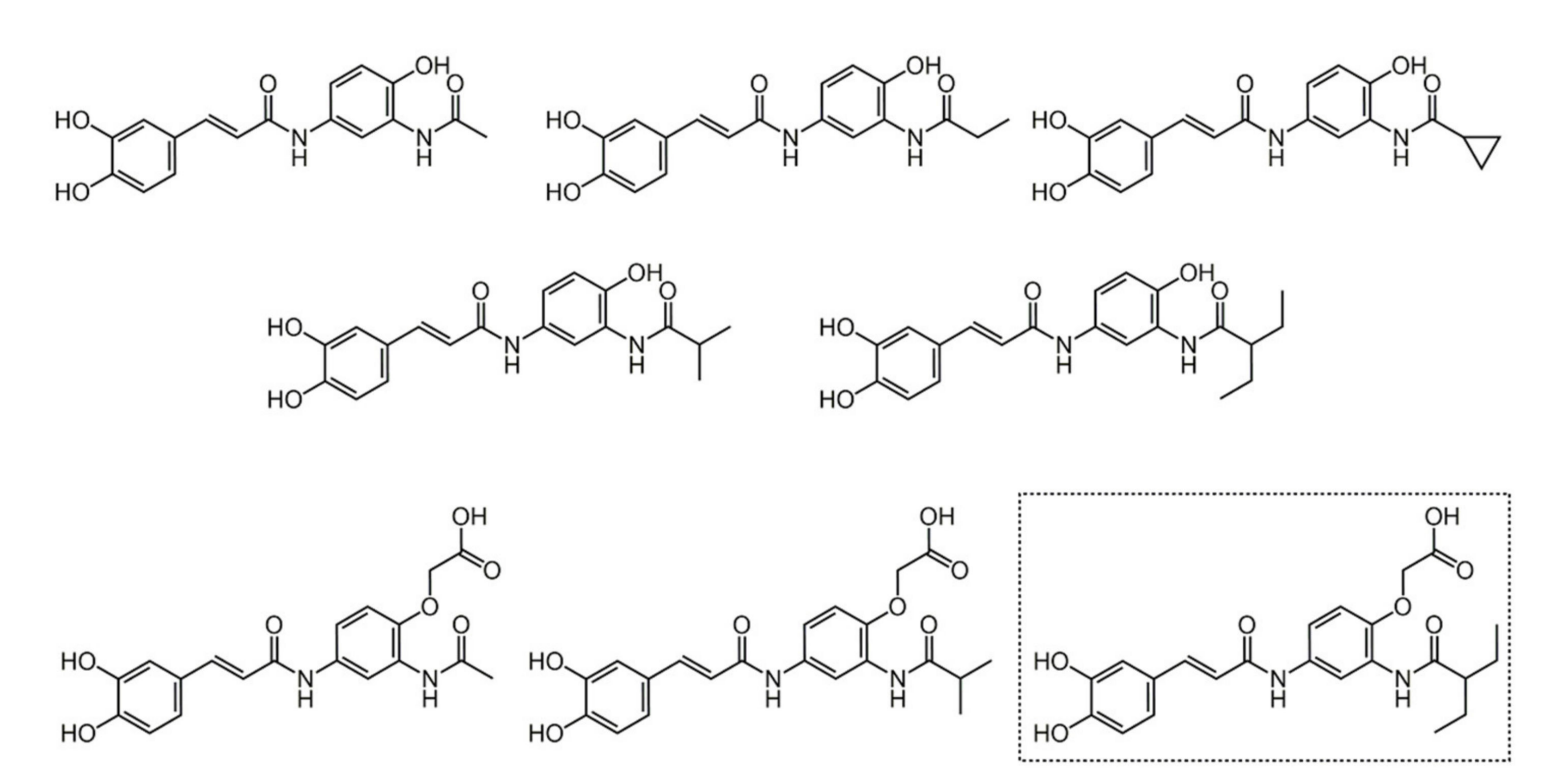
Amides of caffeic acid with antiviral effect against influenza virus [304].

Since some plant-derived polyphenols were found to exert antiviral effects against influenza virus, inhibiting the viral surface protein neuraminidase, a library of caffeic acid-based compounds was built, forming variously substituted amides [304]. The amides presented a moderate activity on neuraminidases and the compound evidenced in Figure 7 is the most promising one.

**Scheme 27:**
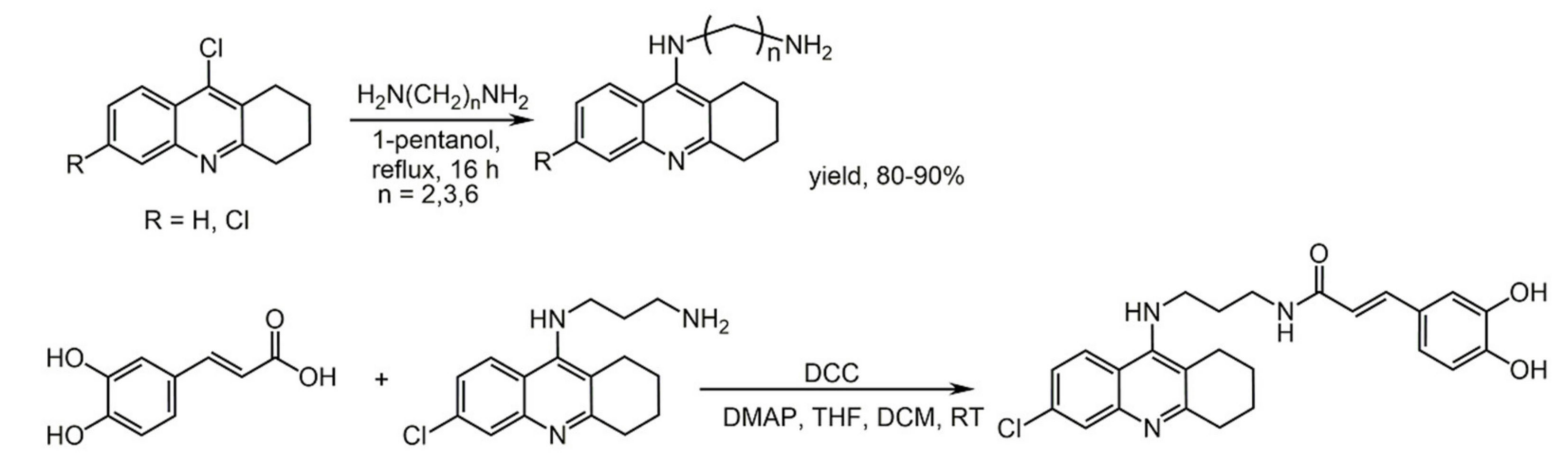
Synthesis of caffeic acid–tacrine hybrids [305].

Amides were prepared from caffeic acid and the heteroaromatic amine tacrine with alkyl spacers of different length (Scheme 27) [305]. All the tacrine derivatives have much more antioxidant efficiency than caffeic acid. In particular, the compound with a 3 C linker and a Cl atom in the aromatic ring was the more selective one, since it had potent neuroprotective effect, inhibiting *β*-amyloid aggregation. These properties make the amide a good candidate for development of beneficial therapeutic tools in Alzheimer’s disease treatment.

A number of acylhydrazides of caffeic acid were prepared and investigated for anti-inflammatory, analgesic and ulcerogenic activities [306]. Caffeic acid was *tert*-butylated in position 5 of the aromatic ring [307]. Both caffeic and *t*-butylated caffeic acids showed strong antioxidant capacity during squalene peroxidation under direct UVA irradiation, but only the latter could reduce the generation of reactive oxygen species.

### 4.2. Ferulic Acid

**Scheme 28:**
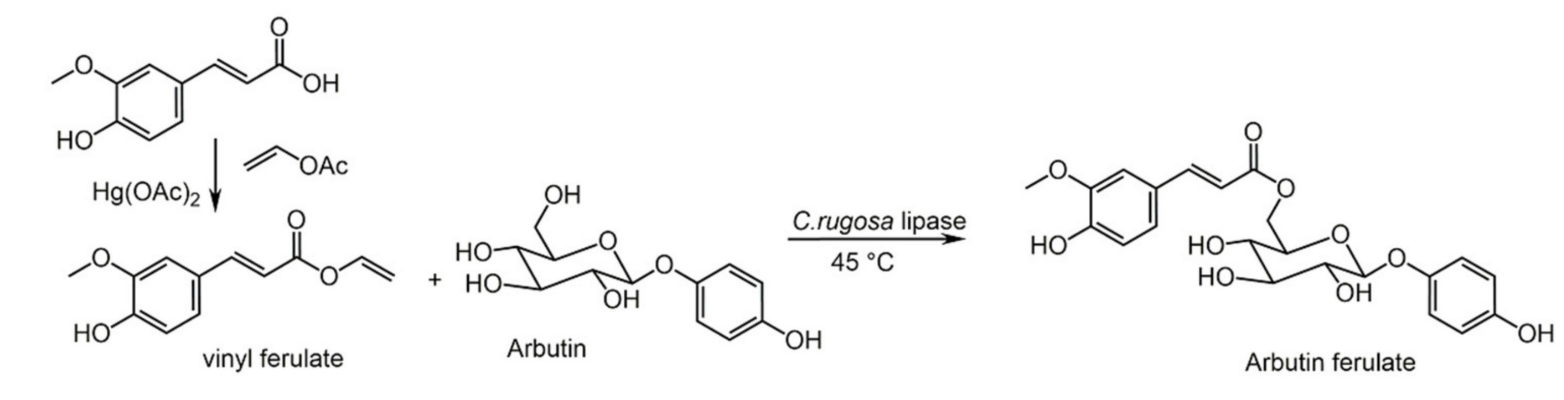
Enzymatic transterification with Arbutin [308].

Ferulic acid (4-hydroxy-3-methoxycinnamic acid) is an effective antioxidant compound, typically found in seeds, leaves, as well as in plant cell wall. Several ferulic acid derivatives have been recently explored in different areas. Lipases from *Candida antarctica* (Novozyme 435), *Candida rugosa*, *Chromobacterium viscosum* and *Pseudomonas sp.* were used to catalyze transesterifications of vinyl ferulate with hydroxyl steroids and arbutin [308] (Scheme 28). Arbutin ferulate possesses a 19% higher antiradical activity than ferulic acid.

**Scheme 29:**
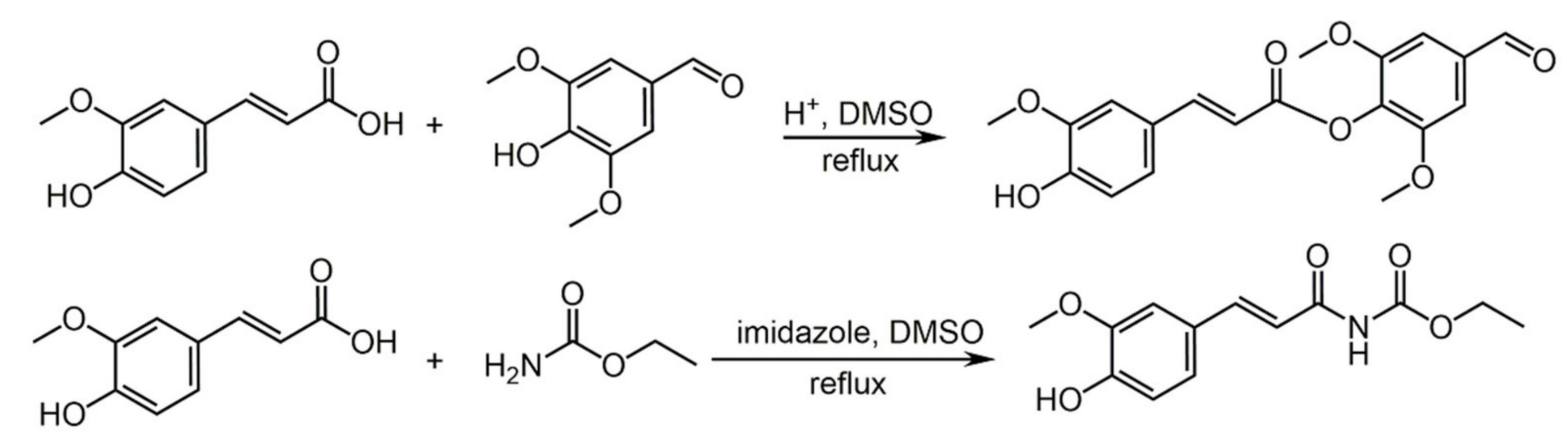
Esters and imides from ferulic acid [311].

Other enzymatic esterification reactions allowed the synthesis of ethyl ferulate from ferulic acid and ethanol, using Novozym 435 as the biocatalyst, that could be reused eight times before significant loss of activity [309]. Lipase-catalyzed preparation of mono- and diesters of ferulic acid was reported [310]. In fact, the exploitation of antioxidant capacity of ferulic acid is limited by its scarce solubility in hydrophobic media such as fats and oils. A strategy to maximize the therapeutic benefits is to transform the acid in esters [311] (Scheme 29). Once prepared, esters were evaluated for in vitro antioxidant potential. Moreover, molecular docking indicated that ferulic esters inhibit target proteins in breast cancer and in oxidative stress.

Alkyl esters and amides of ferulic acid were prepared and evaluated for their anti-cancer activity [312]. According to the authors, all the synthesized amides showed good cytotoxic activity, a characteristic attributed to lipophilicity. Several amides of substituted ferulic acids were also prepared from *O*-acetyl feruloyl chloride with amines [313]. Most of the amides exhibited significant promotion of insulin release from rat pancreatic cells.

**Scheme 30:**
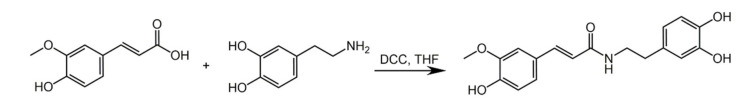
Example of amide from ferulic acid with antioxidant property [314].

A number of amides were prepared from ferulic acid and other phenolic acids (Scheme 30), with the aim to evaluate their antioxidant activity [314]. The results of four different in vitro tests indicated a very high antioxidant activity, especially when –OH or –OMe groups were present in the amide.

**Figure 8:**
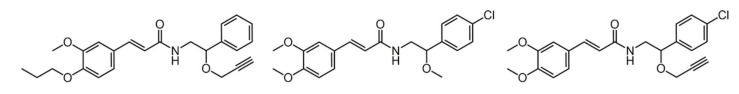
Amides of ferulic acid with in vivo insecticidal activities and antiviral activities [315].

Several ferulic acid amides and amides of the corresponding 3-(4-ethoxy-3-hydroxy)phenylpropanoic acid were synthesized and tested for antiviral and insecticidal activity, against the tobacco mosaic virus [315]. Most of the examined amides presented not only good antiviral activity, inhibiting the plant viral infection, but also insecticidal efficiency against the insect vectors, thus helping in prevention of virus spreading in the crops (Figure 8).

**Scheme 31:**
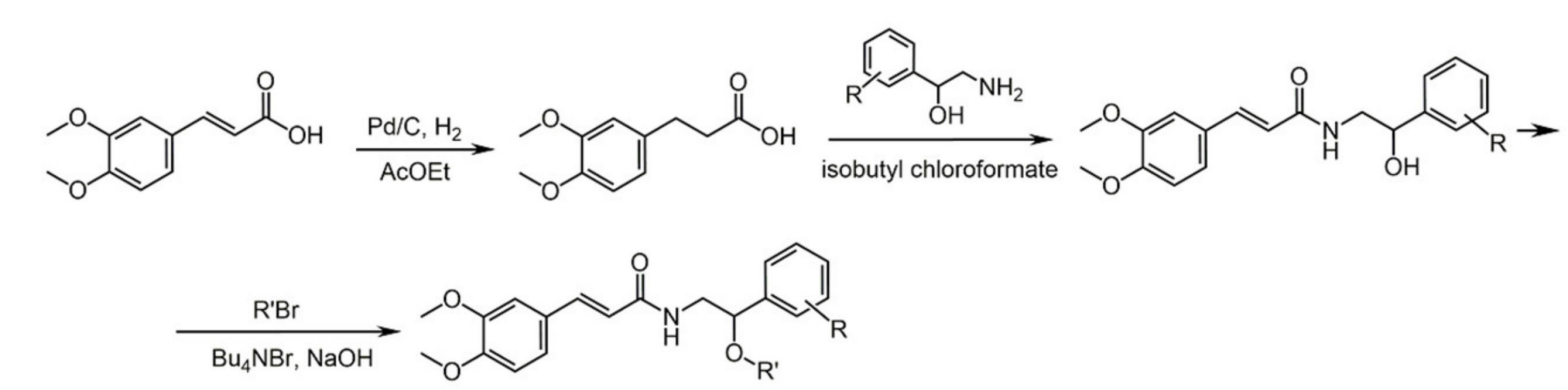
Synthesis of biologically active amides of 3-(3,4-dimethoxy)phenylpropanoic acid [316].

Amides of hydrogenated ferulic acid, 3-(4-hydroxy-3-ethoxy)phenylpropanoic acid, were synthesized (Scheme 31) [316]. Some of them displayed excellent protection and curative activity against the tobacco mosaic virus.

The feruloyl and phenethyl groups were combined to synthesize the ester phenethyl *trans*-3-(4-hydroxy-3-methoxyphenyl)acrylate and the amide *trans*-3-(4-hydroxy-3-methoxyphenyl)-*N*-phenethylacrylamide [317]. Phenethyl ester and amides were studied for bioactivity as anticancer agents, proving more effective than the parent compound [318].

Many other amides of ferulic acid were prepared under solvent-free conditions by MW assisted condensation reaction [319]. Twenty-one amides showed noticeable in vitro anticancer activity, nine presented in vitro free radical scavenging activity higher than that of ferulic acid.

Hexyl caffeate and ferulate as well as caffeoylhexylamide and feruloylhexylamide were tested in human breast cancer cell lines [320]. Differently from parent compounds, the new compounds inhibited cell proliferation and they induced cell cycle alterations and cell death.

**Scheme 32:**
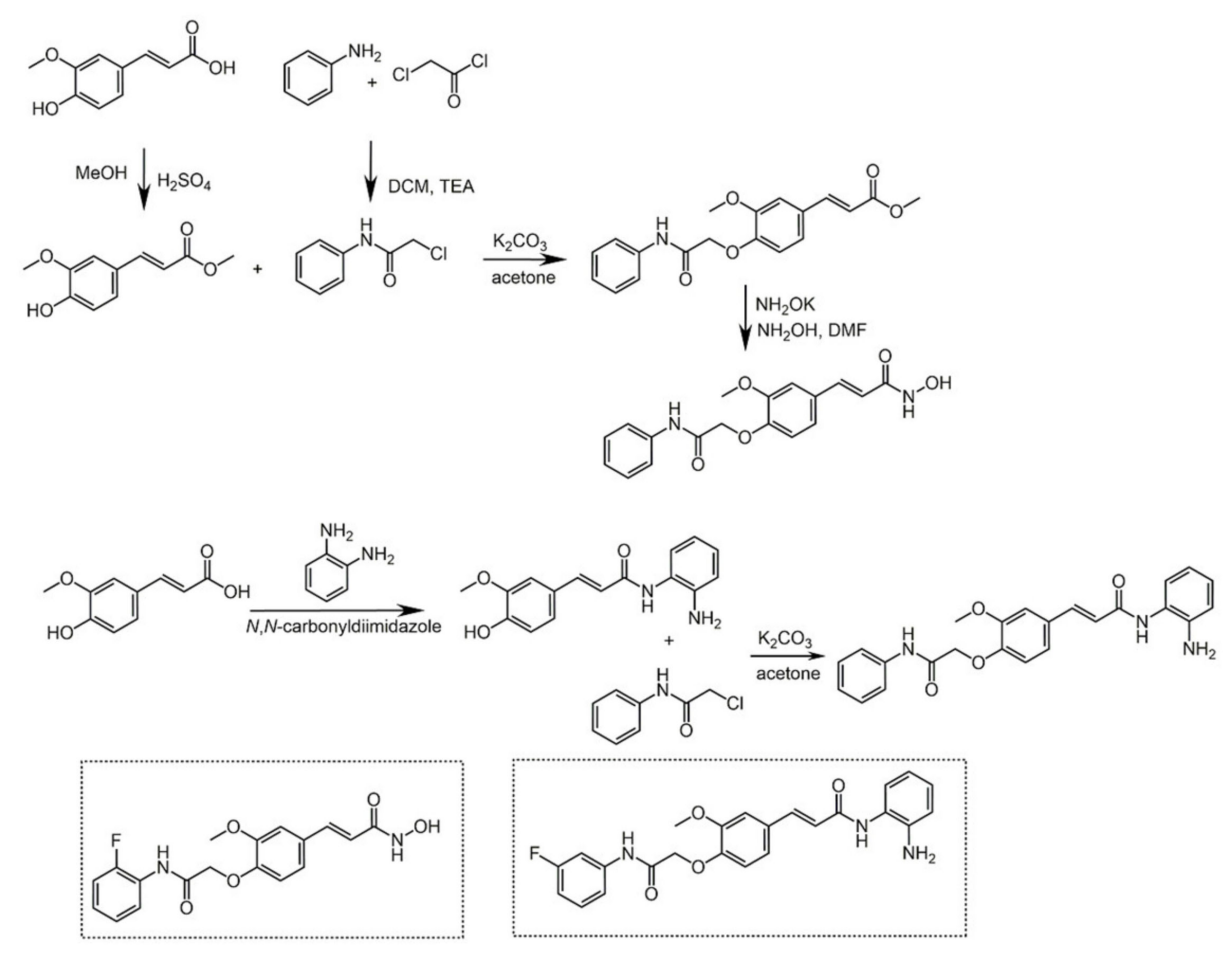
Synthesis of ferulic acid amides designed to be used as histone deacetylase inhibitors [321].

Twenty seven ferulic acid derivatives, with halogeno-acetanilide as novel surface recognition moiety, were prepared with the aim to obtain compounds able to act as histone deacetylase inhibitors. The latter class of compounds was important for cancer therapy [321]. Synthesized compounds evidenced at the bottom of Scheme 32 exhibited significant enzymatic inhibitory activities.

Notably, the geranylated derivative of ferulic acid, 3-(4*O*-geranyloxy-3-methoxyphenyl)-2-propenoate resulted more active than the parent ferulic acid as inhibitory agent on aberrant crypt foci, reducing the incidence of adenocarcinomas in the colon [322].

### 4.3. Miscellanea

Methyl, ethyl, propyl and butyl esters of sinapic acid (4-hydroxy-3,5-dimethoxycinnamic acid, a bioactive natural compound found in fruits, vegetables and medicinal plants), prepared by acid-catalyzed esterification of alcohols, presented almost the same antioxidant activity, which is slightly lower than that of sinapic acid [323]. However, they have the advantage of higher lipophilicity, which is valuable for antioxidants designed to act as membrane protectors in vivo.

Alkyl esters of protocathecuic and gallic acids (3,4-dihydroxybenzoic acid and 3,4,5-trihydroxybenzoic acid, respectively), prepared reacting the acid with different alkanols in presence of DCC, were found to inhibit HIV-1 protease dimerization [324], with efficiency depending on the length of the alkyl chain. In fact, no inhibition was observed with alkyl chains with less than eight carbon atoms. Protocatechuic acid and its ethyl and heptyl esters were investigated for the photoprotective activity [325]. The photoprotection and antiaging activity was higher with the esters than with the parent acid, probably because of their higher lipophilicity. Unfortunately, cytotoxicity also increased.

Alkyl esters of gallic acid were prepared by the classical Fisher esterification, from acid and alcohols in acid medium [326]. Besides anti-cancer, antiviral and antimicrobial properties, they are able to scavenge and reduce reactive oxygen species formation. Additionally, gallic acids esters are potential inhibitors of metastasis [327]. The propyl ester of gallic acid was transformed into the acyl hydrazide that, upon reaction with the appropriate calchone, was transformed into pyrazole derivatives of gallic acid [328]. The compounds were screened for their anti-inflammatory activity, with good results in some cases.

New syringic hydrazones from the aldehyde related to syringic acid (4-hydroxy-3,5-dimethoxybenzoic acid) and substituted hydrazines (with electron withdrawing or electron attracting groups) [329] were efficient against the oxidative stress.

## 5. Lipidic Phenols

Lipidic phenols (or phenolic lipids, also called phenolipids) are phenols substituted with lipophilic chains, that confer to the molecule amphiphilic characteristics. An important phenolic lipid is *α*-tocoferol [330]; however, this compound alone deserves a review, so it has not been included.

The importance of natural lipidic phenols has been underestimated for a long time [331]. However, their excellent antioxidant, antigenotoxic and cytostatic properties are now established [332], together with their bioactivity in influencing biological pathways involved in the Alzheimer’s disease pathogenesis [333]. Anti-inflammatory and anti-arthritis activities were also reported for lipidic phenols extracted from cashew nut (*Anacardium occidentale*) [334].

Because of the importance of such class of compounds, several synthetic lipidic phenols have been proposed in recent decades to further extend their biological applications.

### 5.1. Biocatalyzed Syntheses of Lipidic Phenols

Lipases are the enzymes of choice to perform transesterification reactions to obtain modified or synthetic lipids, with functional or pharmaceutical applications [335,336]. A large number of esters from catechol and fatty acids were prepared stirring a suspension of catechol in ethyl ester of a fatty acid [337].

**Scheme 33:**
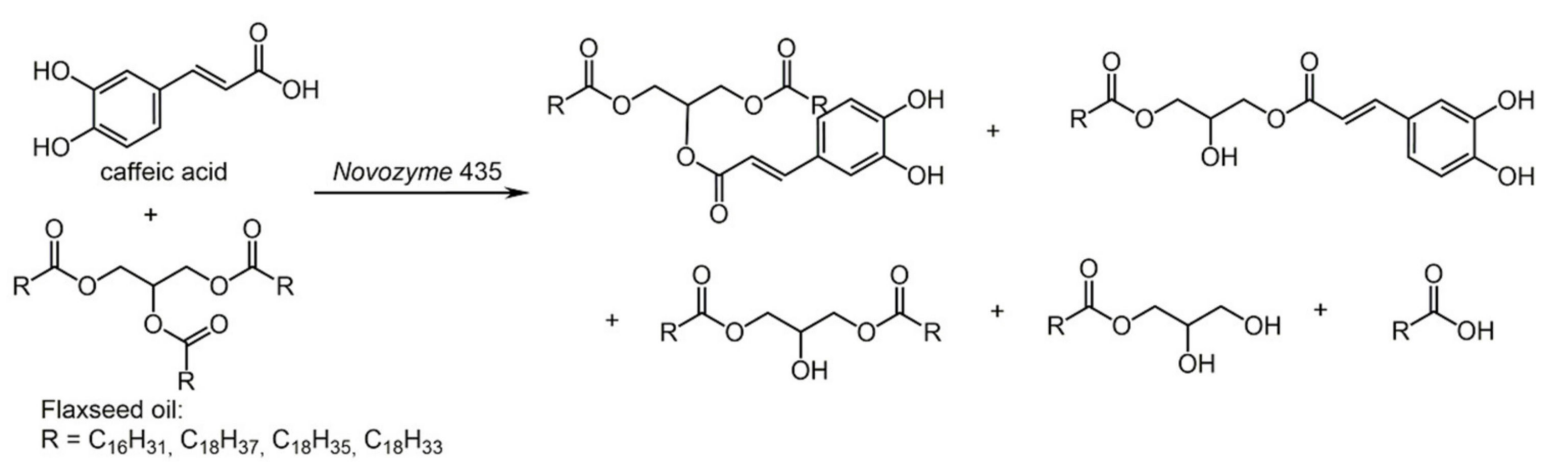
Lipase-catalyzed transesterification of flaxseed oil with caffeic acid [338,339].

Semisynthetic lipidic phenols were prepared by transesterifications reactions of phenolic acids with flaxseed oil [338,339], olein [340], fish liver oil [341] and krill oil [342]. The reactions, performed in organic solvents or in a solvent-free systems [343,344], were catalyzed by Novozym 435 isolated by *Candida antarctica* (Scheme 33).

More recently, yields improved using supercritical carbon dioxide as the reaction medium [345]. The prepared mixtures of lipidic phenols were tested for antioxidant activity. The obtained radical scavenging activity ranged from moderate to good, but it was always lower than that of *α*-tocoferol. Moreover, being obtained from mixtures, the results could not be attributed to a single compound.

**Scheme 34:**
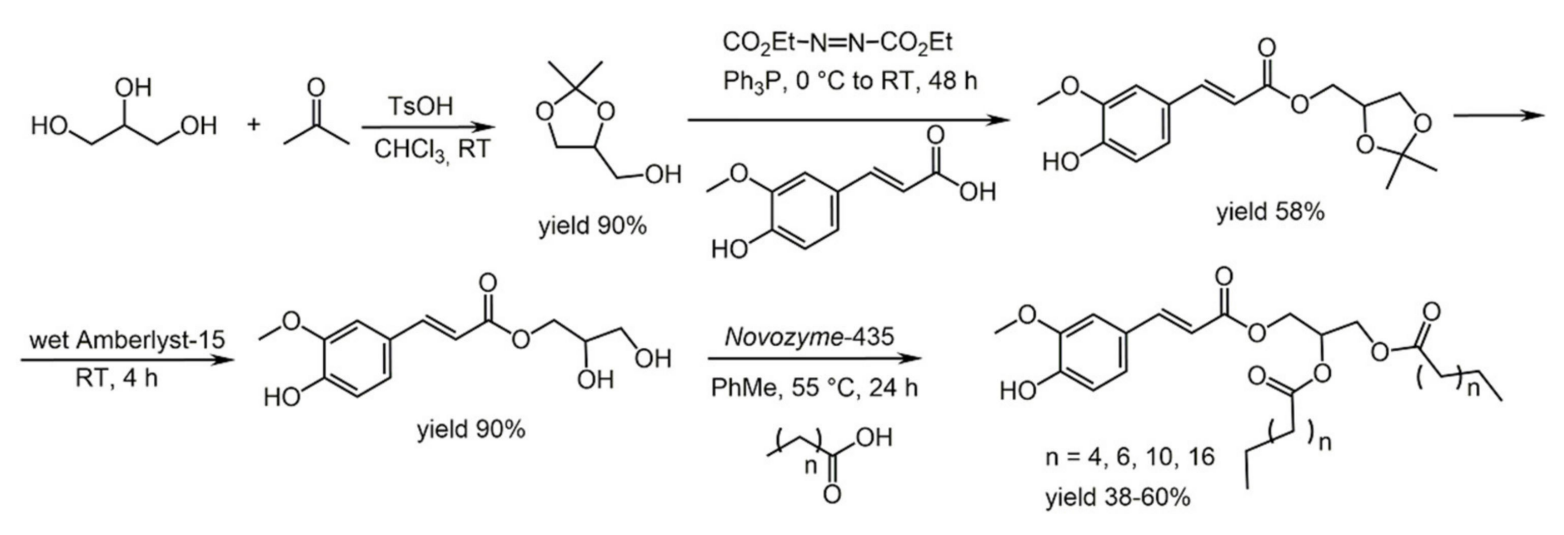
Chemo-enzymatic synthesis of lipidic phenols from ferulic acid [346].

Conversely, pure phenolic lipids of varying chain-length fatty acids were prepared with ferulic acid [346]. The synthesis involved a bio-catalyzed step (by Nosozyme) (Scheme 34).

The antioxidant activity investigation gave mixed results because the radical scavenging assay showed no improvement with respect to ferulic acid, while autoxidation of linoleic acid in a micellar system showed some improvement, attributed to increased solubility.

Chemo-enzymatic synthesis of phosphatidylcholines containing phenolic acid and fatty acids was reported [347], with one of the active derivatives, 1-(4-hydroxy-3,5-dimethoxy)cinnamoyl-2-acyl-*sn*-glycero-3-phosphocholine, showing excellent antioxidant activity.

**Scheme 35:**
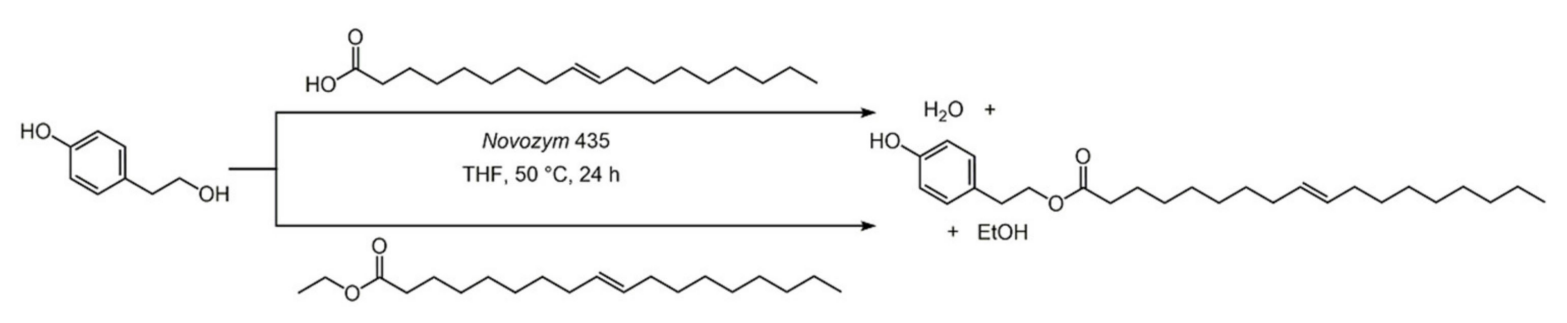
Chemo-enzymatic synthesis of lipidic phenols from ferulic acid [346].

A different approach was to obtain lipidic phenols from a phenol and free fatty acids or the corresponding esters, with immobilized lipase from *Candida antarctica* as the biocatalyst [348]. The antioxidant activity of tyrosol was increased upon acylation, but no correlation was found with the number of double bonds in the fatty acyl group (Scheme 35).

**Scheme 36:**
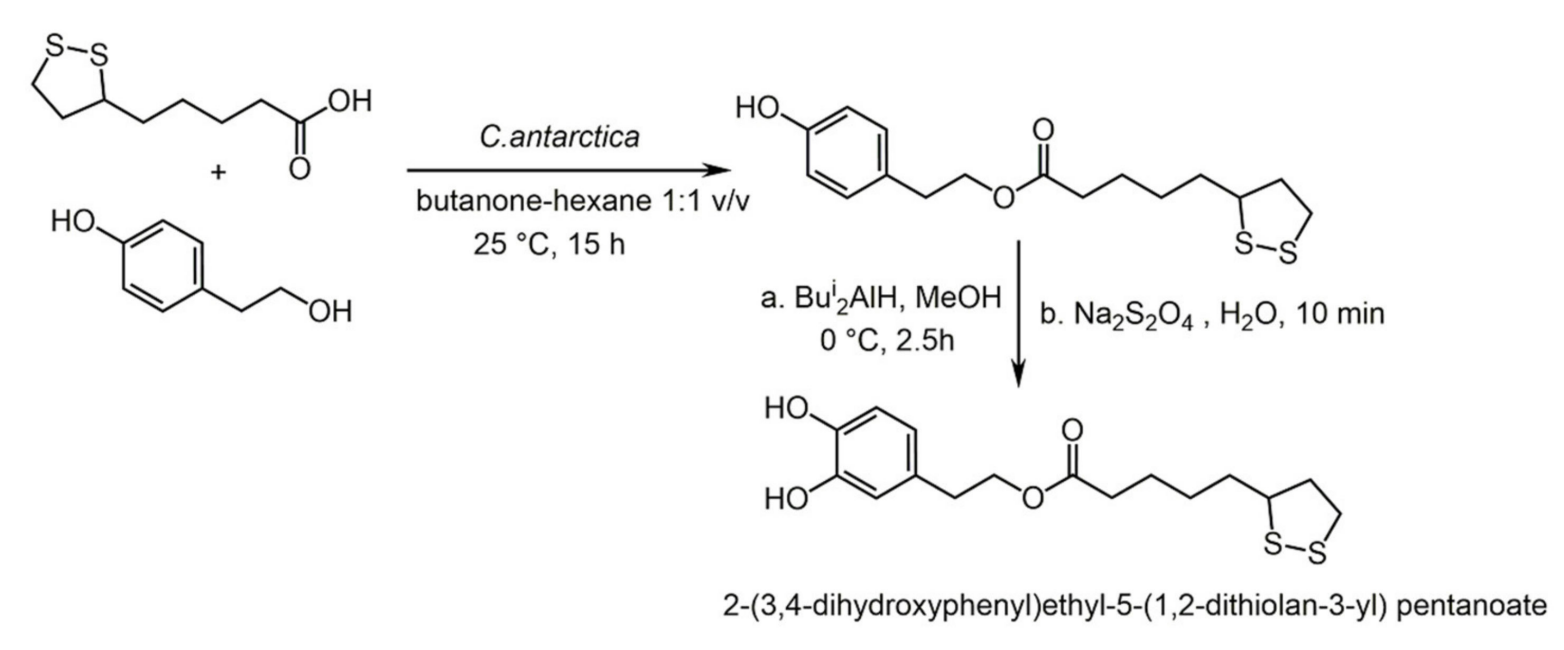
Enzymatic esterification of tyrosol with *α*-lipoic acid [349].

Some esters were synthesized from natural phenols and *α*-lipoic acid in a reaction catalyzed by Novozym 435 (the immobilized lipase B from *Candida antarctica*) in butanone–hexane mixture [349]. The antioxidant activity was determined not only by radical scavenging assay, but also by measuring the inhibition of the oxidation in a tuna fish oil emulsion. The ester 2-(3,4-dihydroxyphenyl)ethyl-5-(1,2-dithiolan-3-yl)pentanoate, obtained from tyrosol and *α*-lipoic acid, followed by aromatic hydroxylation (Scheme 36), showed excellent antioxidant activity in both tests and, according to the authors, it may be used as pro-drug, because, upon hydrolysis, it releases compounds which are non-toxic or even healthy.

Later, it was found that 2-*S*-lipoyl caffeic acid methyl ester was an inhibitor of tyrosinase from human melanoma cells [350].

**Scheme 37:**
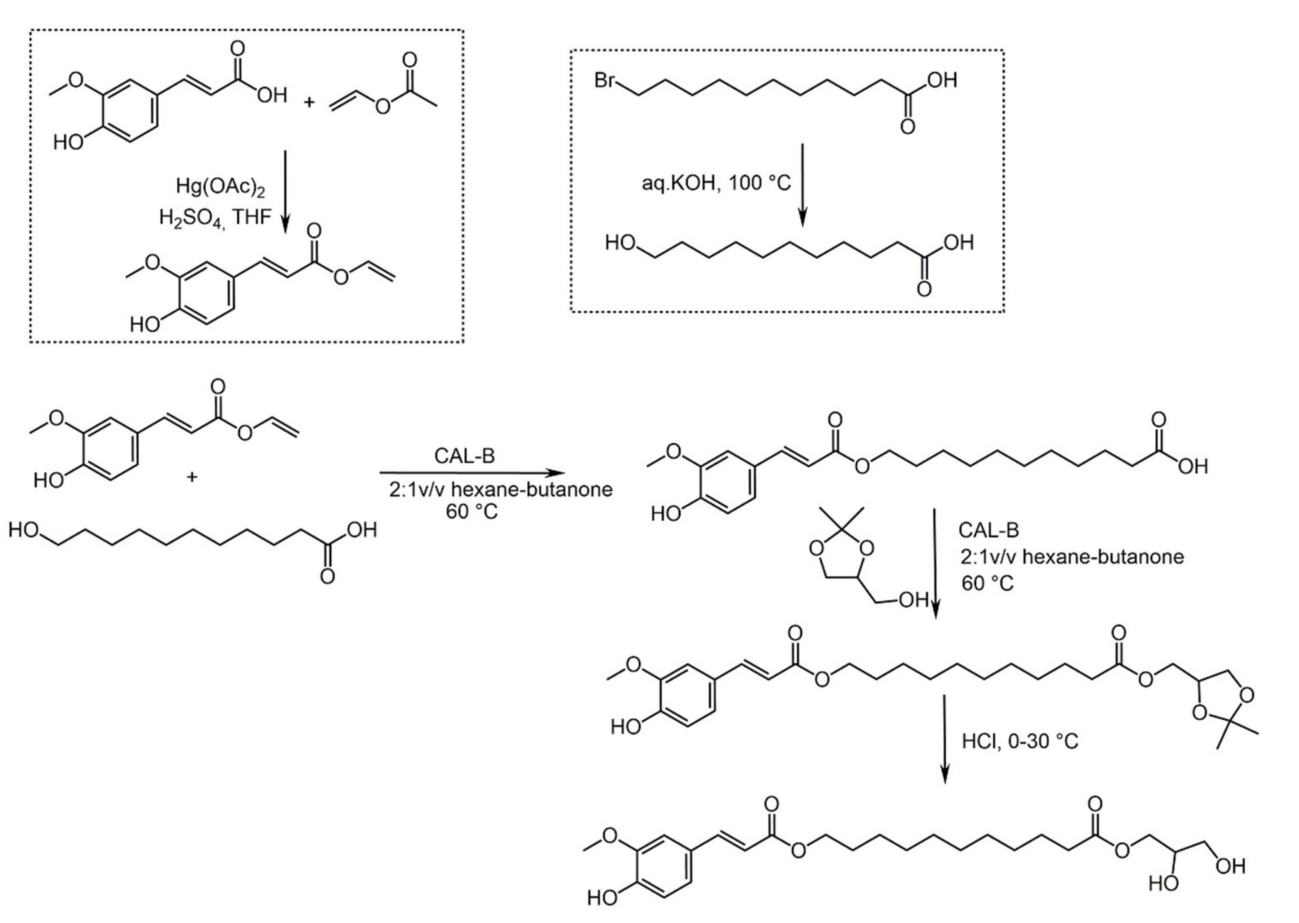
Chemo-enzymatic approach for the synthesis of 1-[11-(ferulyloxy)undecanoyl)]glycerol [351].

The goal to prepare a single lipidic phenol, avoiding troublesome separation of a complicated mixture, was achieved by a multi-step strategy involving both chemical and enzymatic catalysis. Immobilized lipase from *Candida antarctica* (CAL-B) was used in organic medium [351] (Scheme 37).

After characterization, the prepared 1-[11-(ferulyloxy)undecanoyl)]glycerol underwent antimicrobial, antioxidant and cytotoxic studies. The antimicrobial activity was moderate, the antioxidant activity was excellent and activity against some cancer cell lines was promising, so the authors predicted potential cosmetic and biomedical applications.

Transesterification reaction using *Candida antarctica* lipase B was performed, treating 4-hydroxyphenylacetic acid with triolein and with fish oil, obtaining synthetic substances that have both antioxidant and antibacterial activity [352].

### 5.2. Chemical Syntheses of Lipidic Phenols

Lipidic phenols were prepared as esters, either from phenols with long-chain carboxylic acids, or from phenolic acids. Selected examples of synthetic lipidic phenols are reported in Table 1.

**Scheme 38:**
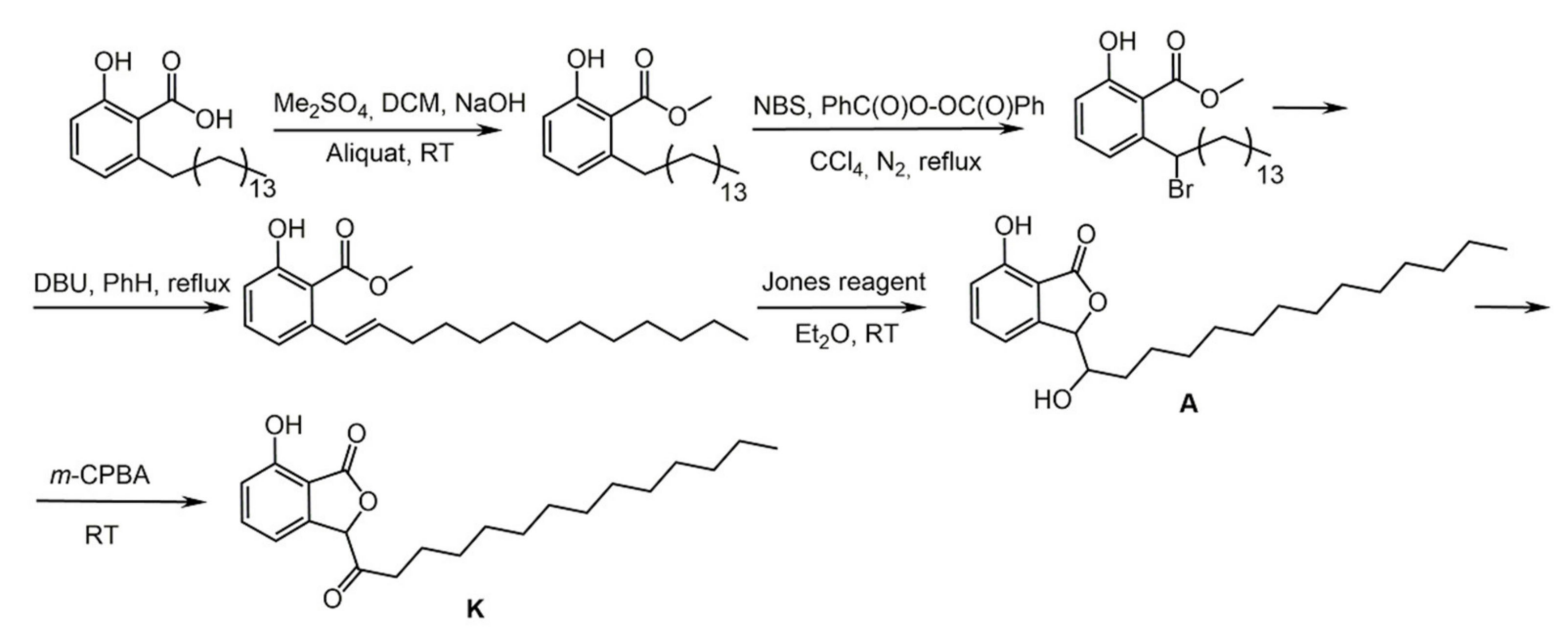
Synthesis of isobenzofuranones from anacardic acid [360]. Abbreviation: *m*-CPBA = *m*-chloroperoxybenzoic acid.

Anacardic acid, from fresh and dry cashew nuts of *Anacardium occidentale*, was transformed into isobenzofuranones, as illustrated in Scheme 38, with an alcohol (A) or keto (K) functionality in the long chain [360].

Both isobenzofuranones A and K, as well as the acyclic precursor, were from moderately to significantly active in cytotoxicity screening with different human cancer cell lines.

A good antioxidant ability to stabilize olive oil was observed with a family of phenolic fatty acid esters, prepared from 3,4-dihydroxybenzyl alcohol (protocatechuic alcohol) or hydroxytyrosol and fatty acids. The reaction was performed in anhydrous THF, in the presence of a carbodiimide and DMAP [361]. All twenty compounds were investigated as potential antioxidants in refined olive oil. The length of the alkyl chain attached to the phenyl ring seems to influence the activity.

**Scheme 39:**
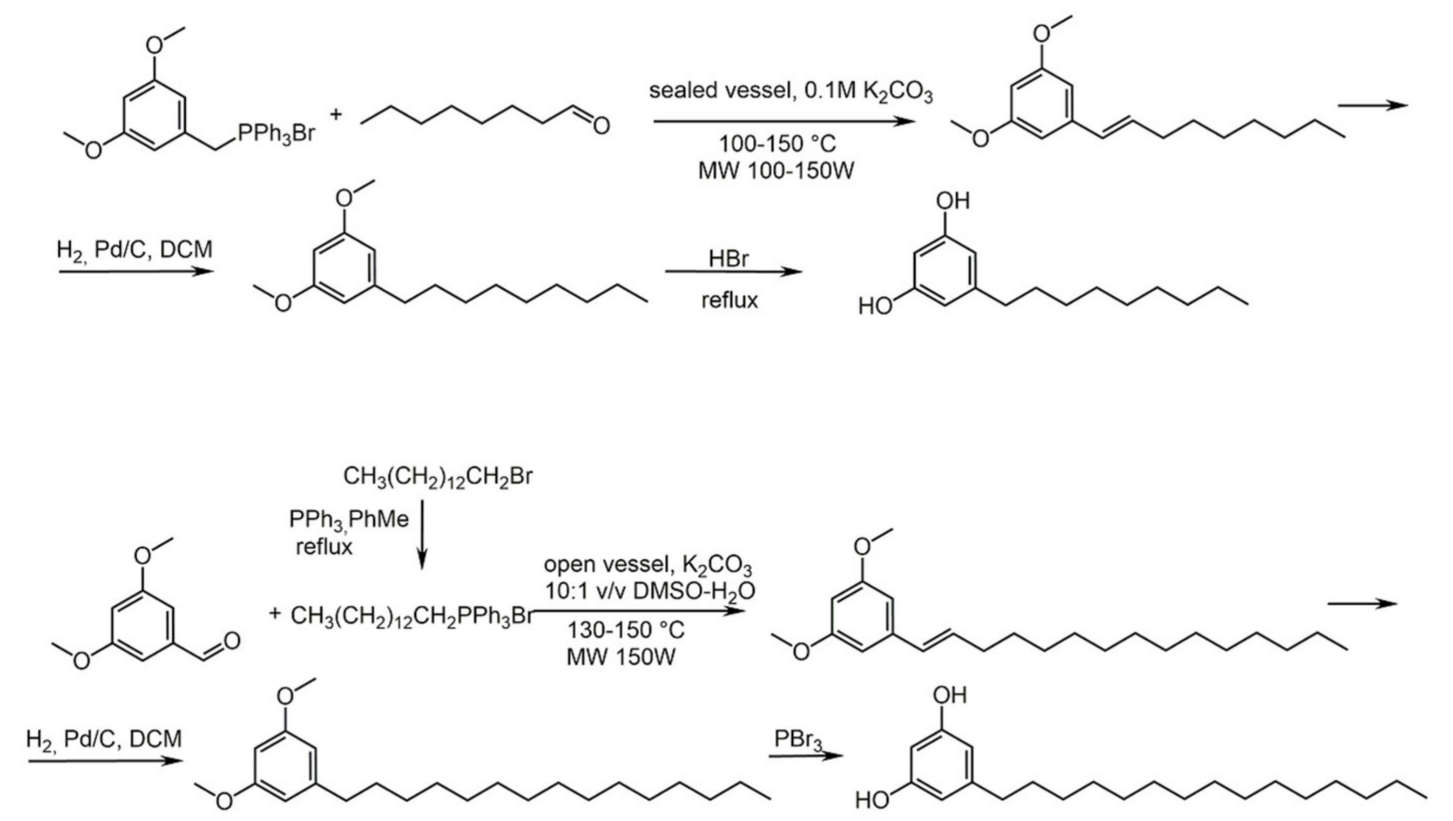
Synthesis of bioactive 5-alkylresorcinols with long alkyl chains, by modified Wittig reaction [363].

Following the finding that 5-alkyl- and 5-alkenylresorcinols, isolated from the mushroom *Merulius incarnates*, inhibited methacillin-resistant *Staphylococcus aureus* [362], a good synthetic method was necessary, because they are not readily available, but are important for analytical, metabolic and bioactivity studies. A general method, based on the Wittig reaction, was developed [363] to overcome the problem of alkyl chain introduction into the aromatic ring. The problem was solved reacting long chain alkanals, when available, with semi-stabilized benzylphosphoniumyilids or, alternatively 3,5-dimethoxybenzenecarbaldehyde with alkylphosphonium ylids. The procedure delivered 5-alkylresorcinols with alkyl chains up to 25 C atoms. The reaction was performed in water or a water–DMSO mixture, with MW irradiation, in pressurized or in open vessel. One example for each route is shown in Scheme 39.

Moderate to good in vitro antioxidant activity was shown by 1,2-dibutanoyloxy-2-(4-hydroxy-3-methoxyphenyl)ethyl butanoate, a lipidic phenol obtained from ferulic acid [354].

2-Methyl-5-[(2*Z*)-non-2-en-1-yl]benzene-1,3-diol and 5-[(2*Z*)-non-2-en-1-yl]benzene-1,2,3-triol were prepared as synthetic derivatives of the natural 5-[(2*Z*)-non-2-en-1-yl]benzene-1,3-diol (climacostol), a defence chemical in protozoan *Climacostomum virens* [355]. The structural modifications in the aromatic ring (a methyl and a hydroxyl group, respectively) increased the toxicity.

Long chain alkyl hydroxycinnamates were prepared from the corresponding monoesters of malonic acid and benzaldehyde derivatives by Knoevenagel condensation [364]. The observed antioxidant activity followed the order caffeic esters > sinapic esters > ferulic esters.

12-Hydroxy-9-octadecanoic acid (ricinoleic acid) was transformed in (*Z*)-methyl-12-aminooctadeca-9-enoate and then reacted with phenolic acids, forming the corresponding amides [356]. The investigated antioxidant activity indicated that modification of phenolic acids with lipophilic moieties improves their antioxidant and anticancer properties.

It is worth signaling a conceptually different type of lipidic phenols, namely the phenylsulfonylfuroxan derivatives of caffeic and other phenolic acids [357]. Besides good antioxidant activities in vivo, these compounds showed anticoagulant and vasodilatation effects that were attributed to the NO releasing ability. 

Caffeic or 3,4-dimethylcaffeic acids were reacted with malic acid and then coupled with monoglicerids of fatty acids with chain lengths ranging from 8 to 18 C atoms [359]. The combinations of phenolic and fatty acids gave a series of six amphiphilic compounds that were tested for activity. They proved to be non-toxic and gave stable oil-in-water emulsions, with potential for food, pharmaceutical and cosmetic industry applications, according to the authors.

## 6. Polyphenols

Natural polyphenols constitute a numerous and largely distributed group of bioactive molecules in edible plants, with bioactivities ranging from cardiovascular protection to prevention of cancer [365,366,367,368].

**Figure 9:**
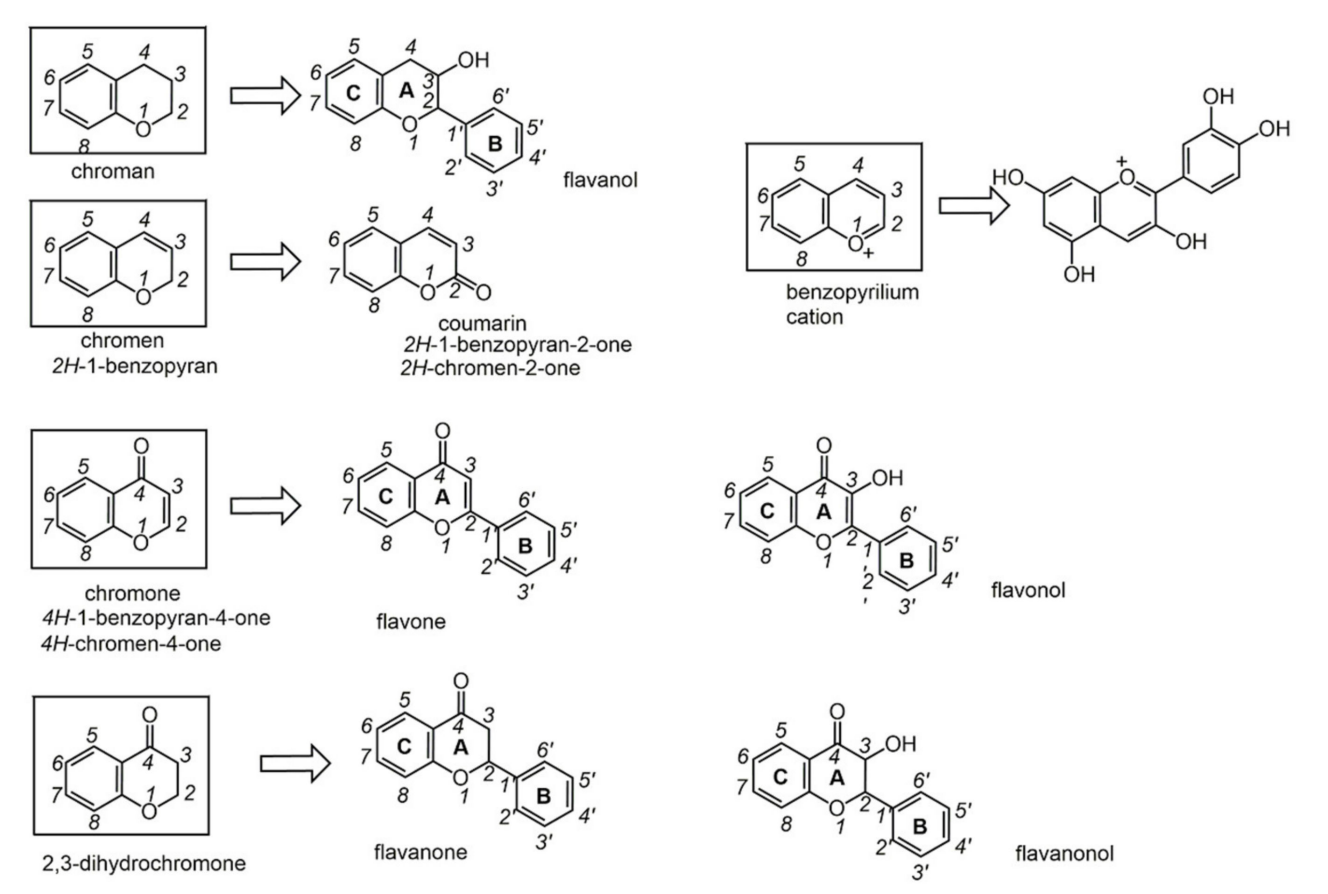
Structures of parent heterocyclic compounds and their phenyl derivatives present in polyphenols.

Polyphenols are characterized by the presence of benzo-fused heteroaromatic ring of the pyrane or pyrilium type. They are usually named by a semi-systematic nomenclature, based on the parent heterocycle. Thus, benzopyrane derivatives with a phenyl substituent are named flavanes, while phenyl substituted benzopyranones are indicated as flavones. The structures of parent compounds and their phenyl derivatives are collected in Figure 9.

The synthesis of natural and semi-synthetic highly oxidized bioactive polyphenols was reviewed in 2008, discussing advances and challenges [369]. Alternatively, more efficient and sustainable production may come from microbial cell factories, as reviewed in 2018 [370].

Chemical transformations of natural phenols might lead to more effective species, if structural features at the basis of biological activity are understood. Thus, results of chemical modifications for representative polyphenols are reported in the following.

### 6.1. Phenols from Chroman

**Figure 10:**
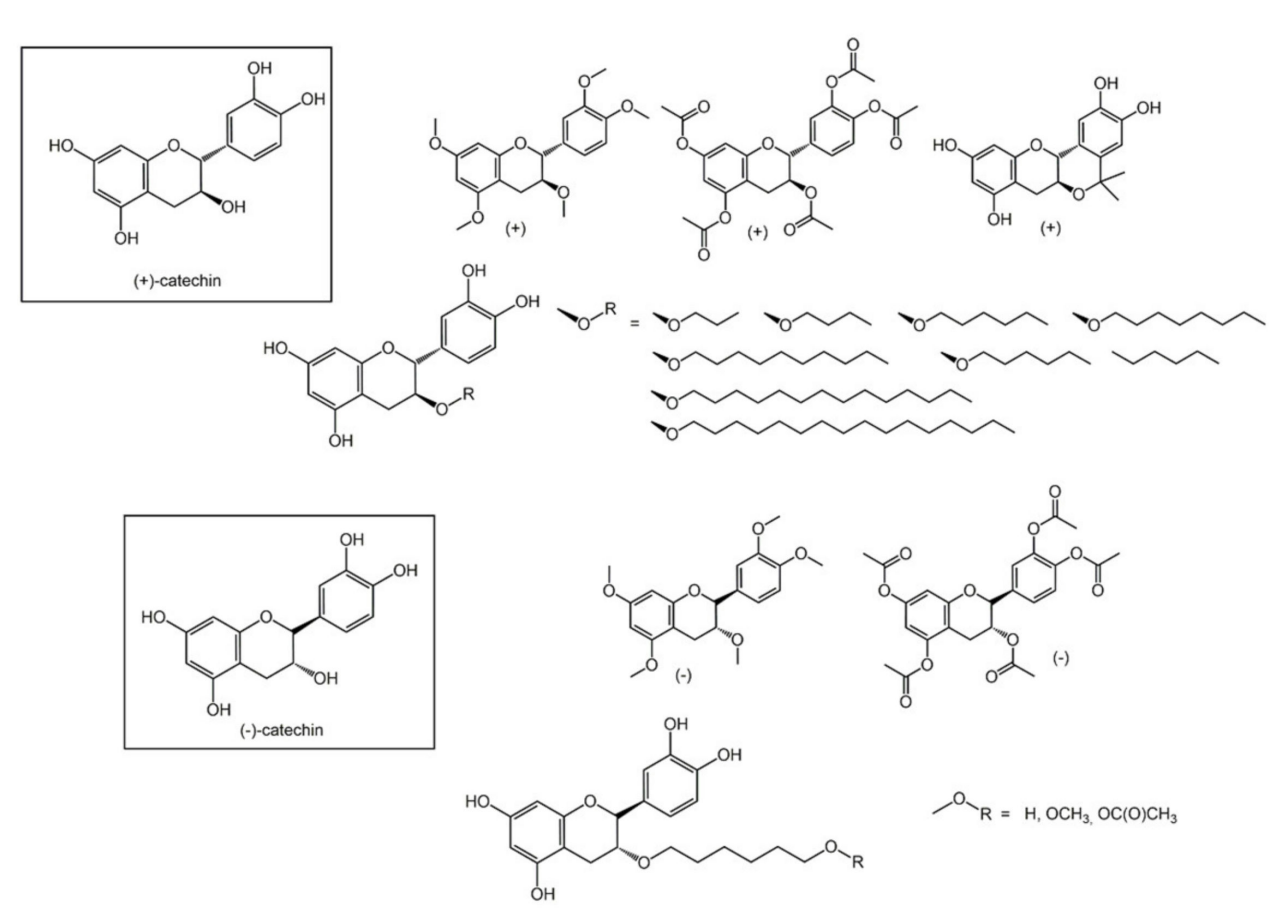
Synthetic derivatives of catechin with enhanced activity [371,372,373].

Cathechin is a polyphenol of the flavanol family that can be found in green tea. Derivatives reported in Figure 10 were prepared from racemic cathechin (tetramethoxy, pentaacetoxy, and cyclic) [371]. Catechin and the derivatives were tested for antimicrobial activity against root-colonizing fungi, which was maintained, although with a lower efficiency, in the less polar compounds.

The antioxidative radical scavenging of (+)-catechin, probed against the galvinoxyl radical, is enhanced upon reaction with ninhydrin [374].

To address the problem of increasing resistance of microorganisms, studies were aimed at preparing and testing synthetic derivatives of catechin. The systematic etherification of 3-hydroxyl group with linear alkyl chains of different length or with substituted benzyl groups gave a library of 3-*O*-alkyl analogues of catechin [372] that were used to test the antifungal activity as a function of structure. Compounds with longer chains (C14–C16) exhibited weaker activities than compounds with C8–C12 chains.

Moreover, varying the -OH functionality in 3, twelve derivatives of (-)-catechin were prepared [373]. Only three compounds showed antibacterial and antifungal activity higher than that of the standard drugs (neomycin and miconazole). Molecular docking studies agreed with experimental results.

**Figure 11:**
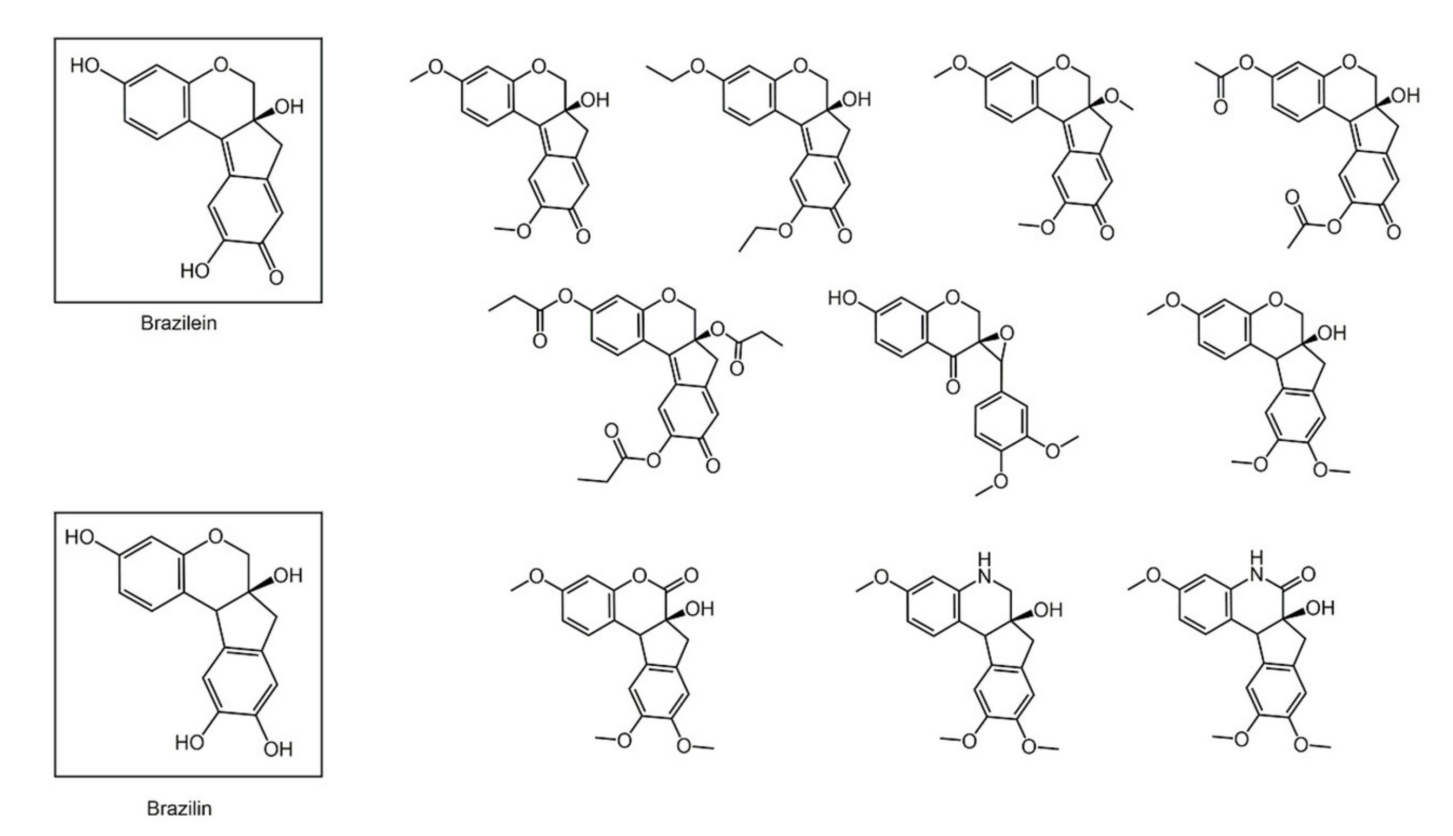
Selected examples of biologically active synthetic derivatives of brazilein [375] and heterocyclic analogues of brazilin [376].

Brazilin and the oxidized analog brazilein (Figure 11) are chromane derivatives found in plants (*Caesalpinia sappan L*.), known for their anti-inflammatory properties. Novel synthetic derivatives were prepared to explore their antitumor activity. The synthesis of brazileins [375] was achieved starting from 1,3-dihydroxybenzene (resorcinol) and 3-chloropropanoic acid, with formation of the key intermediate 7-hydroxy-4-chromanone. The synthesized brazileins (Figure 11) were tested for anti-inflammatory effects and against a number of human cancer cell lines, but only some of the synthetic derivatives showed some improvement with respect to the unsubstituted brazilein.

### 6.2. Phenols from Chromen

Coumarin (2*H*-chromen-2-one), the most common derivative of chromen, and substituted coumarins are found in green plants, where they exert different actions [377].

**Scheme 40:**
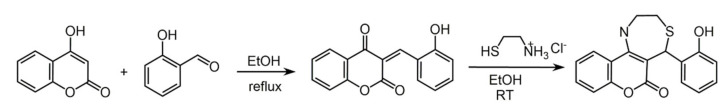
Synthesis of 1,4-thiazepine derivative from 4-hydroxycoumarin [379].

Considering the versatility of reactions leading to coumarin heterocycles system [378], a number of substituted coumarins were synthesized and promising activity was found with coumarin-fused 1,4-thiazepines, synthesized starting from 4-hydroxycoumarins (Scheme 40) [379].

Coumarins and benzocoumarins substituted with hydroxyl group in 7- or 8- positions were prepared and tested in vitro for a number of biological activities [380]. Generally, they delivered potent superoxide anion scavengers and inhibited in vitro lipid peroxidation; on the other hand, they did not show significant lipoxygenase inhibitory activity.

### 6.3. Phenols from Chromon

Quercetin (3,3′,4′,5,7-pentahydroxyflavone) is a flavonol largely present in plants, foods and beverages, often together with fisetin (3,3′,4′,7-tetrahydroxyflavone).

The interest in quercetin was prompted by its anti-cancer, anti-inflammatory and antioxidant roles. Particular relevance is offered by the anti-hypertensive effect [381]. Many synthetic derivatives were prepared with the aim to obtain anti-cancer candidates that might overcome quercetin problems: (*i*) low solubility in water, (*ii*) low bioavailability, and (*iii*) fast degradation. Studies on biological activity were not sufficient to assess the actual effectiveness of those derivatives, although some of them seemed promising [382]. On the other hand, simple complexation with Cu(II) gave a Cu(quercetin)(bipy) complex with enhanced antioxidant properties compared to free quercetin [383].

The interest in therapeutic properties of quercetin and derivatives is not limited to cytotoxicity, as it can be seen by the number of patents reported between 2010 and 2015 [384]. Selected meaningful examples are collected in Table 2.

Most of such derivatives involved transformations at all the hydroxyl groups, with the modification at C-3-hydroxyl group resulting in enhancement of anticancer activity. Moreover, the bioactivity was significantly increased by nanotechnology.

The problem of scarce solubility in water of flavonoids polyphenols was addressed considering derivatives with hydrophilic substituents, such as sulfate [385].

A different approach was to prepare conjugates of sugars with flavonoid being the aglycone. Glucose, galactose and rhamnose were used, inter alia.

Enzymatic synthesis was successful in modifying natural compounds yielding not only more soluble, but also more efficient species in medicinal [386] or cosmeceutical [387] applications.

**Scheme 41:**
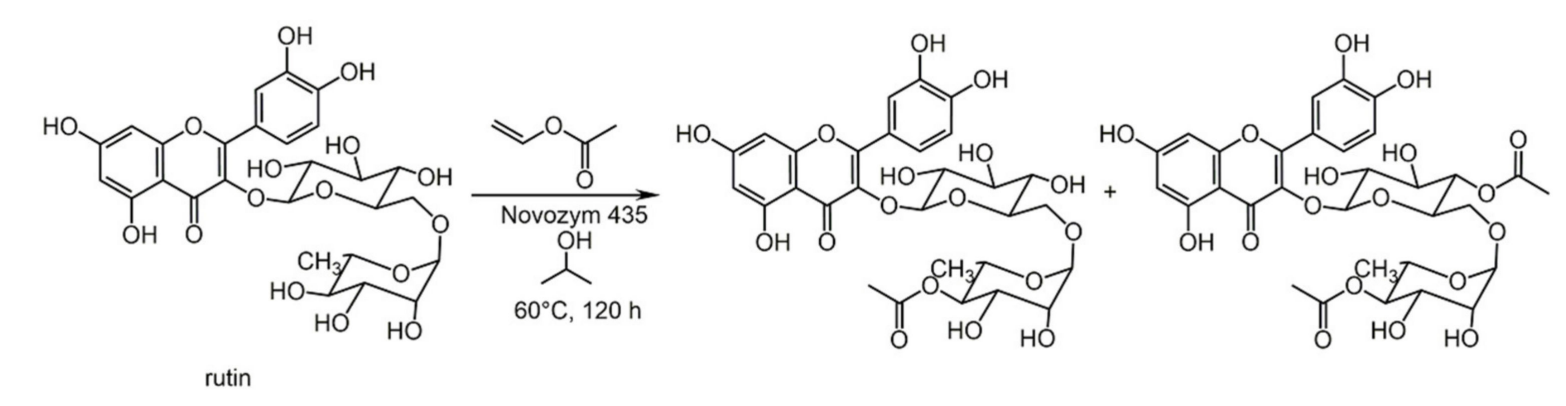
Enzymatic esterification of rutin [388].

It is interesting that rutin (2-(3,4-dihydroxyphenyl)-5,7-dihydroxy-3-[α-*L*-rhamnopyranosyl-(1→6)-*β-D*-glucopyranosyloxy]-4*H*-chromen-4-one) was further transformed by enzymatic transesterification reaction into mono- and di-acetate derivatives (Scheme 41) with maintained anti-oxidant properties and more efficient ability to penetrate the cell membrane of murine macrophages [388]. Moreover, acetoxy-substituted rutins were not toxic to mammalian cells and the enzyme could be reused.

The importance of sugar-substituted flavonoids is exemplified by myricitrin (myricetin-3-*O*-*α*-*L*-rhamnopyranoside), whose anti-oxidant activity had a protective effect against DNA damage [389].

A novel flavonoid scaffold was prepared introducing salicylate and trimethoxybenzene groups in flavonoids [390]. All the compounds were evaluated for antiproliferative activity against three human tumor cells showing moderate to good activity.

### 6.4. Phenols from 2,3-dihydrochromon

Among flavanones, secondary metabolites of plants with a broad spectrum of biological activities, pinostribin (5-hydroxy-7-methoxy-flavanone) received interest because it is the major component in the rhizome of fingerroot (*Kaempferia pandurata*), used in Southeast Asian cooking, known to have several pharmacological activities, among which the antimicrobial one is promising.

**Scheme 42:**
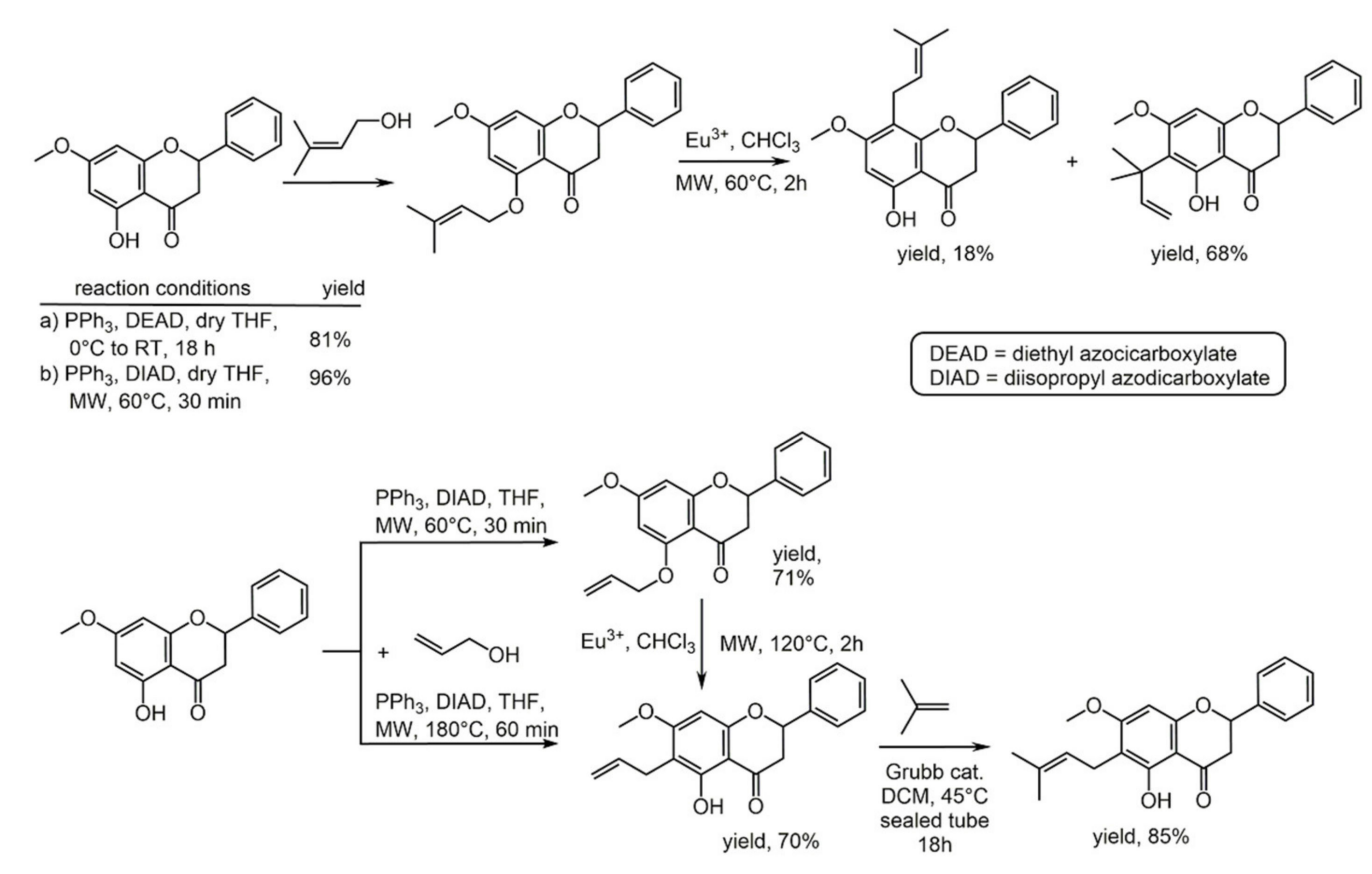
MW-assisted prenylation of pinostribin [391].

Allylation and prenylation of pinostrobin were performed using MW irradiation (Mitsunobu and metathesis reactions, Claisen and Cope rearrangements) (Scheme 42), giving compounds that were tested against a number of cancer cell lines [391]. The derivatives were more reactive than pinostribin, a result that the authors attributed to a better interaction with the biological targets, due to the increased lipophilicity provided by alkenyl substituents.

**Scheme 43:**
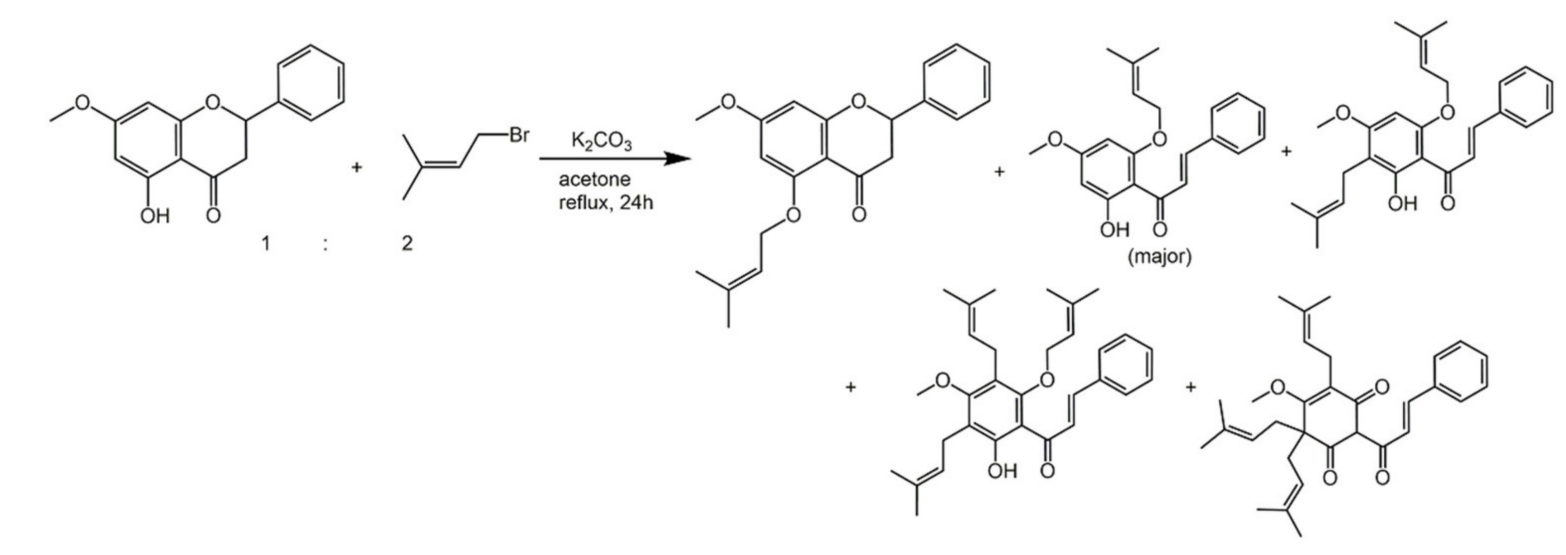
Prenylation of pinostribin [392].

Pinostribin was prenylated under simple S_N_2 conditions (Scheme 43), giving a mixture of products, most of which lost the flavanone structure. They were separated and tested for antimicrobial activity [392], showing a moderate effect. Interestingly, several prenylated coumarins and quercetins were isolated from the root bark of *Broussonetia Papyrifera* as metabolites with, in some cases, cytotoxic activity [393].

**Scheme 44:**
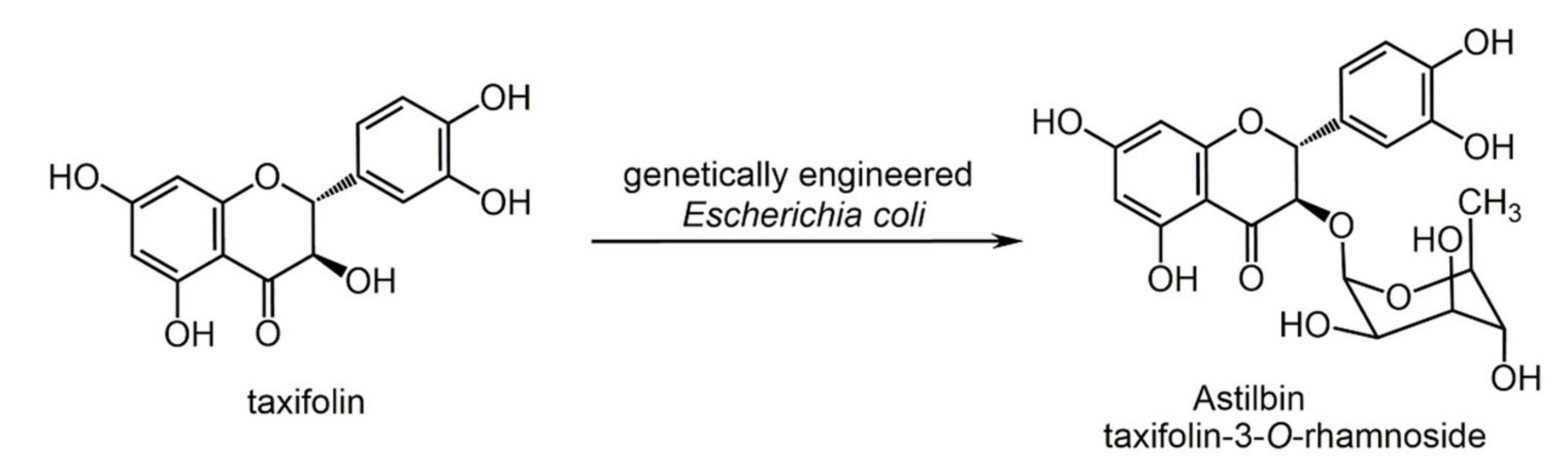
Astilbin from taxifolin by microbial fermentation [394].

Astilbin, a sugar derivative of the flavanol taxifolin, is extracted from herbal medicinal plants, commonly used in traditional Chinese medicine. However, for their possible pharmaceutical use, the astilbin available from extraction is not sufficient. An efficient process to obtain astilbin from taxifolin relied on microbial fermentation in genetically-engineered *Escherichia coli* (Scheme 44) [394].

A cascade biocatalytic system was developed to prepare the 4′-*O*-glucoside derivatives of naringenin (5,7-dihydroxyflavanone), a flavonoid with several bioactive effects, found in grapes and oranges [395]. The method relies on regenerating uridine diphosphate from sucrose and reusing it, performing also preparative scale production. By the same method, quercetin 7-O-*α*-L-rhamnoside was obtained.

Interestingly, myricitrin (myricetin-3-*O*-*α*-L-rhamnopyranoside) [389] and naringenin, present in extracts of *Cynara cardunculus*, a powerful natural herbicide, exhibited phytotoxic effects on the leaves of *Trifolium incarnatum*, opening the way to natural herbicides, a field increasingly important due the increasing resistance of weeds to commonly used ones [396].

## 7. Curcumin and Curcuminoids

**Scheme 45:**
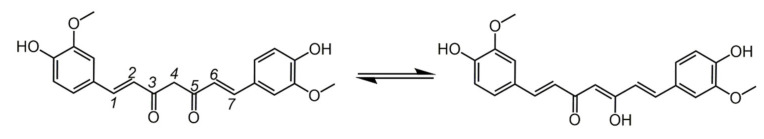
Keto-enol equilibrium of curcumin.

Curcumin, [1,7-bis(4-hydroxy-3-methoxyphenyl)-1,6-heptadiene-3,5-dione], a yellow pigment isolated from turmeric (*Curcuma longa* Linn), is a multifunctional compound that, at least from reading the literature of the past twenty years, seems a sort of panacea for all the illness of modern society, cancer and Alzheimer’s disease included. Phenolic –OH groups ensure the anti-oxidant properties, whereas the extensive conjugation due to the keto-enol equilibrium (Scheme 45) is the basis of photodynamic activity.

Several recent reviews discuss the aspects of biological activity [397,398,399,400] and possible medical applications [70,292,401,402,403,404] of curcumins and derivatives. Important aspects, as new delivery methods and synergistic effects with other compounds, were also discussed, together with the mechanism of action [405]. An increasing interest is devoted to curcumin-based drugs against neurodegenerative diseases [406], especially Alzheimer’s [407] and cancer [408].

The quest for new curcumin derivatives is motivated by (*i*) the necessity of increasing the material availability, and (*ii*) the necessity of meliorating the solubility in aqueous solution.

Notably, just treating curcumin with Cu(II), a Cu(curcumin)(bipy) complex was isolated and it was a better antioxidant and DNA binding compared to free curcumin, while being less toxic, on the basis of its antifungal properties [383]. It was also reported that silver–curcumin nanoconjugates, prepared by a sonochemical method, were tested on skin cell lines and for antibacterial activity against *Escherichia coli*. Results indicated that silver nanoparticles were made biocompatible by curcumin, while they make curcumin more photostable and more active as an antibacterial [409].

We discuss curcumin derivatives according to structural changes.

### 7.1. Minor Structural Changes

Small structural changes can alter the efficiency of curcumin bioactivity. For example, introducing one methyl group in position 2 or two methyl groups, as in 2,7-dimethylcurcumin, enhanced anti-angiogenesis activity and suppression of tumor growth [410] were observed, together with increased anti-inflammatory activity [411] and stability against enzymatic reduction [412], with respect of curcumin.

Diacetylcurcumin, easily prepared by acetylation of the parent compound, showed excellent antibacterial activity [413] and it was effective in antiarthritic activity in mice (Table 3) [414].

The anti-oxidant activity of curcumin was compared with that of demethoxy metabolites and of hydrogenated derivatives [415]. Results indicated that the saturated derivatives (tetrahydro-, hexahydro- and octahydro- coumarins) have increased antioxidant activity with respect to coumarin (Table 3).

The derivative obtained by the introduction of prenyl substituents in both aromatic rings was tested against oxidative stress [416] (Table 3), showing equal or better antioxidant properties with respect to curcumin.

A more drastic substitution was performed, introducing electron withdrawing substituents into benzene rings, or even condensing heterocycles (Table 3) [417].

### 7.2. Substituents in the Unsaturated Chain

**Figure 12:**
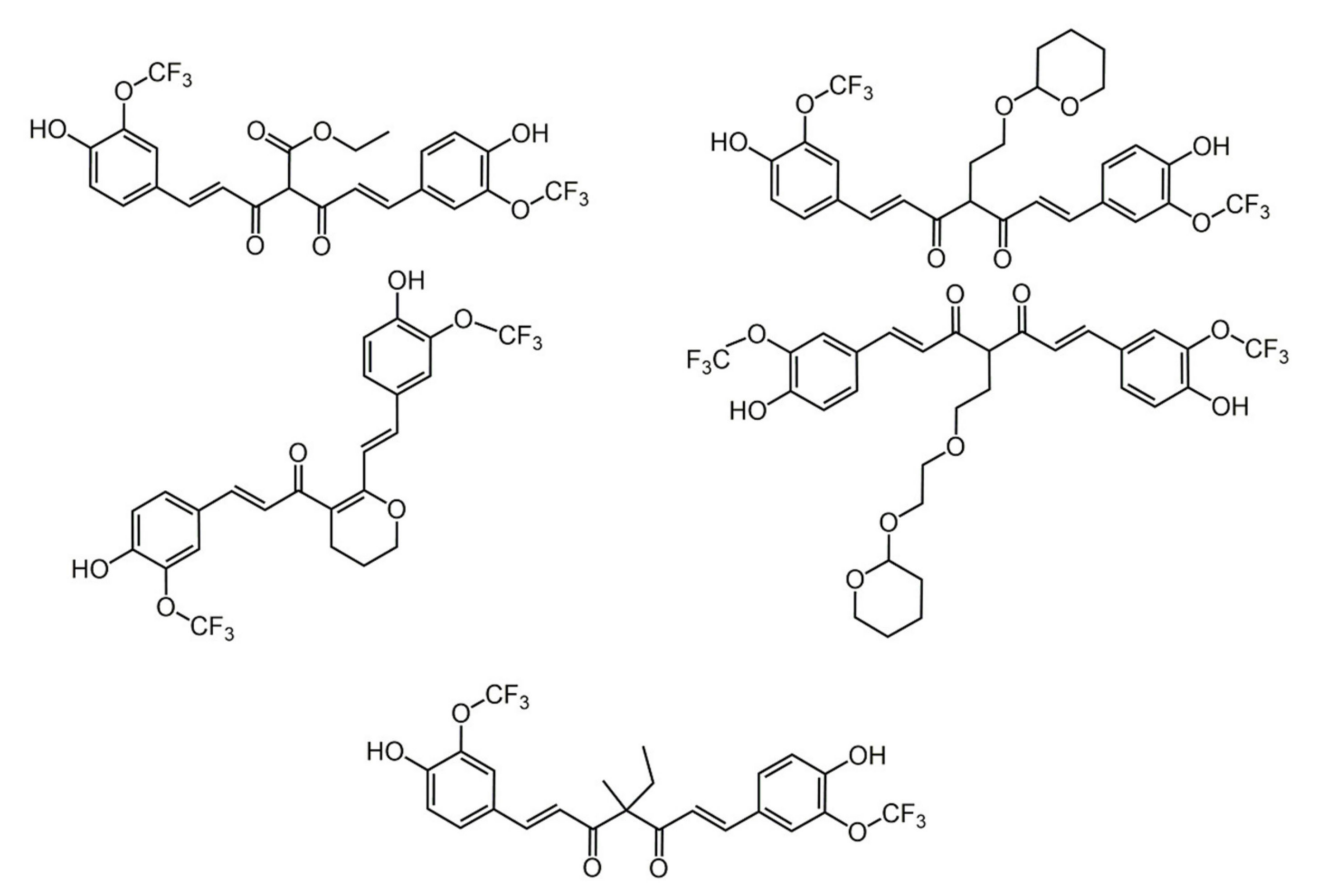
The 4-Substituted curcumins that inhibit the formation of large amyloid aggregates [434] and 4,4-disubstituted curcumin that binds amyloid oligomers [435].

Most of synthetic derivatives come from the introduction of substituents in position 4, thus affecting curcumin tautomeric equilibrium. An important bioactivity linked to the keto-enol equilibrium is the interaction with amyloid β (Aβ) aggregation, present in Alzheimer’s disease. An extensive investigation of keto-enol tautomeric equilibria in substituted curcumins was performed [432,433,434]. Recently, it was reported that 4,4-disubstituted curcumin (Figure 12), where the keto form is the only possible one, binds non-fibrillar soluble Aβ oligomers, becoming, as the authors state, “a first-generation compound targeting Aβ oligomers” [435].

Fluorinated curcumin derivatives showed significant inhibition of the thioredoxin-interacting protein (TXNIP), which is associated with multiple diseases [418].

**Figure 13:**
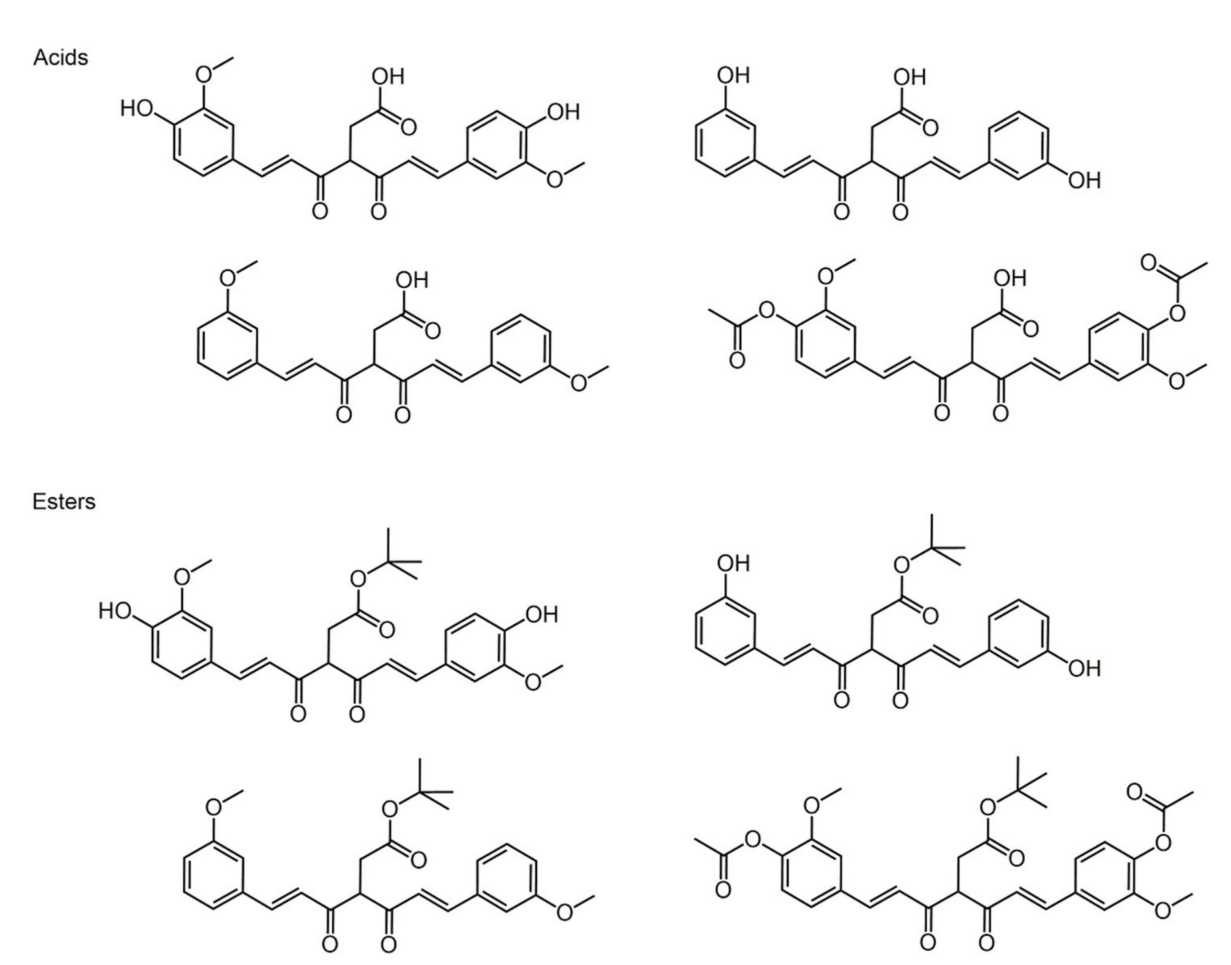
Curcumins 4-substituted with acid or ester groups [436].

A number of curcumin derivatives substituted by acid or ester groups in position 4 were prepared [436] (Figure 13). Acidity, lipophilicity and kinetic stability were determined, together with free radical scavenging activity, in order to evaluate any relation between structure and activity. The ester derivatives exhibited selectivity against colon carcinoma cells, probably as a result of their higher lipophilicity thank curcumin.

A different approach was the introduction of an unsaturated moiety in position 4 (Table 3), by Knøvenagel reaction with benzenecarbaldehyde, 4-hydroxybenzaldehyde and 4-hydroxy-3-methoxy-benzaldehyde (vanillin) [419]. Obtained derivatives were tested for anti-malaria activity against *P. falciparum*, and the vanillin derivative was considerably potent. 4-Benzylidene curcumins, prepared from 2-hydroxybenzenecarbaldehyde [420] and 4-benzylidenecurcumins, were investigated as anti-oxidant agents and both were effective in attenuating the cataractin cultured rat lenses.

### 7.3. Modification of the β-dicarbonyl Moiety

Eighteen new derivatives, still featuring the hepta-1,6-dien-3,5-dione structure of curcumin, but with one of the carbonyl groups incorporated into the cycloheptanone moiety, were synthesized by a versatile synthetic strategy [421]. One example of substituted tropanone is reported in Table 3. The authors are confident that the family of dicarbonyl curcumins with the tropane ring will have important activity, since the simple monocarbonyltropanones were cytotoxic towards breast cancer cells.

**Scheme 46:**
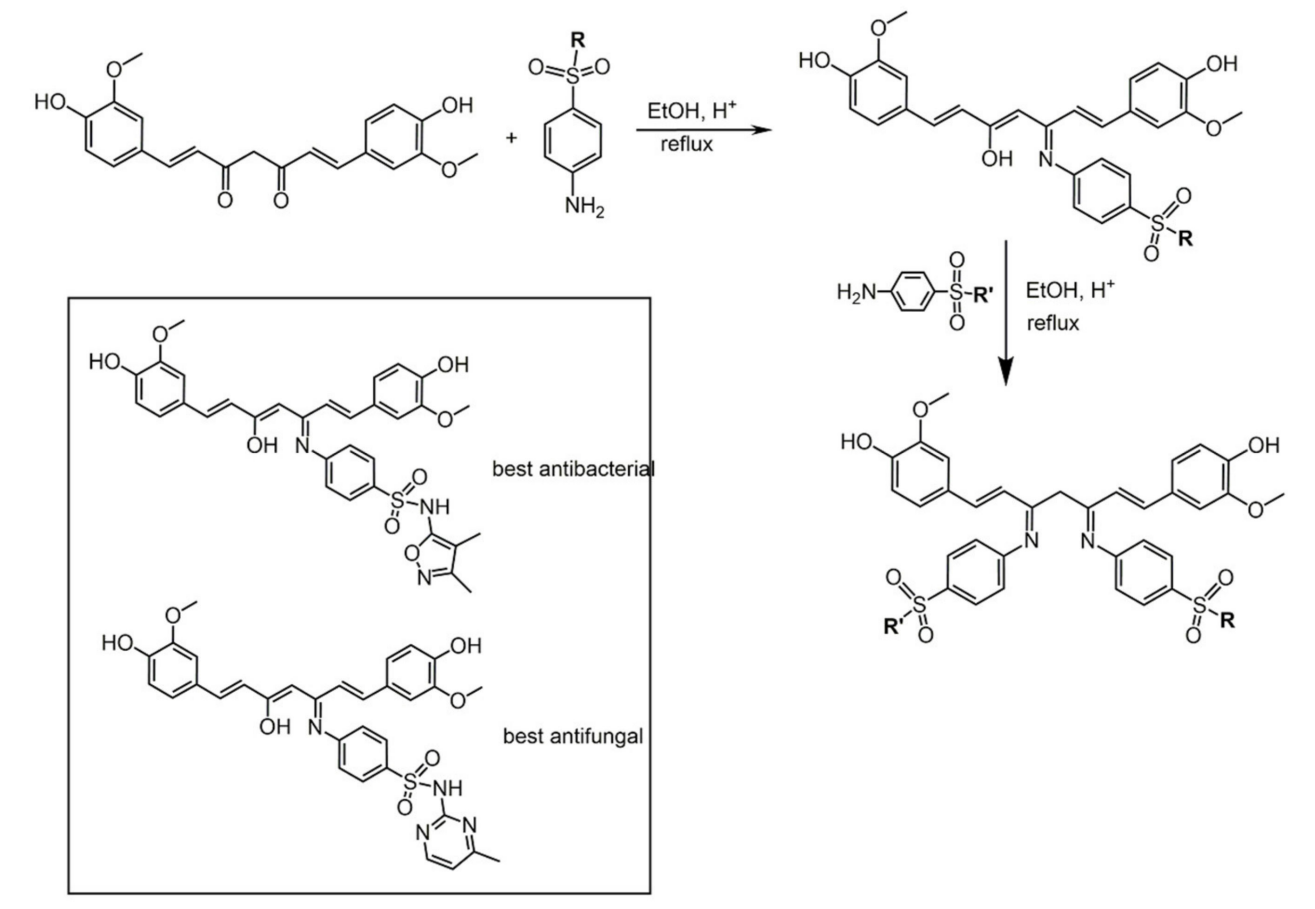
Sulfonamido analogues of curcumin [437].

A library of curcumin derivatives was obtained upon reaction with one or two equivalents of sulfonamides (chosen among sulfa drugs) (Scheme 46) [437]. Antibacterial and antifungal activities were evaluated against Gram-positive and Gram-negative microorganisms, with good results.

3,4-Dihydropyrimidin-2(1*H*)-one and thione analogues of curcumin (Table 3) were synthesized in good yield by a one-pot multi-component cyclocondensation under MW irradiation [425]. Antibacterial and antioxidant studies were performed in vitro with results considered by the authors “moderate” in the former case and “excellent” in the latter.

A pyrazole derivative of curcumin was prepared trying to incorporate in the same molecule the structural features of curcumin and of a steroid-like compound (cyclohexyl bisphenol A) [422]. The compound was found to be neuroprotective in cell culture assays, also against intracellular and extracellular amyloid. Moreover, it was found to possess memory enhancing properties in a rat object recognition test [423].

### 7.4. Partial Replacement of the β-dicarbonyl Moiety

**Scheme 47:**
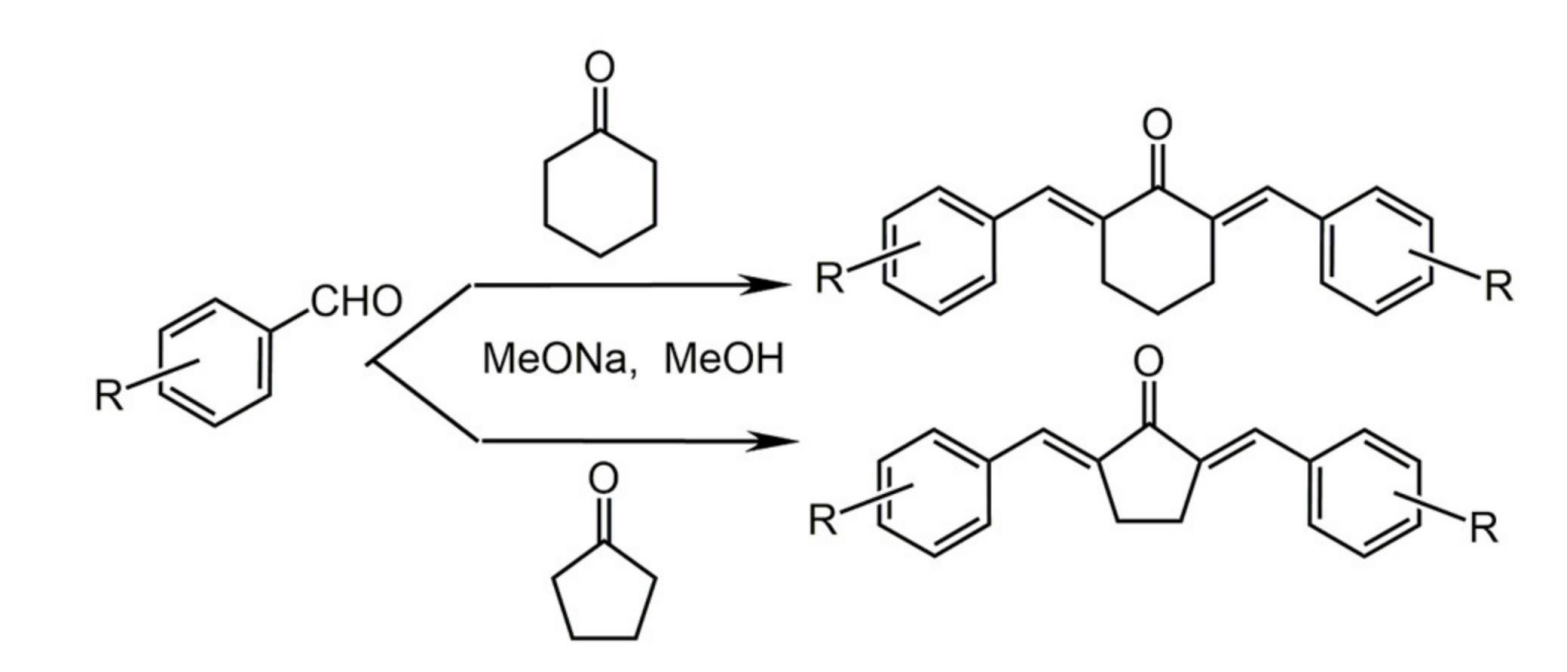
Synthesis of monocarbonyl curcuminoids [439].

Partial replacing of the *β*-dicarbonyl moiety of curcumin was considered useful to overcome the problem of its unsatisfactory stability. A series of mono-carbonyl analogues of curcumin, synthesized from the opportunely substituted benzaldehyde and a cycloalkanone [438,439,440]. The stability of the substituted cyclopentanones and cyclohexanones was enhanced in vitro. The cytotoxic activity was also higher with cyclohexanones, with a remarkable importance of substituents electronic effects (Scheme 47).

The monocarbonyl curcumin analogs were tested against pro-inflammatory cytokines, exhibiting a more potent inhibitory ability than curcumin.

Symmetrical bis(arylidene)ketones were prepared by reacting cycloalkanones with substituted benzaldehydes, in an acid-catalyzed aldolic condensation. Most of the synthesized compounds showed inhibition of ovarian cancer cell growth, even with cells resistant to cisplatin [441].

Several synthetic monocarbonyl curcumin analogues were tested against *Trichomonas vaginalis* (considered the “most common non-viral sexually transmitted infection in the world”) [442]; 1,5-diphenylpenta-1,4-diene-3-one, 1,5-bis(2-chlorophenyl)penta-1,4-diene-3-one and 2,6-bis(2-chlorobenzylidene)cyclohexanone presented significant antiparasitic activity at effective concentrations lower than that of curcumin.

More recently, a first, but very promising, result was obtained with (2*E*,6*E*)-2,6-bis(2(trifluoromethyl)benzylidene)cyclohexanone, which was found to heal diabetic wound in mice [426] (Table 3).

A dozen curcumin monocarbonyl analogues were synthesized, in order to find compounds with increased chemical stability and, eventually, better anticancer activity against some human cancer cells [427]. Two of them (Table 3) met the requirements and were successively tested against melanoma cells, resulting selectively toxic [428].

**Scheme 48:**
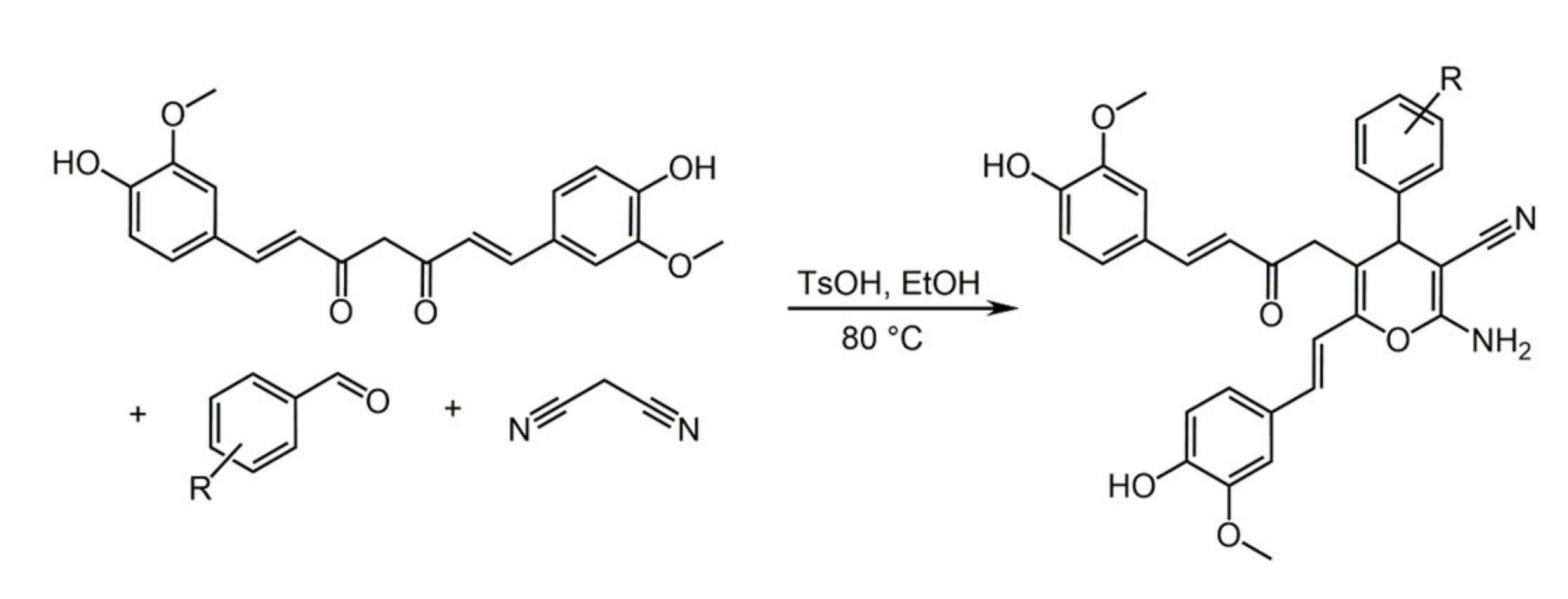
One-pot synthesis of curcuminoids incorporating 4*H*-pyran [443].

New curcuminoids incorporating 4*H*-pyran heterocycles were prepared by one-pot condensation of curcumin with propanodinitrile and a substituted benzenecarbaldehyde (Scheme 48) [443]. The consequent modification of *β*-dicarbonyl moiety improved inhibition of the α-glucosidase, one of the enzymes responsible for carbohydrate hydrolyses and therefore for postprandial hyperglycemia. This feature, together with antioxidant activity, has possible beneficial consequences against diabetes mellitus, especially because no toxic effect was observed on the common human intestine microflora.

### 7.5. Reducing the Length of Unsaturated Chain

A curcumin analogue, 5-(3,4-dihydroxyphenyl)-3-hydroxy-1-(2-hydroxyphenyl)penta-2,4-diene-1-one showed anti-inflammatory activity in mice (Table 3) [429]. Similar compounds with the same skeleton were used to establish the importance of reactive oxygen species upregulation in suppression of tumorigenesis [444]. According to the authors, these compounds are promising for the development of an anti-cancer drug with few side effects.

**Scheme 49:**
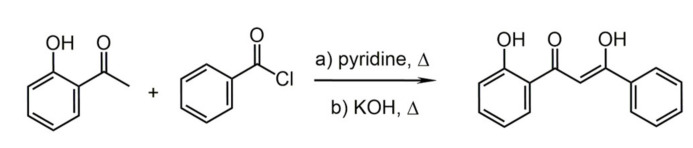
Synthesis of (*Z*)-3-hydroxy-1-(2-hydroxyphenyl)-3-phenylprop-2-en-1-one [445].

A similar but shorter compound, (*Z*)-3-hydroxy-1-(2-hydroxyphenyl)-3-phenylprop-2-ene-1-one, was prepared starting from 2-hydroxyphenyl methyl ketone and benzoyl chloride (Scheme 49). The resulting molecule showed selective cytotoxicity on breast cancer MCF-7 cell [445], human colon cancer cell line [446] and human osteosarcoma cells [447].

### 7.6. Derivatives with Only “Half” of the Curcumin Structure

**Scheme 50:**
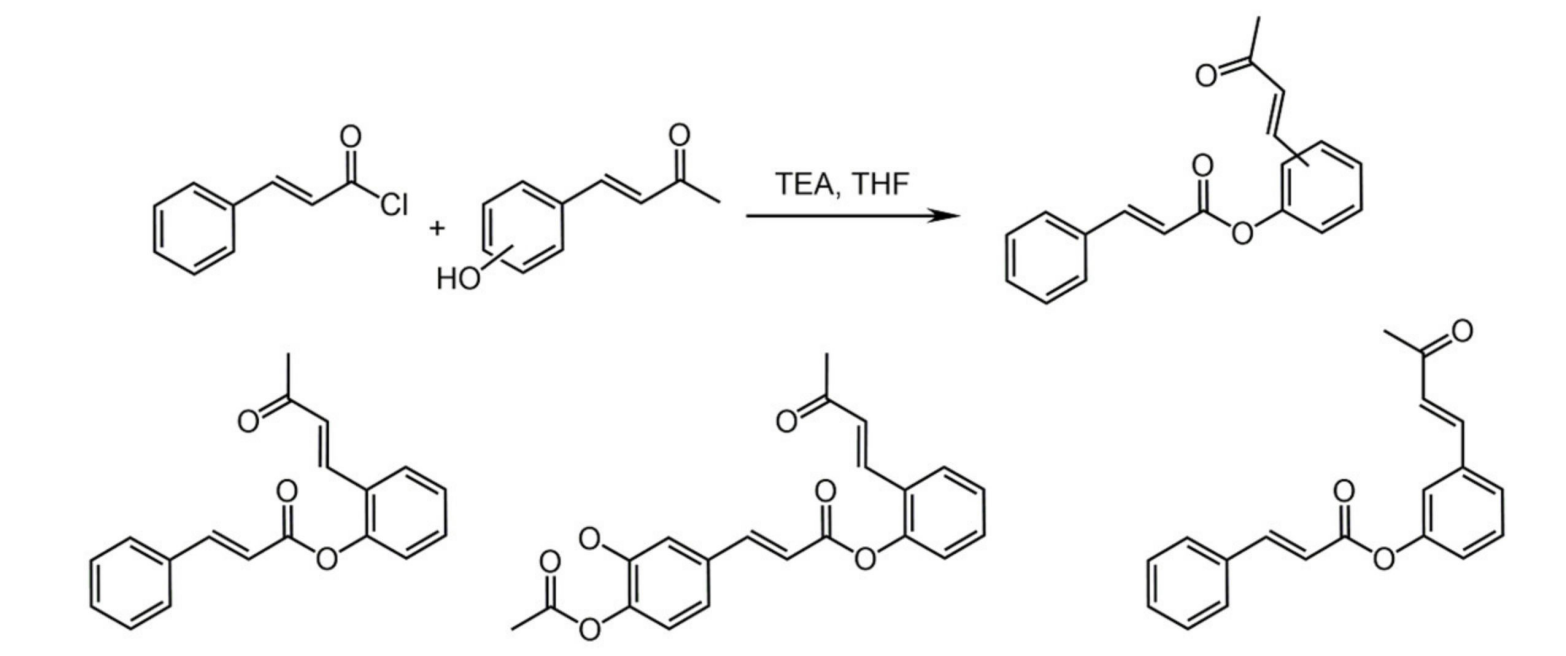
Synthesis of retro-curcuminoids and compounds with cytotoxic properties [448].

A family of compounds, named by the authors retro-curcuminoids, was prepared maintaining only “half” of the curcumin structure (Scheme 50), because the *β*-dicarbonyl moiety was considered responsible for curcumin scarce stability [448]. Resulting compounds showed relevant cytotoxic activity against human cancer cell lines, but they did not injure healthy cells.

A synthetic amide analogue showed anti-oxidative and anti-inflammatory properties. It was tested with good results on hepatic steatosis in mice with induced obesity [430] (Table 3).

**Scheme 51:**
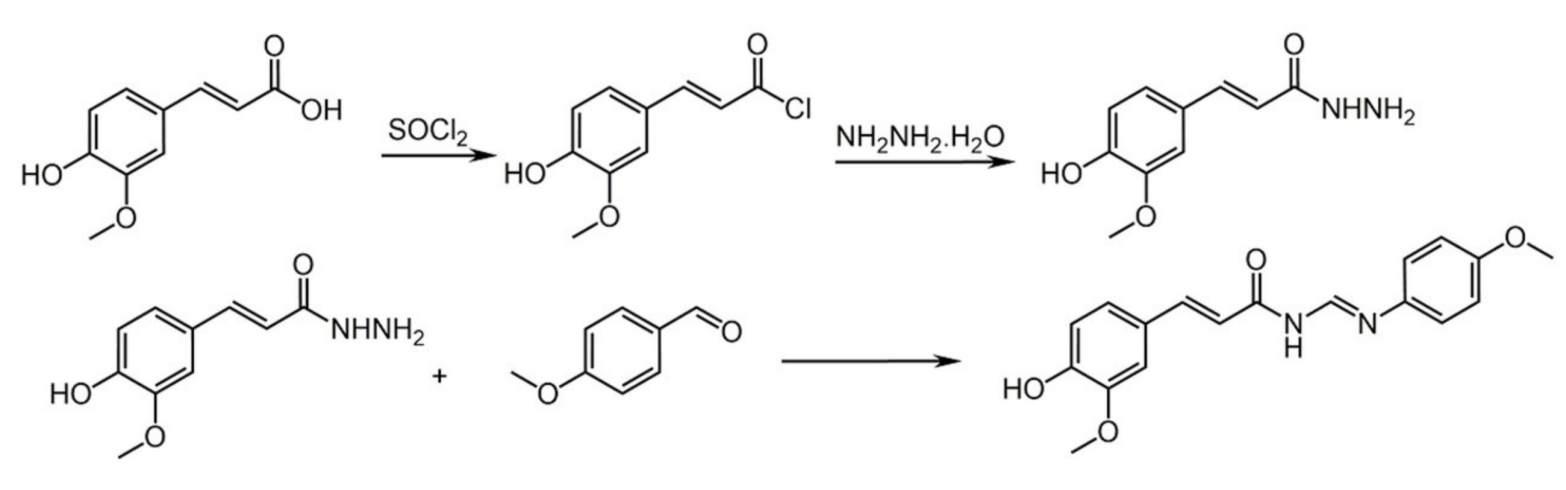
Synthesis of a curcumin–resveratrol hybrid [449].

A library of curcumin–resveratrol hybrids was synthesized starting from the hydrazide derivative of substituted cinnamic acid and a series of substituted benzaldehydes [449]. The example illustrated in Scheme 51 refers to the most promising hybrid as an antitumor multi-target agent.

### 7.7. Photosensityzers

**Figure 14:**
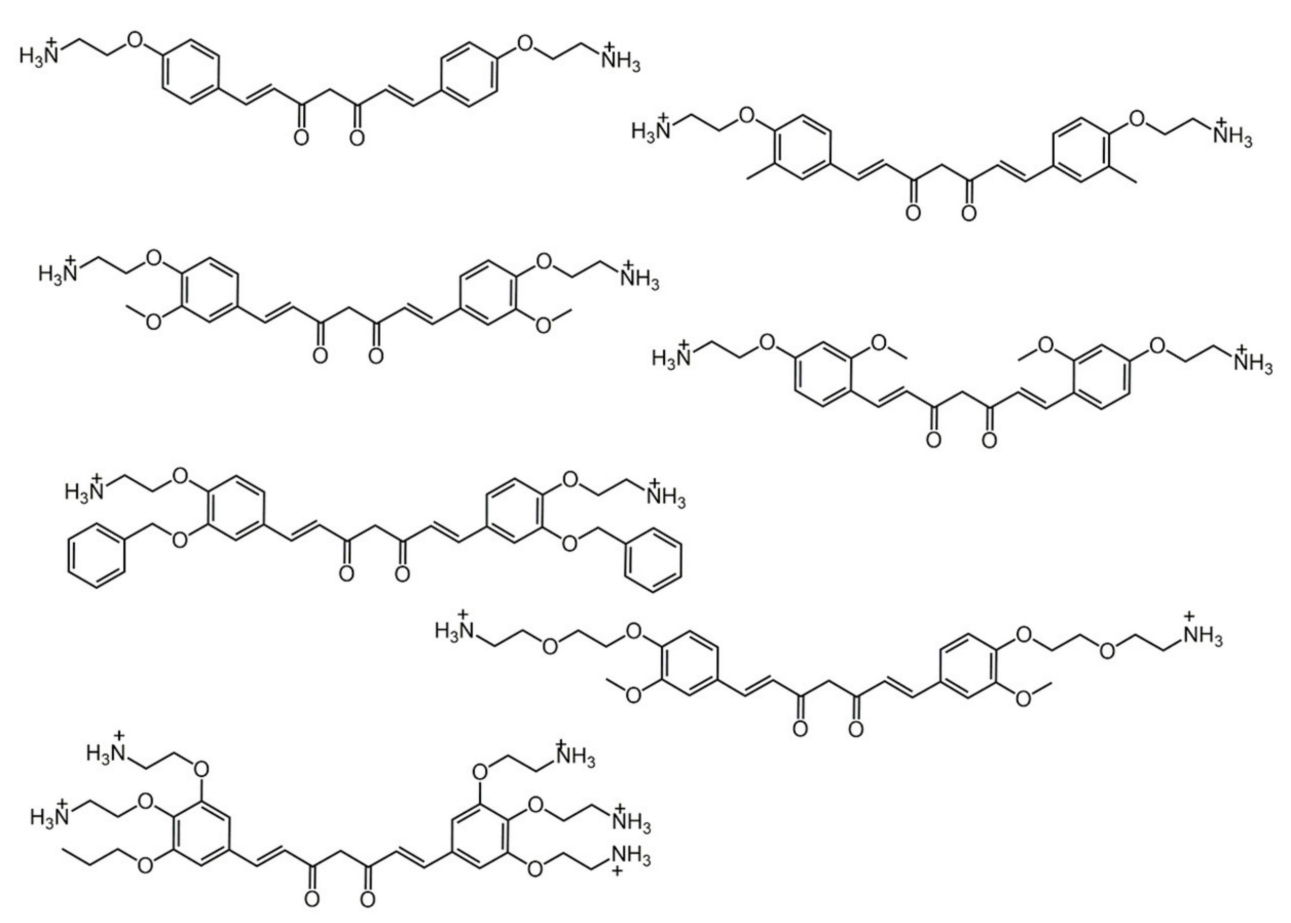
Cationic curcumin derivatives with interesting antimicrobial photodynamic activity [450].

Curcumin might be an excellent photosensitizer, due to its good biocompatibility, but the practical use is strongly limited by its low stability and scarce solubility in water. A solution was searched preparing curcumin derivatives with cationic substituents [450] (Figure 14).

All the derivatives showed high stability with pH and temperature. As to photodynamic properties, they were able to promote photodynamic inactivation of *E. coli*, with the hexa-cationic species being the most effective, probably because of the high hydrophilicity.

A comparative study was performed on different curcumin derivatives synthesized ad hoc, with the aim to increase the tissue penetration, increasing the absorption maximum. Thus, 1,11-diphenyl-1,3,8,10-undecatetraene-5,7-dione and 1,7-bis(4′-dimethylaminophenyl)-1,6-heptadienyl-3,5-dione presented promising characteristics in terms of generating reactive oxygen species and, therefore, of efficacy in photodynamic therapy [431].

## 8. Conclusions

Natural phenols and their derivatives with biological activities constitute a fast-growing research topic, in view of their many present and future applications. Their structural diversity offers many possibilities of chemical transformations, aimed at overcoming the drawbacks of natural phenols. However, apart from some guidelines that emerged from the huge number of publications, such as the need to meliorate stability and bioavailability of the bioactive compounds, the picture of structural requirements is not yet complete, in view of optimizing in vivo and in-field applications.

## Figures and Tables

**Table 1:** Examples of chemically synthesized bioactive lipidic phenols.

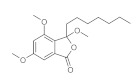	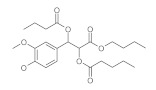
chemotherapy adjuvant [353]	moderate antioxidant activity [354]
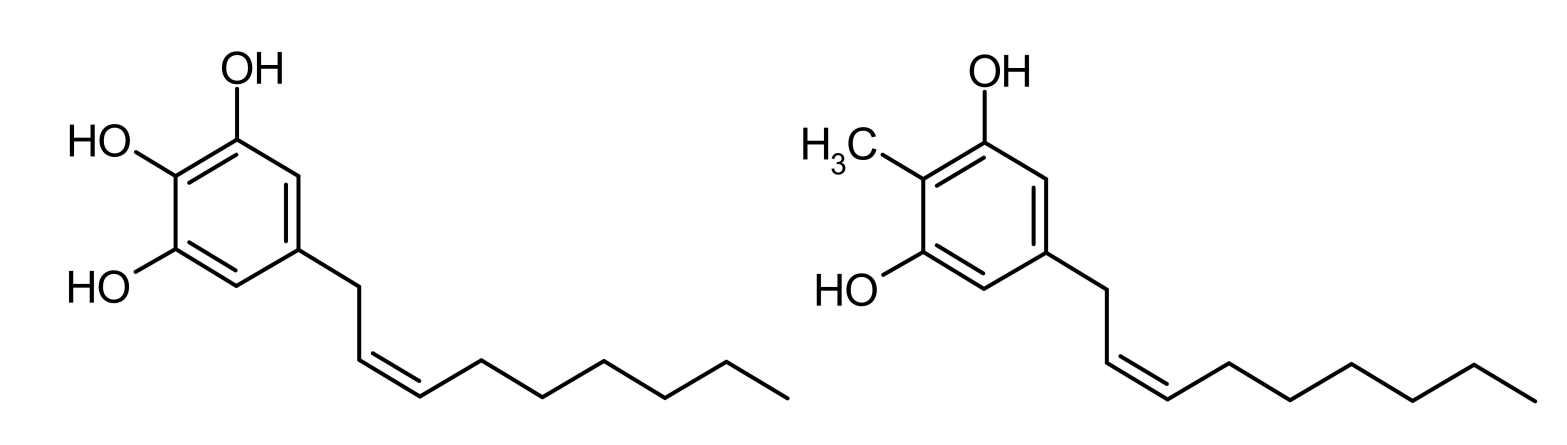	
defense chemical [355]	
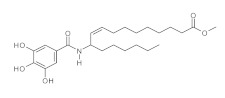	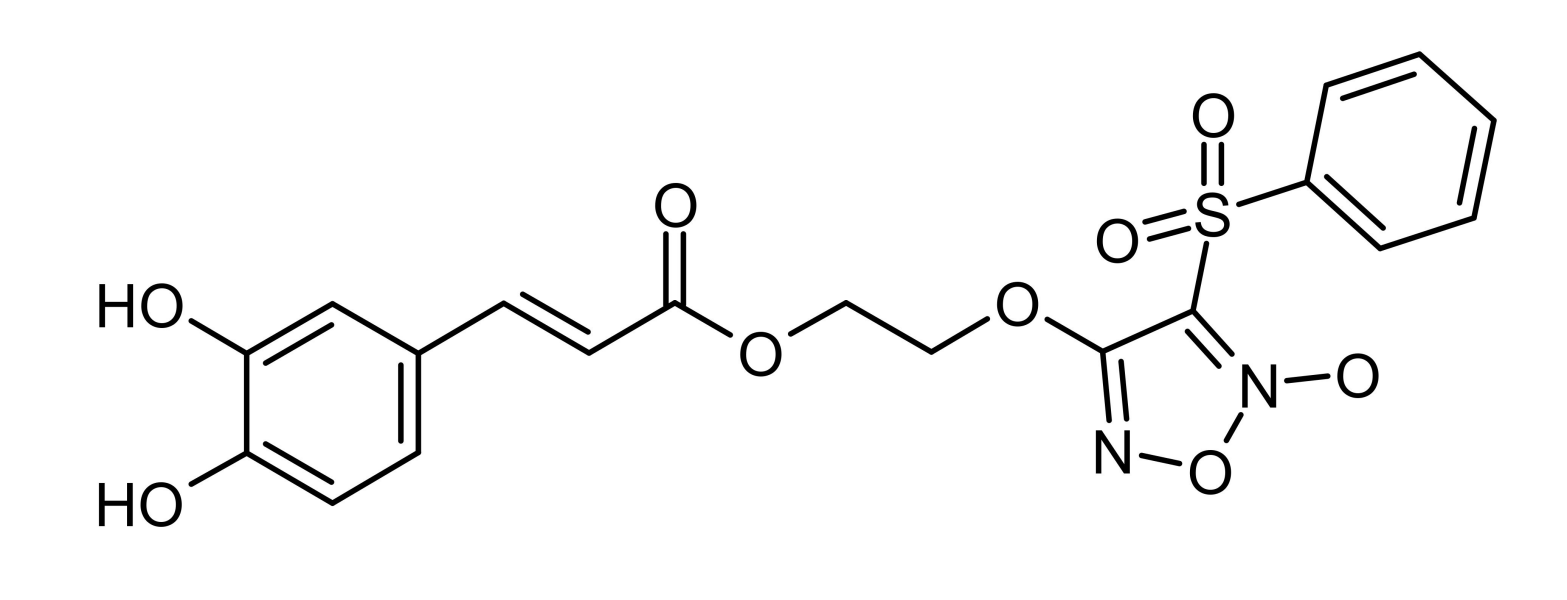
antioxidant and anticancer activity [356]	anticoagulant and vasodilatation effects [357]
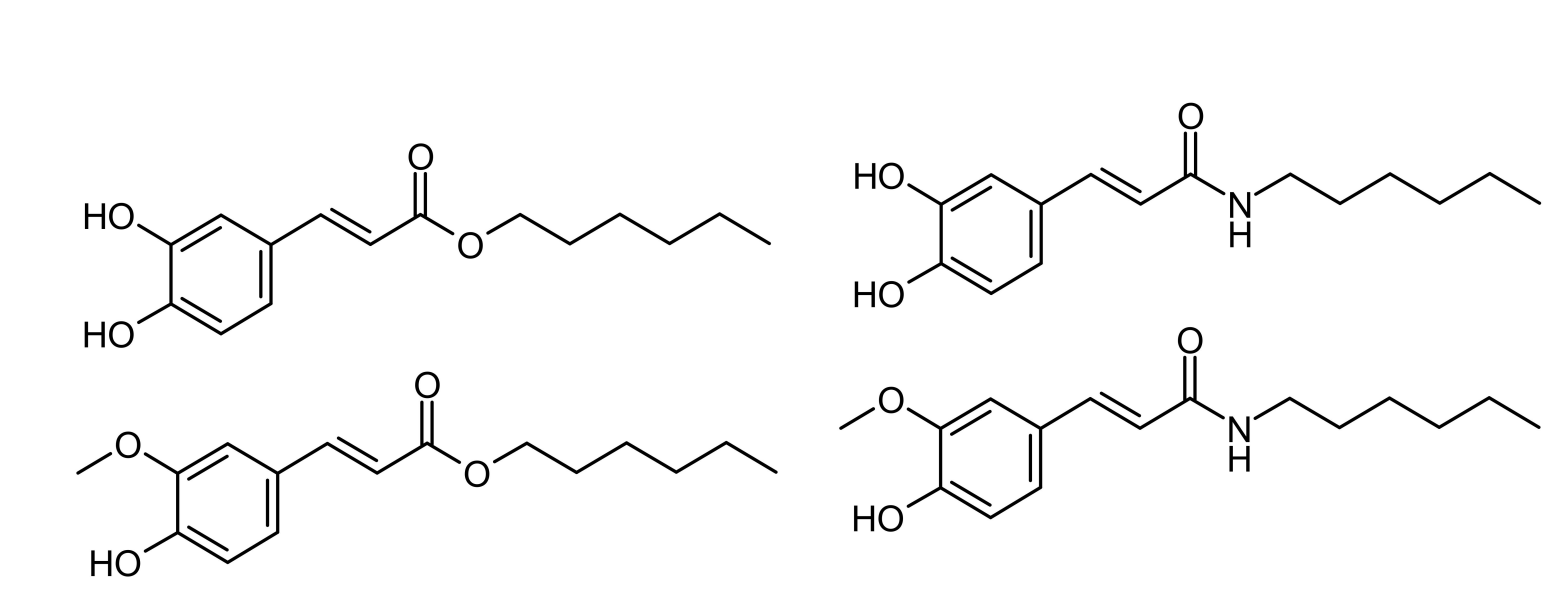	
inhibit cell proliferation, induce cell death in human breast cancer cell lines [320,358]	
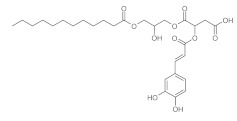	
non-toxic antioxidant [359]	

**Table 2:** Selected examples of quercetin and derivatives with significant therapeutic properties [384].

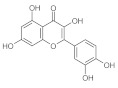 Quercetin *antiviral activity* against DENV-2 used in treating a dengue virus infection	
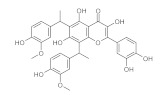	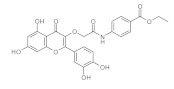
*anticancer*/*antiproliferative activity* (possible treatment of oral cancer)	*anticancer*/*antiproliferative activity* (activity against human esophageal squamous cell EC109)
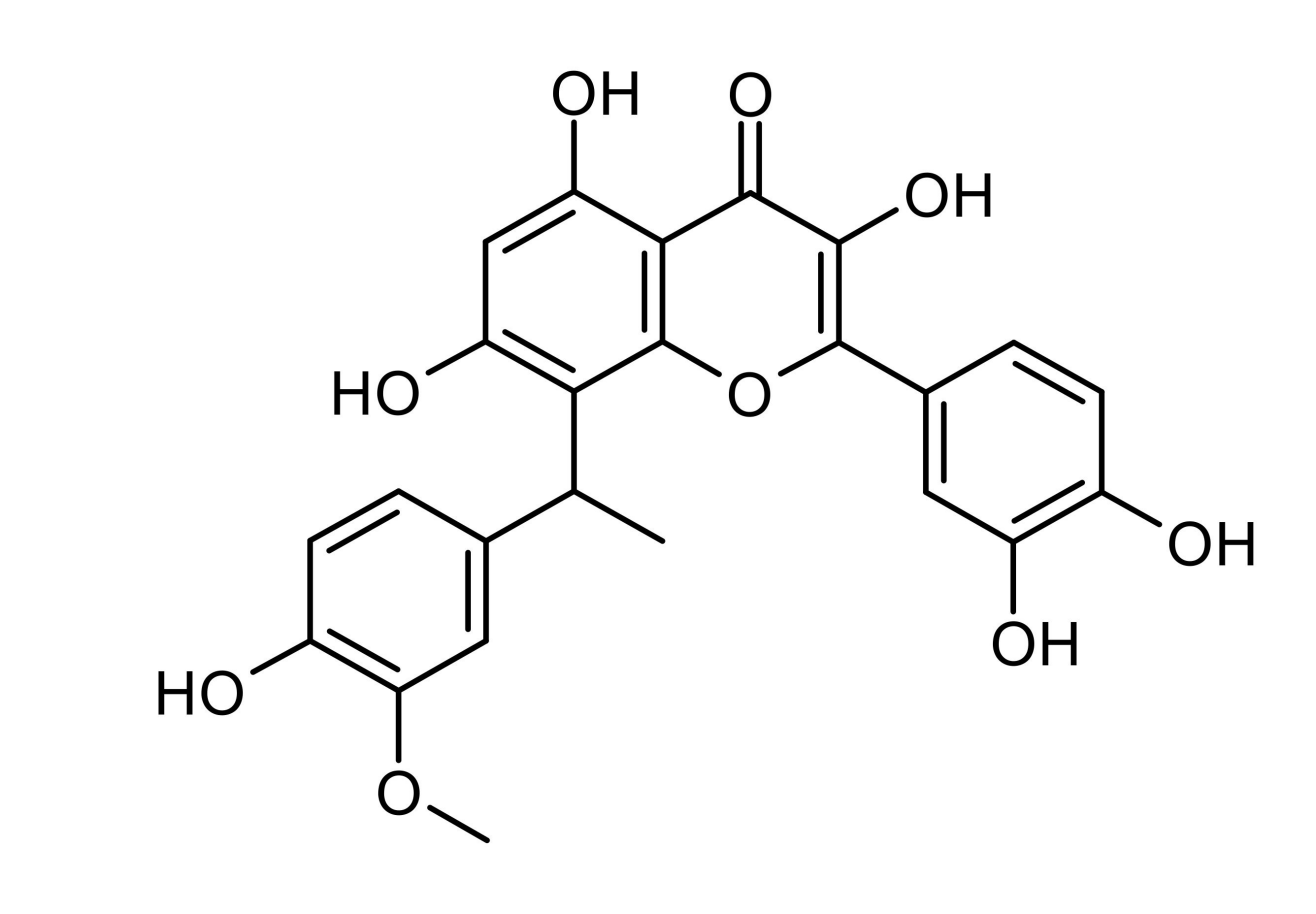	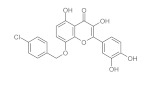
*anticancer*/*antiproliferative activity* (quasi-drugs to reduce the risk of developing cancer)	*antiviral activity* (prophylaxis or treatment of hepatitis C virus)
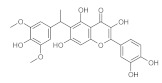	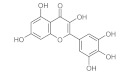
*anti-inflammatory activity* (inhibiting the production of the inflammatory mediator TNF-α)	*cardioprotective activity* (suppressing the activity of the human chymase)
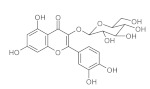	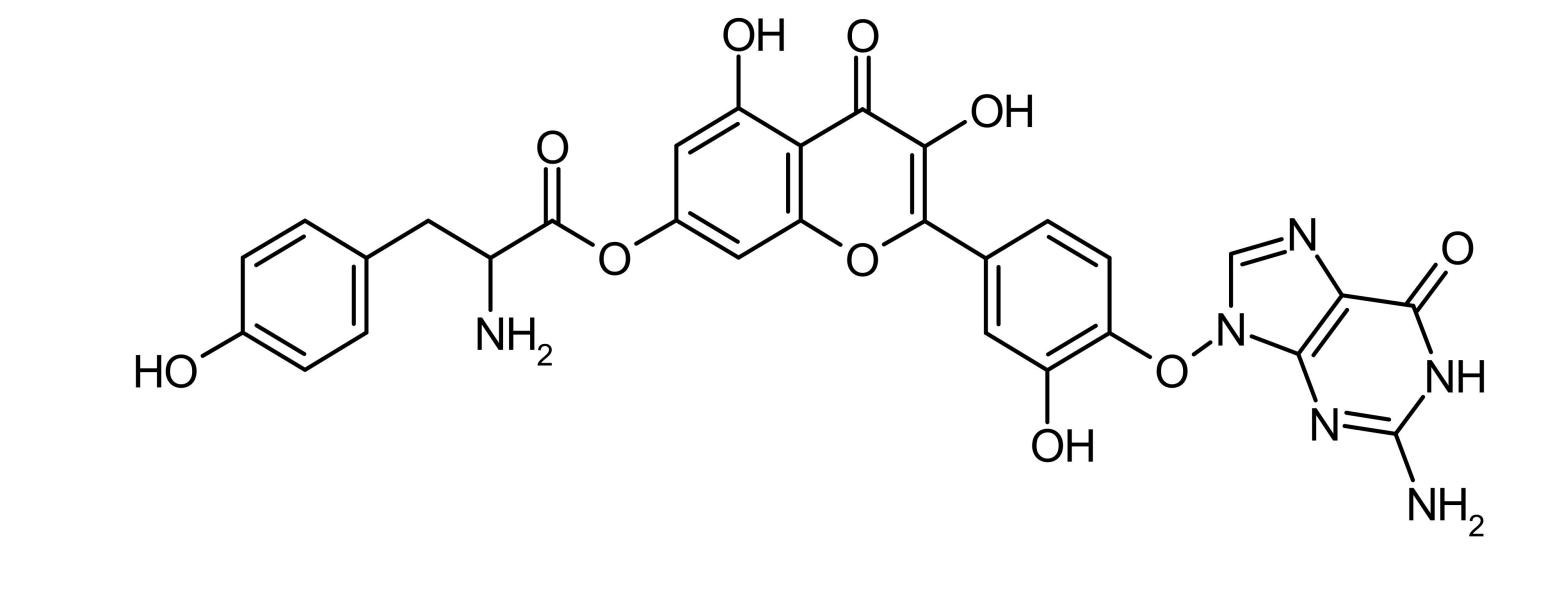
*cardioprotective activity* (regulating the cholesterol synthesis)	*skin protective activity*

**Table 3:** Selected examples of curcumin derivatives with enhanced biological activity.

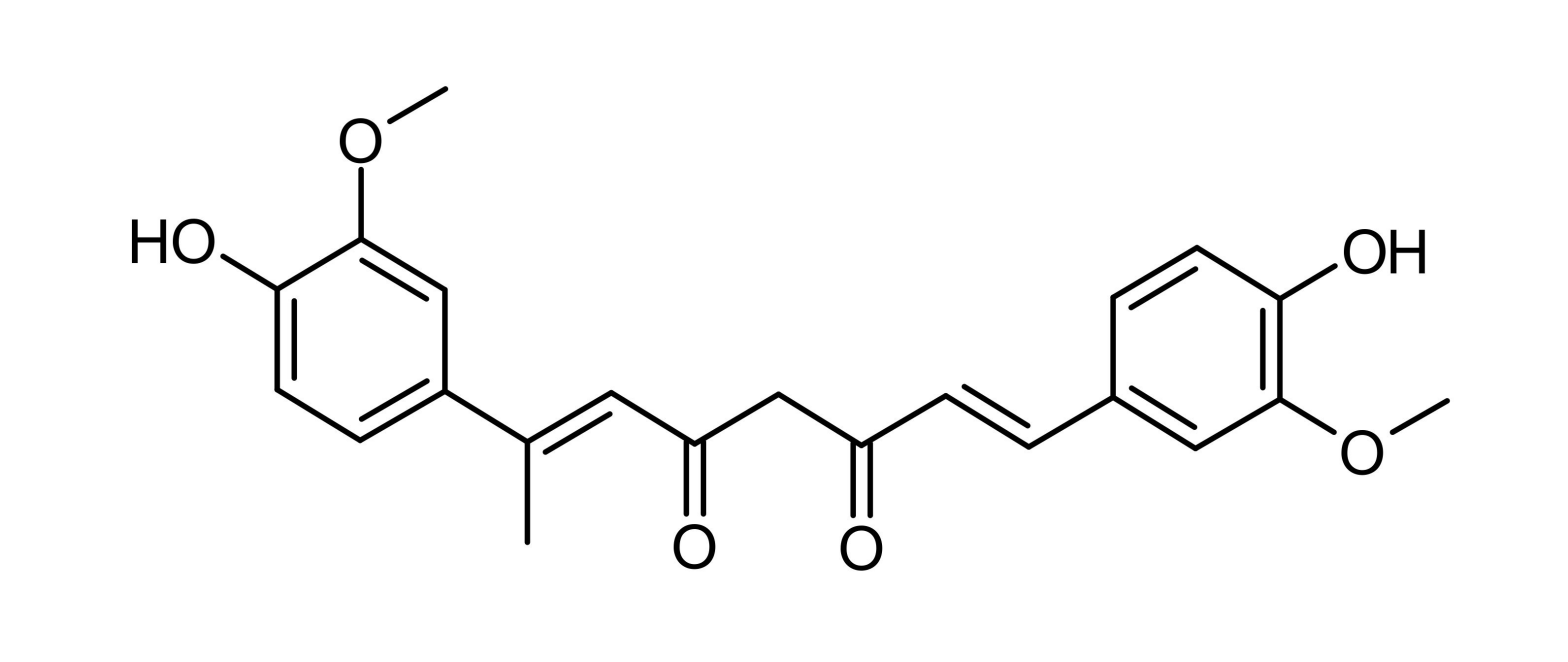	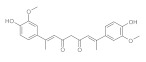
*2-methylcurcumin*	*2,7-dimethylcurcumin*
enhanced antiangiogenesis activity, increased anti-inflammatory activity and stability against enzymatic reduction [410,411,412]	
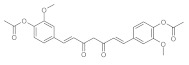	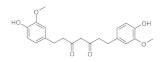
*diacetylcurcumin* antiarthritic activity in mice [413,414]	*tetrahydrocurcumin* increased antioxidant activity [415]
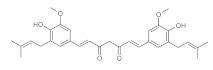	
*diprenylcurcumin* antioxidant properties [416]	
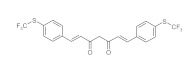	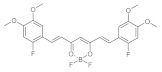
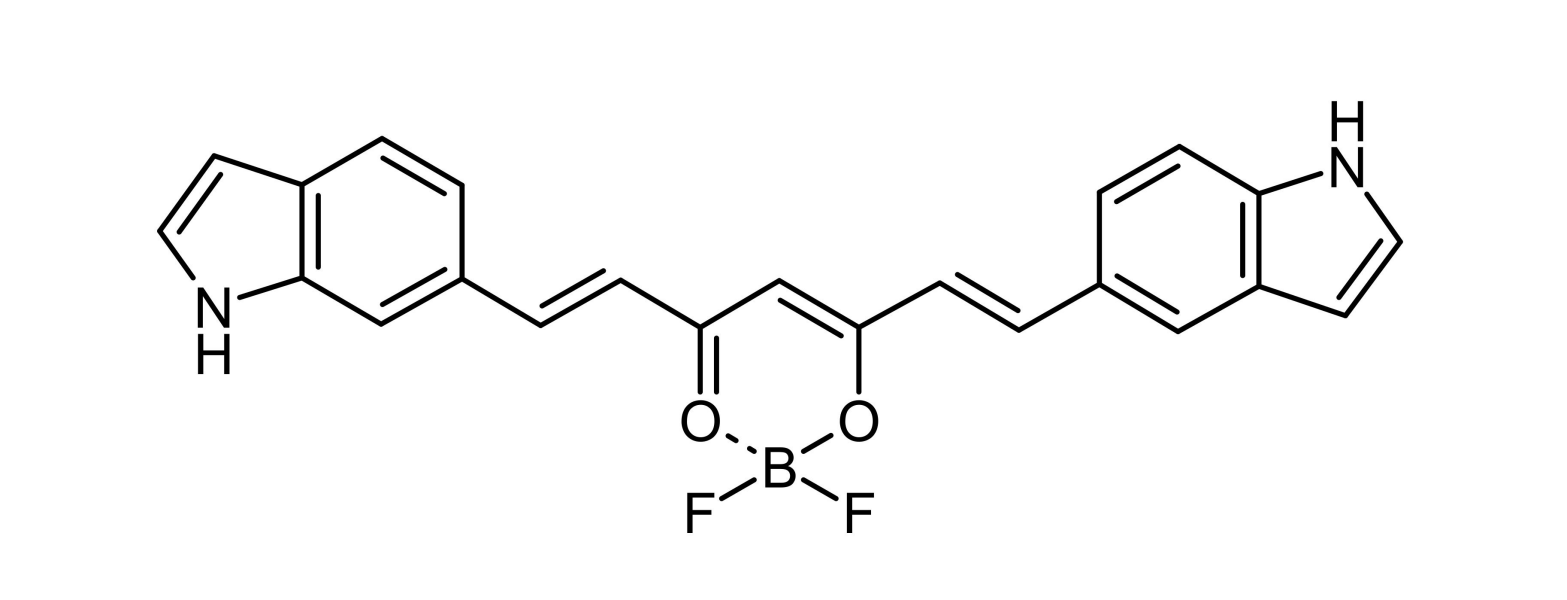	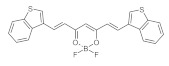
anti-proliferative in vitro bioassay studies [417]	
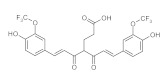	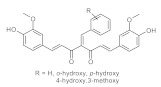
inhibition of TXNIP, protein associated with multiple diseases [418]	*4-benzylidenecurcumins* [419,420]
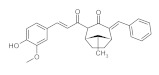	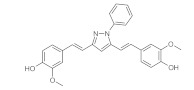
versatile synthesis of 18 dicarbonyl curcumins containing tropane [421]	neuroprotective activity in cell culture; enhances memory; anti-neuroinflammatory effects [422,423,424]
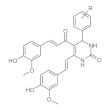	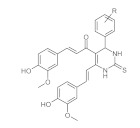
antibacterial and antioxidant activity [425]	
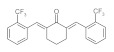	
*(2E,6E)-2,6-bis(2(trifluoromethyl)benzylidene)cyclohexanone* promising for healing diabetic wound in mice [426]	
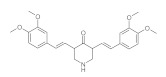	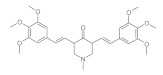
better anticancer activity than curcumin against melanoma cells [427,428]	
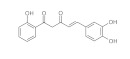	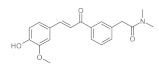
*5-(3,4-dihydroxyphenyl)-3-hydroxy-1-(2-hydroxyphenyl)penta-2,4-dien-1-one* anti-edematogenic/anti-granuloma [429]	effect on hepatic steatosis in mice with induced obesity [430]
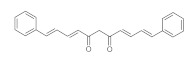	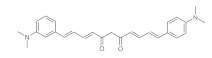
efficient for photodynamic therapy [431]	

## References

[1] Staszowska-Karkut M., Materska M. (2020). Phenolic composition, mineral content, and beneficial bioactivities of leaf extracts from black Currant (*Ribes nigrum* L.), Raspberry (*Rubus idaeus*), and Aronia (*Aronia melanocarpa*). Nutrients.

[2] Metsämuuronen S., Sirén H. (2019). Bioactive phenolic compounds, metabolism and properties: A review on valuable chemical compounds in Scots pine and Norway spruce. Phytochem. Rev..

[3] Mouwakeh A., Kincses A., Nové M., Mosolygó T., Mohácsi-Farkas C., Kiskó G., Spengler G. (2019). *Nigella sativa* essential oil and its bioactive compounds as resistance modifiers against *Staphylococcus aureus*. Phytother. Res..

[4] Chibane L.B., Degraeve P., Ferhout H., Bouajila J., Oulahal N. (2019). Plant Antimicrobial polyphenols as potential natural food preservatives. J. Sci. Food Agric..

[5] Muhammad D.R.A., Tuenter E., Patria G.D., Foubert K., Pieters L., Dewettinck K. (2021). Phytochemical composition and antioxidant activity of *Cinnamomum burmannii* Blume extracts and their potential application in white chocolate. Food Chem..

[6] Pavlić B., Tesli N., Zengin G., Đurović S., Rakić D., Cvetanović A., Gunes A.K., Zeković Z. (2021). Antioxidant and enzyme-inhibitory activity of peppermint extracts and essential oils obtained by conventional and emerging extraction techniques. Food Chem..

[7] Sánchez-Gutiérrez J.A., Moreno-Lorenzana D., Álvarez-Bernal D., Rodríguez-Campos J., Medina-Medrano J.R. (2020). Phenolic profile, antioxidant and anti-proliferative activities of methanolic extracts from *Asclepias linaria* Cav. Leaves. Molecules.

[8] Bodoira R., Maestri D. (2020). Phenolic compounds from nuts: Extraction, chemical profiles, and bioactivity. J. Agric. Food Chem..

[9] Singh B., Singh J.P., Kaur A., Singh N. (2018). Phenolic compounds as beneficial phytochemicals in pomegranate (*Punica granatum* L.) peel: A review. Food Chem..

[10] Servili M., Sordini B., Esposto S., Urbani S., Veneziani G., Di Maio I., Selvaggini R., Taticchi A. (2014). Biological activities of phenolic compounds of extra virgin olive oil. Antioxidants.

[11] Bouyahya A., Chamkhi I., Benali T., Guaouguaou F.-E., Balahbibe A., El Omari N., Taha D., Belmehdi O., Ghokhan Z., El Menyiy N. (2021). Traditional use, phytochemistry, toxicology, and pharmacology of *Origanum majorana* L.. J. Ethnoph..

[12] Mamede L., Ledoux A., Jansen O., Frédérich M. (2020). Natural phenolic compounds and derivatives as potential antimalarial agents. Planta Med..

[13] Mazumder K., Biswas B., Raja I.M., Fukase K. (2020). A review of cytotoxic plants of the indian subcontinent and a broad-spectrum analysis of their bioactive compounds. Molecules.

[14] Mansoori B., Mohammadi A., Doustvandi M.A., Mohammadnejad F., Kamari F., Gjerstorff M.F., Baradaran B., Hamblin M.R. (2019). Photodynamic therapy for cancer: Role of natural products. Photodiag. Photodyn. Ther..

[15] Da Fonsêca D.V., da Silva Maia Bezerra C., Cardoso Lima T., Nóbrega de Almeida R., Pergentino de Sousa D. (2019). Anticonvulsant essential oils and their relationship with oxidative stress in epilepsy. Biomolecules.

[16] Tabassum H., Ahmad A., Ahmad I.Z. (2018). *Nigella sativa* L. and its bioactive constituents as hepatoprotectant: A review. Curr. Pharm. Biotechnol..

[17] Tepe B., Cakir A., Tepe A.S. (2016). Medicinal uses, phytochemistry, and pharmacology of *Origanum onites* (L.): A review. Chem. Biodiv..

[18] Yamada M., Ono K., Hamaguchi T., Noguchi-Shinohara M., Vassallo N. (2015). Natural phenolic compounds as therapeutic and preventive agents for cerebral amyloidosis. Natural Compounds as Therapeutic Agents for Amyloidogenic Diseases.

[19] Gutiérrez-Grijalva E.P., Ambriz-Pére D.L., Leyva-López N., Castillo-López R.I., Heredia J.B. (2016). Review: Dietary phenolic compounds, health benefits and bioaccessibility. Archiv. Latinoam. Nutr..

[20] Kimura H., Ohtsuka K., Matsumoto A., Ougi T., Ishibashi Y., Yamano K. (2015). High performance phenolic resin based on untreated natural herbaceous lignin. Polym. Polym. Compos..

[21] Campos D., Betalleluz I., Tauquino R., Chirinos R., Pedreschi R. (2009). Nutritional and functional characterisation of Andean chicuru (*Stangea rhizanta*). Food Chem..

[22] Lombardo L., Grasso F., Lanciano F., Loria S., Monetti E., Atta-ur-Rahman (2018). Broad-Spectrum health protection of extra virgin olive oil compounds. Studies in Natural Products Chemistry.

[23] Kaushik P., Andújar I., Vilanova S., Plazas M., Gramazio P., Herraiz F.J., Brar N.S., Prohens J. (2015). Breeding vegetables with increased content in bioactive phenolic acids. Molecules.

[24] Dall’Acqua S., Ak G., Sut S., Zengin G., Yıldıztugay E., Mahomoodally M.F., Sinan K.I., Lobine D. (2020). Comprehensive bioactivity and chemical characterization of the endemic plant *Scorzonera hieraciifolia* Hayek extracts: A promising source of bioactive compounds. Food Res. Int..

[25] Zengin G., Mahomoodally M.F., Rocchetti G., Lucini L., Sieniawska E., Świątek Ł., Rajtar B., Polz-Dacewicz M., Senkardes I., Aktumsek A. (2020). Chemical characterization and bioactive properties of different extracts from *Fibigia clypeata*, an unexplored plant food. Foods.

[26] Quílez M., Ferreres F., López-Miranda S., Salazar E., Jordán M.J. (2020). Seed oil from mediterranean aromatic and medicinal plants of the lamiaceae family as a source of bioactive components with nutritional. Antioxidants.

[27] Silva V., Falco V., Dias M.I., Barros L., Silva A., Capita R., Alonso-Calleja C., Amaral J.S., Igrejas G., Ferreira I.C.F.R. (2020). Evaluation of the phenolic profile of *Castanea sativa* mill. by-products and their antioxidant and antimicrobial activity against multiresistant bacteria. Antioxidants.

[28] Golkar P., Moattar F. (2019). Essential oil composition, bioactive compounds, and antioxidant activities in *Iberis amara* L.. Nat. Prod. Commun..

[29] Sut S., Dall’Acqua S., Zengin G., Senkardes I., Bulut G., Cvetanović A., Stupar A., Mandić A., Picot-Allain C., Dogan A. (2019). Influence of different extraction techniques on the chemical profile and biological properties of *Anthemis cotula* L.: Multifunctional aspects for potential pharmaceutical applications. J. Pharm. Biomed. Anal..

[30] Petrović M., Pastor F., Ðurović S., Veljović S., Gorjanović S., Sredojević M., Vukosavljević P. (2021). Evaluation of novel green walnut liqueur as a source of antioxidants: Multi-method approach. J. Food. Sci. Technol..

[31] Walsh D.J., Livinghouse T., Goeres D.M., Mettler M., Stewart P.S. (2019). Antimicrobial activity of naturally occurring phenols and derivatives against biofilm and planktonic bacteria. Front. Chem..

[32] Caleja C., Finimundy T.C., Pereira C., Barros L., Calhelha R.C., Sokovic M., Ivanov M., Carvalho A.M., Rosa E., Ferreira I.C.F.R. (2019). Challenges of traditional herbal teas: Plant infusions and their mixtures with bioactive properties. Food Funct..

[33] Andrade C., Ferreres F., Gomes N.G.M., Duangsrisai S., Srisombat N., Vajrodaya S., Pereira D.M., Gil-Izquierdo A., Andrade P.B., Valentão P. (2019). Phenolic profiling and biological potential of ficus curtipes corner leaves and stem bark: 5-lipoxygenase inhibition and interference with NO levels in LPS-stimulated RAW264.7 macrophages. Biomolecules.

[34] Garzon A.G., Drago S.R. (2018). Free a-amino acids, g-Aminobutyric acid (GABA), phenolic compounds and their relationships with antioxidant properties of sorghum malted in different conditions. J. Food Sci. Technol..

[35] Quintero-Flórez A., Pereira-Caro G., Sánchez-Quezada C., Moreno-Rojas J.M., Gaforio J.J., Jimenez A., Beltran G. (2018). Effect of olive cultivar on bioaccessibility and antioxidant activity of phenolic fraction of virgin olive oil. Eur. J. Nutr..

[36] Uzun Y., Dalar A., Konczak I. (2017). *Sempervivum davisii*: Phytochemical composition, antioxidant and lipase-inhibitory activities. Pharm. Biol..

[37] Atiya A., Sinha B.N., Lal U.R. (2017). Bioactive phenylpropanoid analogues from *Piper betle* L. var. *birkoli* leaves. Nat. Prod. Res..

[38] Freitas dos Santos H., Ferreira Campos J., Miranda dos Santos C., Perrella Balestieri J.B., Brentan Silva D., Carollo C.A., de Picoli Souza K., Estevinho L.M., dos Santos E.L. (2017). Chemical profile and antioxidant, anti-inflammatory, antimutagenic and antimicrobial activities of geopropolis from the stingless bee *Melipona orbignyi*. Int. J. Mol. Sci..

[39] Martini S., Conte A., Tagliazucchi D. (2017). Phenolic compounds profile and antioxidant properties of six sweet cherry (*Prunus avium*) cultivars. Food Res. Int..

[40] Guenes M.E., Şahin S., Demir C., Borum E., Tosunoğlu A. (2016). Determination of phenolic compounds profile in chestnut and floral honeys and their antioxidant and antimicrobial activities. J. Food Biochem..

[41] Herraiz F.J., Villano D., Plazas M., Vilanova S., Ferreres F., Prohens J., Moreno D.A. (2016). Phenolic profile and biological activities of the pepino (*Solanum muricatum*) fruit and its wild relative *S. caripense*. Int. J. Mol. Sci..

[42] Mathew S., Abraham T.E., Zakaria Z.A. (2015). Reactivity of phenolic compounds towards free radicals under in vitro conditions. J. Food Sci. Tech..

[43] Detsi A., Kavetsou E., Kostopoulou I., Pitterou I., Pontillo A.R.N., Tzani A., Christodoulou P., Siliachli A., Zoumpoulakis P. (2020). Nanosystems for the encapsulation of natural products: The case of chitosan biopolymer as a matrix. Pharmaceutics.

[44] Delogu G., Juliano C.C.A., Usai M. (2015). *Thymus catharinae* camarda essential oil: β-cyclodextrin inclusion complexes, evaluation of antimicrobial activity. Nat. Prod. Res..

[45] Dall’Acqua S., Kumar G., Sinan K.I. (2020). An insight into *Cochlospermum planchonii* extracts obtained by traditional and green extraction methods: Relation between chemical compositions and biological properties by multivariate analysis. Ind. Crops Prod..

[46] Nazeam J.A., AL-Shareef W.A., Helmy M.W., El-Haddad A.E. (2020). Bioassay-guided isolation of potential bioactive constituents from pomegranate agrifood by-product. Food Chem..

[47] Borrás Linares I., Arráez-Romána D., Herrero M., Ibáñez E., Segura-Carretero A., Fernández-Gutiérrez A. (2011). Comparison of different extraction procedures for the comprehensive characterization of bioactive phenolic compounds in *Rosmarinus officinalis* by reversed-phase high-performance liquid chromatography with diode array detection coupled to electrospray time-of-flight mass spectrometry. J. Chrom. A.

[48] Alves-Silva J.M., Guerra I., Gonçalves M.J., Cavaleiro C., Cruz M.T., Figueirinha A., Salgueiro L. (2020). Chemical composition of *Crithmum maritimum* L. essential oil and hydrodistillation residual water by GC-MS and HPLC-DAD-MS/MS, and their biological activities. Ind. Crops Prod..

[49] Mahomoodally M.F., Dall’Acqua S., Sinan K.I., Sut S., Ferrarese I., Etienne O.K., Sadeer N.B., Ak G., Zengin G. (2020). Phenolic compounds analysis of three Euphorbia species by LC-DAD-MS*^n^* and their biological properties. J. Pharm. Biomed. Anal..

[50] Ma W., Tang B., Row K.H. (2017). Exploration of a ternary deep eutectic solvent of methyltriphenylphosphonium bromide/chalcone/formic acid for the selective recognition of rutin and quercetin in Herba *Artemisiae scopariae*. J. Sep. Sci..

[51] Arun K.P., Brindha P. (2015). Investigations into phenolic and alkaloid constituents of *Jatropha tanjorensis* by LC-MS/MS and evaluating its bioactive property. Asian J. Chem..

[52] Oldoni T.L.C., Melo P.S., Massarioli A.P., Moreno I.A.M., Bezerra R.M.N., Rosalen P.L., da Silva G.V.J., Nascimento A.M., Alencar S.M. (2016). Bioassay-guided isolation of proanthocyanidins with antioxidant activity from peanut (*Arachis hypogaea*) skin by combination of chromatography techniques. Food Chem..

[53] Alagawany M., Farag M.R., Salah A.A., Mahmoud M.A. (2020). The role of oregano herb and its derivatives as immunomodulators in fish. Rev. Aquacult..

[54] Sorrenti V., Fortinguerra S., Caudullo G., Buriani A. (2020). Deciphering the role of polyphenols in sports performance: From nutritional genomics to the gut microbiota toward phytonutritional epigenomics. Nutrients.

[55] Araghi M., Moslehi Z., Nafchi A.A., Mostahsan A., Salamat N., Garmakhany A.D. (2015). Cold water fish gelatin modification by a natural phenolic cross-linker (ferulic acid and caffeic acid). Food Sci. Nutr..

[56] Zhang X., Do M.D., Casey P., Sulistio A., Qiao G.G., Lundin L., Lillford P., Kosaraju S. (2010). Chemical modification of gelatin by a natural phenolic cross-linker, tannic acid. J. Agric. Food Chem..

[57] Alirezalua K., Pateiro M., Yaghoubi M., Alirezalu A., Peighambardoust S.H., Lorenzo J.M. (2020). Phytochemical constituents, advanced extraction technologies and technofunctional properties of selected Mediterranean plants for use in meat products. A comprehensive review. Trends Food Sci. Technol..

[58] Gullón P., Astray G., Gullón B., Tomasevic I., Lorenzo J.M. (2020). Pomegranate peel as suitable source of high-added value bioactives: Tailored functionalized meat products. Molecules.

[59] Mainente F., Menin A., Alberton A., Zoccatelli G., Rizzi C. (2018). Evaluation of the sensory and physical properties of meat and fish derivatives containing grape pomace powders International. J. Food Sci. Tech..

[60] Gutiérrez-del-Río I., Fernández J., Lombó F. (2018). Plant nutraceuticals as antimicrobial agents in food preservation: Terpenoids, polyphenols and thiols. Int. J. Antimicr. Agents.

[61] Akl E.M., Dacrory S., Abdel-Aziz M.S., Kamel S., Fahim A.M. (2021). Preparation and characterization of novel antibacterial blended films based on modified carboxymethyl cellulose/phenolic compounds. Polym. Bull..

[62] Ibrahim S.A., Soliman O.R. (2016). Nano-encapsulation of bioactive oils through emulsion polymerization as antimicrobials and its adhesion to packaging films. Der Pharm. Lett..

[63] Jing S., Li T., Li X., Xu Q., Hu J., Li R. (2014). Phenolic foams modified by cardanol through bisphenol modification. J. Appl. Polym. Sci..

[64] Mahendran A.R., Wuzella G., Aust N., Mueller U., Kandelbauer A. (2013). Processing and characterization of natural fibre reinforced composites using lignin phenolic binder. Polym. Polym. Comp..

[65] Rahim M.A., Kristufek S.L., Pan S., Richardson J.J., Caruso F. (2019). Phenolic building blocks for the assembly of functional materials. Angew. Chem. Int. Ed..

[66] Lomonaco D., Maia F.J.N., Clemente C.S., Mota J.P.F., Costa A.E., Mazzetto S.E. (2012). Thermal studies of new biodiesel antioxidants synthesized from a natural occurring phenolic lipid. Fuel.

[67] Morales J.C., Lucas R., Preedy V.R., Watson R.R. (2010). Structure-activity relationship of phenolic antioxidants and olive components. Olives and Olive Oil in Health and Disease Prevention.

[68] Chen Y., Xiao H., Zheng J., Liang G. (2015). Structure-thermodynamics-antioxidant activity relationships of selected natural phenolic acids and derivatives: An experimental and theoretical evaluation. PLoS ONE.

[69] Zeljković S.C., Topčagic A., Požgan F., Štefane B., Tarkowski P., Maksimović M. (2015). Antioxidant activity of natural and modified phenolic extracts from *Satureja montana* L.. Ind. Crops Prod..

[70] Sun Q., Heilmann J., König B. (2015). Natural phenolic metabolites with anti-angiogenic properties—A review from the chemical point of view. Beilstein J. Org. Chem..

[71] Groussin A.-L., Antoniotti S. (2012). Valuable chemicals by the enzymatic modification of molecules of natural origin: Terpenoids, steroids, phenolics and related compounds. Biores. Technol..

[72] Cravens A., Payne J., Smolke C.D. (2019). Synthetic biology strategies for microbial biosynthesis of plant natural products. Nat. Commun..

[73] Zielińska-Błajet M., Feder-Kubis J. (2020). Monoterpenes and their derivatives—Recent development in biological and medical applications. Int. J. Mol. Sci..

[74] Pawełczyk A., Olender D., Sowa-Kasprzak K., Zaprutko L. (2020). Linked drug-drug conjugates based on triterpene and phenol structures. Rational synthesis, molecular properties, toxicity and bioactivity prediction. Arab. J. Chem..

[75] Nesterkina M., Kravchenko I. (2017). Synthesis and pharmacological properties of novel esters based on monoterpenoids and glycine. Pharmaceuticals.

[76] Rajput J.D., Bagul S.D., Pete U.D., Zade C.M., Padhye S.B., Bendre R.S. (2018). Perspectives on medicinal properties of natural phenolic monoterpenoids and their hybrids. Mol. Divers..

[77] Mastelić J., Jerković I., Blažević I., Poljak-Blaži M., Borović S., Ivanćić-Baće I., Smrećki V., Žarković N., Brćić-Kostic K., Vikić-Topić D. (2008). Comparative study on the antioxidant and biological activities of carvacrol, thymol, and eugenol derivatives. J. Agric. Food Chem..

[78] Schreiner L., Bauer J., Ortner E., Buettner A. (2020). Structure−Odor activity studies on derivatives of aromatic and oxygenated monoterpenoids synthesized by modifying *p*-cymene. J. Nat. Prod..

[79] Marković A.K., Torić J., Barbarić M., Brala C.J. (2019). Hydroxytyrosol, tyrosol and derivatives and their potential effects on human health. Molecules.

[80] Zang H., Shen P., Xu Q., Zhang L., Xia G., Sun J., Zhu J., Yang X. (2019). Synthesis and biological activities of tyrosol phenolic acid ester derivatives. Chem. Nat. Compd..

[81] Barontini M., Bernini R., Carastro I., Gentili P., Romani A. (2014). Synthesis and DPPH radical scavenging activity of novel compounds obtained from tyrosol and cinnamic acid derivatives. New J. Chem..

[82] Lombrea A., Antal D., Ardelean F., Avram S., Pavel I.Z., Vlaia L., Mut A.-M., Diaconeasa Z., Dehelean C.A., Soica C. (2020). A recent insight regarding the phytochemistry and bioactivity of *Origanum vulgare* L. essential oil. Int. J. Mol. Sci..

[83] Aljaafari M.N., AlAli A.O., Baqais L., Alqubaisy M., AlAli M., Molouki A., Ong-Abdullah J., Abushelaibi A., Lai K.-S., Erin Lim S.-H. (2021). An overview of the potential therapeutic applications of essential oils. Molecules.

[84] Nabavi S., Marchese A., Izadi M., Curti V., Daglia M., Nabavi S.F. (2015). Plants belonging to the genus *Thymus* as antibacterial agents: From farm to pharmacy. Food Chem..

[85] Marinelli L., Di Stefano A., Cacciatore I. (2018). Carvacrol and its derivatives as antibacterial agents. Phytochem. Rev..

[86] De Mesquita B.M., do Nascimento P.G.G., Souza L.G.S., de Farias I.F., da Silva R.A.C., de Lemos T.L.G., Monte F.J.Q., Oliveira I.R., Trevisan M.T.S., da Silva H.C. (2018). Synthesis, larvicidal and acetylcholinesterase ihibitory activities of carvacrol/thymol and derivatives. Quim. Nova.

[87] Damasceno S.R.B., Oliveira F.R.A.M., Carvalho N.S., Brito C.F.C., Silva I.S., Sousa F.B.M., Silva R.O., Sousa D.P., Barbosa A.L.R., Freitas R.M. (2014). Carvacryl acetate, a derivative of carvacrol, reduces nociceptive and inflammatory response in mice. Life Sci..

[88] Pires L.F., Muratori Costa L., Cardoso de Almeida A.A., Almeida Silva O., Santos Cerqueira G., de Sousa D.P., de Freitas R.M. (2014). Is there a correlation between in vitro antioxidant potential and in vivo effect of carvacryl acetate against oxidative stress in mice hippocampus?. Neurochem. Res..

[89] Wang K., Jiang S., Pu T., Fan L., Su F., Ye M. (2019). Antifungal activity of phenolic monoterpenes and structure-related compounds against plant pathogenic fungi. Nat. Prod. Res..

[90] Pires L.F., Muratori Costa L., Almeida Silva O., Cardoso de Almeida A.A., Cerqueira G.S., de Sousa D.P., de Freitas R.M. (2013). Anxiolytic-like effects of carvacryl acetate, a derivative of carvacrol in mice. Pharmacol. Biochem. Behav..

[91] Novato T., Gomes G.A., Zeringóta V., Franco C.T., de Oliveira D.R., Melo D., de Carvalho M.G., Daemond E., de Oliveira Monteiroc C.M. (2018). In vitro assessment of the acaricidal activity of carvacrol, thymol, eugenol and their acetylated derivatives on *Rhipicephalus microplus* (Acari: Ixodidae). Vet. Parasitol..

[92] Konig I.F.M., Gonçalves R.R.P., Oliveira M.V.S., Silva C.M., Thomasi S.S., Peconick A.P., Remedio R.N. (2019). Sublethal concentrations of acetylcarvacrol strongly impact oocyte development of engorged female cattle ticks *Rhipicephalus microplus* (Canestrini, 1888) (Acari: Ixodidae). Ticks Tick Borne Dis..

[93] De Santana M.T., Barros Silva V., de Brito R.G., dos Santos P.L., de Holanda Cavalcanti S.C., Oliveira Barreto E., de Souza Ferro J.N., Viana dos Santos M.R., de Sousa Araújo A.A., Quintans-Júnior L.J. (2014). Synthesis and pharmacological evaluation of carvacrol propionate. Inflammation.

[94] Nesterkina M., Kravchenko I. (2016). Synthesis and pharmacological properties of novel esters based on monocyclic terpenes and GABA. Pharmaceuticals.

[95] Nesterkina M., Kravchenko I. (2016). Analgesic activity of novel GABA esters after transdermal delivery. Nat. Prod. Commun..

[96] Ashraf Z., Rafiq M., Nadeem H., Hassan M., Afzal S., Waseem M., Afzal K., Latip J. (2017). Carvacrol derivatives as mushroom tyrosinase inhibitors; synthesis, kinetics mechanism and molecular docking studies. PLoS ONE.

[97] Mathela C.S., Singh K.K., Gupta V.K. (2010). Synthesis and in vitro antibacterial activity of thymol and carvacrol derivatives. Acta Pol. Pharm..

[98] Alokam R., Jeankumar V.U., Sridevi J.P., Matikonda S.S., Peddi S., Alvala M., Yogeeswari P., Sriram D. (2014). Identification and structure–activity relationship study of carvacrol derivatives as *Mycobacterium tuberculosis* chorismate mutase inhibitors. J. Enzyme Inhib. Med. Chem..

[99] Wang K., Jiang S., Yang Y., Fan L., Su F., Ye M. (2019). Synthesis and antifungal activity of carvacrol and thymol esters with heteroaromatic carboxylic acids. Nat. Prod. Res..

[100] Walsh D.J., Livinghouse T., Durling G.M., Chase-Bayless Y., Arnold A.D., Stewart P.S. (2020). Sulfenate esters of simple phenols exhibit enhanced activity against biofilms. ACS Omega.

[101] Pinheiro P.F., Parreira Menini L.A., Campos Bernardes P., Henriques Saraiva S., Mesquita Carneiro J.W., Vidal Costa A., Rodrigues Arruda T., Ribeiro Lage M., Martins Gonçalves P., de Oliveira Bernardes C. (2018). Semisynthetic phenol derivatives obtained from natural phenols: Antimicrobial activity and molecular properties. J. Agric. Food Chem..

[102] Marinelli L., Fornasari E., Eusepi P., Ciulla M., Genovese S., Epifano F., Fiorito S., Turkez H., Ortücü S., Mingoia M. (2019). Carvacrol prodrugs as novel antimicrobial agents. Eur. J. Med. Chem..

[103] Cacciatore I., Di Giulio M., Fornasari E., Di Stefano A., Cerasa L.S., Marinelli L., Turkez H., Di Campli E., Di Bartolomeo S., Robuffo I. (2015). Carvacrol codrugs: A new approach in the antimicrobial plan. PLoS ONE.

[104] Gharbi A., Legigan T., Humblot V., Papot S., Berjeaud J.-M. (2015). Surface functionalization by covalent immobilization of an innovative carvacrol derivative to void fungal biofilm formation. AMB Express.

[105] Bonfim R.R., Paiva-Souza I.O., Moraes J.P., Pereira D.S., Santos C.A., Santana D.G., Thomazzi S.M., Ferro J.N.S., Barreto E.O., Sousa D.P. (2014). Isopropoxy-carvacrol, a derivative obtained from carvacrol, reduces acute inflammation and nociception in rodents. Basic Clin. Pharmacol..

[106] Patil J.U., Suryawanshi K.C., Patil P.B., Chaudhary S.R., Pawar N.S. (2010). Synthesis and antibacterial activity of carvacryl ethers. J. Asian Nat. Prod. Res..

[107] Sisto F., Carradori S., Guglielmi P., Traversi C.B., Spano M., Sobolev A.P., Secci D., Di Marcantonio M.C., Haloci E., Grande R. (2020). Synthesis and biological evaluation of carvacrol-based derivatives as dual inhibitors of *H. pylori* strains and ags cell proliferation. Pharmaceuticals.

[108] Bkhaitan M.M., Alarjah M., Mirza A.Z., Abdalla A.N., El-Said H.M., Faidah H.S. (2018). Preparation and biological evaluation of metronidazole derivatives with monoterpenes and eugenol. Chem. Biol. Drug Des..

[109] Nesterkina M., Bilokon S., Alieksieieva T., Chebotar S., Kravchenko I. (2020). Toxic effect and genotoxicity of carvacrol ethers in *Drosophila melanogaster*. Mutat. Res. Fund. Mol. Mech. Mutagen..

[110] Brotzman N., Xu Y., Graybill A., Cocolas A., Ressler A., Seeram N.P., Ma H., Henry G.E. (2019). Synthesis and tyrosinase inhibitory activities of 4-oxobutanoate derivatives of carvacrol and thymol. Bioorg. Med. Chem. Lett..

[111] Uddin A., Singh V., Irfan I., Mohammad T., Singh Hada R., Hassan M.I., Abid M., Singh S. (2020). Identification and structure–activity relationship (SAR) studies of carvacrol derivatives as potential anti-malarial against *Plasmodium falciparum* falcipain-2 protease. Bioorg. Chem..

[112] Aneja B., Azam M., Alam S., Perwez A., Maguire R., Yadava U., Kavanagh K., Daniliuc C.G., Rizvi M.M.A., Haq Q.M.R. (2018). Natural product-based 1,2,3-triazole/sulfonate analogues as potential chemotherapeutic agents for bacterial infections. ACS Omega.

[113] Bytyqi-Damoni A., Kestane A., Taslimi P., Tuzun B., Zengin M., Bilgicli H.G., Gulcin İ. (2020). Novel carvacrol based new oxypropanolamine derivatives: Design, synthesis, characterization, biological evaluation, and molecular docking studies. J. Mol. Struct..

[114] Kurt B.Z., Gazioglu I., Dag A., Salmas R.E., Kayık G., Durdagi S., Sonmez F. (2017). Synthesis, anticholinesterase activity and molecular modeling study of novel carbamate-substituted thymol/carvacrol derivative. Bioorg. Med. Chem..

[115] Zengin Kurt B., Durdagi S., Celebi G., Ekhteiari Salmas R., Sonmez F. (2020). Synthesis, anticholinesterase activity and molecular modeling studies of novel carvacrol substituted amide derivatives. J. Biomol. Struct. Dyn..

[116] Bassanetti I., Carcelli M., Buschini A., Montalbano S., Leonardi G., Pelagatti P., Tosi G., Massi P., Fiorentini L., Rogolino D. (2017). Investigation of antibacterial activity of new classes of essential oils derivatives. Food Control.

[117] Chen G.-Q., Sun D., Yang J.-M., Zhang S., Tian Y.-E., Che Z.-P., Liu S.-M., Jiang J., Lin X.-M. (2021). Synthesis of sulfonate derivatives of carvacrol and thymol as anti-oomycetes agents. J. Asian Nat. Prod. Res..

[118] Rajput J.D., Bagul S.D., Hosamani A.A., Patil M.A., Bendre R.S. (2017). Synthesis, characterizations, biological activities and docking studies of novel dihydroxy derivatives of natural phenolic monoterpenoids containing azomethine linkage. Res. Chem. Intermed..

[119] Rajput J.D., Bagul S.D., Bendre R.S. (2017). Design, synthesis, biological screenings and docking simulations of novel carvacrol and thymol derivatives containing acetohydrazone linkage. Res. Chem. Intermed..

[120] Rajput J.D., Bagul S.D., Bendre R.S. (2017). Synthesis, biological activities and molecular docking simulation of hydrazone scaffolds of carvacrol, thymol and eugenol. Res. Chem. Intermed..

[121] Bagul S.D., Rajput J.D., Tadavi S.K., Bendre R.S. (2017). Design, synthesis and biological activities of novel 5-isopropyl-2-methylphenolhydrazide-based sulfonamide derivatives. Res. Chem. Intermed..

[122] Sobotta L., Lijewski S., Dlugaszewska J., Nowicka J., Mielcarek J., Goslinski T. (2019). Photodynamic inactivation of *Enterococcus faecalis* by conjugates of zinc(II) phthalocyanines with thymol and carvacrol loaded into lipid vesicles. Inorg. Chim. Acta.

[123] Marchese A., Orhan I.E., Daglia M., Barbieri R., Di Lorenzo A., Nabavi S.F., Gortzi O., Izadi M., Nabavi S.M. (2016). Antibacterial and antifungal activities of thymol: A brief review of the literature. Food Chem..

[124] Kumar A., Singh S.P., Chhokar S.S. (2008). Thymol and its derivatives as antimicrobial agents. Nat. Prod. Commun..

[125] Talavera-Alemán A., Rodríguez-García G., López Y., García-Gutiérrez H.A., Torres-Valencia J.M., del Río R.E., Cerda-García-Rojas C.M., Joseph-Nathan P., Gómez-Hurtado M.A. (2016). Systematic evaluation of thymol derivatives possessing stereogenic or prostereogenic centers. Phytochem. Rev..

[126] De Silvestro I., Drew S.L., Nichol G.S., Duarte F., Lawrence A.L. (2017). Total synthesis of a dimeric thymol derivative isolated from *Arnica sachalinensis*. Angew. Chem. Int. Ed..

[127] El-Miligy M.M.M., Hazzaa A.A., El-Zemity S.R., Al-Kubeisi A.K. (2019). Synthesis of thymol derivatives as potential non-irritant antimicrobial and insecticidal agents. Curr. Bioact. Comp..

[128] Cherkasov R.A., Nizamov I.S., Gabdullina G.T., Almetkina L.A., Shamilov R.R., Sofronov A.V. (2013). Dithiophosphoric and dithiophosphonic acids and their derivatives on the basis of thymol: Synthesis and antimicrobial activity. Phosphorus Sulfur.

[129] Matela G., Aman R., Sharma C., Chaudhray S. (2013). Reactions of tin and triorganotin(IV) isopropoxides with thymol derivative: Synthesis, characterization and in vitro antimicrobial screening. J. Serb. Chem. Soc..

[130] Robledo S., Osorio E., Munõz D., Jaramillo L.M., Restrepo A., Arango G., Vélez I. (2005). In vitro and in vivo cytotoxicities and antileishmanial activities of thymol and hemisynthetic derivatives. Antimicrob. Agents Chemother..

[131] More D.H., Pawar N.S., Dewang P.M., Patil S.L., Mahulikar P.P. (2004). Microwave-assisted Sinthesis of thymyl ethers and esters in aqueous medium. Russ. J. Gen. Chem..

[132] Sabour A., El Asbahani A., Bentahar S., Ait taleb M., Lacherai A., Jilale A. (2019). Synthesis of some thymol derivatives for enhanced antibacterial activity. Mor. J. Chem..

[133] Barros Silva V., Lima Travassos D., Nepel A., Barison A., Vilaça Costa E., Scotti L., Scotti M.T., Bezerra Mendonça-Junior F.J., La Corte dos Santos R., Cabral de Holanda Cavalcanti S. (2017). Synthesis and chemometrics of thymol and carvacrol derivatives as larvicides against *Aedes aegypti*. J. Arthropod-Borne Dis..

[134] De Morais S.M., Vila-Nova N.S., Bevilaqua C.M.L., Rondon F.C., Lobo C.H., Noronha Moura A.d.A.A., Sales A.D., Ribeiro Rodrigues A.P., de Figuereido J.R., Campello C.C. (2014). Thymol and eugenol derivatives as potential antileishmanial agents. Bioorg. Med. Chem..

[135] André W.P.P., Cavalcante G.S., Correia Ribeiro W.L., dos Santos J.M.L., Freitas Macedo I.T., de Paula H.C.B., de Morais S.M., de Melo J.V., Leal Bevilaqua C.M. (2017). Anthelmintic effect of thymol and thymol acetate on sheep gastrointestinal nematodes and their toxicity in mice. Braz. J. Vet. Parasitol..

[136] Xavier F.J.S., Rodrigues K.A.d.F., De Oliveira R.G., Lima Junior C.G., Da Câmara Rocha J., Keesen T.S.L., De Oliveira M.R., Silva F.P.L., Vasconcellos M.L.A.d.A. (2016). Synthesis and in vitro anti *Leishmania amazonensis* biological screening of morita-baylis-hillman adducts prepared from eugenol, thymol and carvacrol. Molecules.

[137] Hassib S.T., Hassan G.S., El-Zaher A.A., Fouad M.A., Abd El-Ghafar O.A., Taha E.A. (2019). Synthesis and biological evaluation of new prodrugs of etodolac and tolfenamic acid with reduced ulcerogenic potential. Eur. J. Pharm. Sci..

[138] Sawraj S., Bhardawaj T.R., Sharma P.D. (2012). Design, synthesis, and evaluation of novel indomethacin-antioxidant codrugs as gastrosparing NSAIDs. Med. Chem. Res..

[139] Sehajpal S., Prasad D.N., Singh R.K. (2018). Synthesis and evaluation of prodrugs of ketoprofen with antioxidants as gastroprotective NSAIDs. Asian J. Chem..

[140] Dhaneshwar S., Patel V., Patil D., Meena G. (2013). Studies on synthesis, stability, release and pharmacodynamic profile of a novel diacerein-thymol prodrug. Bioorg. Med. Chem. Lett..

[141] Ashraf Z., Rafiq M., Seo S.-Y., Kwon K.S., Babar M.M., Sadaf Zaidi N.U.S. (2015). Kinetic and in silico studies of novel hydroxy-based thymol analogues as inhibitors of mushroom tyrosinase. Eur. J. Med. Chem..

[142] Kang H.H., Rho H.S., Hwang J.S., Oh S.-G. (2003). Depigmenting activity and low cytotoxicity of alkoxy benzoates or alkoxy cinnamate in cultured melanocytes. Chem. Pharm. Bull..

[143] Hwan Lee J., Lee E.-S., Bae H., Hwang J.-A., Kim S.-H., Kim D.-Y., Park N.-H., Rho H.S., Kim Y.J., Oh S.-G. (2017). Antimelanogenic efficacy of melasolv (3,4,5-Trimethoxycinnamate Thymol Ester) in melanocytes and three-dimensional human skin equivalent. Ski. Pharmacol. Physiol..

[144] Sheng Z., Ge S., Xu X., Zhang Y., Wu P., Zhang K., Xu X., Li C., Zhao D., Tang X. (2018). Design, synthesis and evaluation of cinnamic acid ester derivatives as mushroom tyrosinase inhibitors. Med. Chem. Commun..

[145] Zengin M., Genca H., Taslimi P., Kestane A., Guclu E., Ogutlu A., Karabay O., Gulçin I. (2018). Novel thymol bearing oxypropanolamine derivatives as potent some metabolic enzyme inhibitors—Their antidiabetic, anticholinergic and antibacterial potentials. Bioorg. Chem..

[146] Kumar T.V.S., Sankar K.-U., Divakar S. (2013). Synthesis of thymol glycosides under SCCO_2_ conditions using amyloglucosidase from *Rhizopus* mold. J. Food Sci. Technol..

[147] Epps S.V.R., Harvey R.B., Byrd J.A., Petrujkić B.T., Sedej I., Beier R.C., Phillips T.D., Hume M.E., Anderson R.C., Nisbet D.J. (2015). Comparative effect of thymol or its glucose conjugate, thymol-β-D-glucopyranoside, on *Campylobacter* in avian gut contents. J. Environ. Sci. Health B.

[148] James Bound D., Bettadaiah B.K., Srinivas P. (2014). ZnBr_2_-Catalyzed and Microwave-Assisted Synthesis of 2,3-Unsaturated Glucosides of Hindered Phenols and Alcohols. Synth. Commun..

[149] Bound J.S., Murthy P.S., Srinivas P. (2016). 2,3-Dideoxyglucosides of selected terpene phenols and alcohols as potent antifungal compounds. Food Chem..

[150] Havasi M.H., Ressler A.J., Parks E.L., Cocolas A.H., Weaver A., Seeram N.P., Henry G.E. (2020). Antioxidant and tyrosinase docking studies of heterocyclic sulfide derivatives containing a thymol moiety. Inorg. Chim. Acta.

[151] Piombino C., Lange H., Sabuzi F., Galloni P., Conte V., Crestini C. (2020). lignosulfonate microcapsules for delivery and controlled release of thymol and derivatives. Molecules.

[152] Swain S.S., Paidesetty S.K., Padhy R.N. (2017). Antibacterial activity, computational analysis and host toxicity study of thymol-sulfonamide conjugates. Biomed. Pharmacother..

[153] Sathe P.S., Rajput J.D., Gunaga S.S., Patel H.M., Bendre R.S. (2019). Synthesis, characterization, and antioxidant activity of thymol-based paracetamol analogues. Res. Chem. Intermed..

[154] Swain S.S., Paidesetty S.K., Padhy R.N. (2019). Synthesis of novel thymol derivatives against MRSA and ESBL producing pathogenic bacteria. Nat. Prod. Res..

[155] Radwan M.A., El-Zemity S.R., Mohamed S.A., Sherby S.M. (2008). Potential of some monoterpenoids and their new *N*-methylcarbamate derivatives against Schistosomiasis snail vector, *Biomphalaria alexandrina*. Ecotoxicol. Environ. Saf..

[156] El-Zemity S.R., Radwan M.A., El-Monam Mohamed S.A., Sherby S.M. (2008). Antibacterial screening of some essential oils, monoterpenoids and novel *N*-methyl carbamates based on monoterpenoids against *Agrobacterium tumefaciens* and *Erwinia carotovora*. Arch. Phytopathol. Plant Prot..

[157] Latacz G., Lubelska A., Jastrzębska-Więsek M., Partyka A., Marć M.A., Satała G., Wilczyńska D., Kotańska M., Więcek M., Kamińska K. (2019). The 1,3,5-triazine derivatives as innovative chemical family of 5-ht6 serotonin receptor agents with therapeutic perspectives for cognitive impairment. Int. J. Mol. Sci..

[158] Nagle P., Pawar Y., Sonawane A., Bhosale S., More D. (2014). Docking simulation, synthesis and biological evaluation of novel pyridazinone containing thymol as potential antimicrobial agents. Med. Chem. Res..

[159] Nagle P.S., Pawar Y.A., Sonawane A.E., Bhosale S.M., More D.H. (2012). Synthesis and evaluation of antioxidant and antimicrobial properties of thymol containing pyridone moieties. Med. Chem. Res..

[160] Kulabaş N., Tatar E., Özakpınar Ö.B., Özsavcı D., Pannecouque C., De Clercq E., Küçükgüzel I. (2016). Synthesis and antiproliferative evaluation of novel 2-(4*H*-1,2,4-triazole-3-ylthio)acetamide derivatives as inducers of apoptosis in cancer cells. Eur. J. Med. Chem..

[161] Inci Gul H., Yamali C., Tugce Yasa A., Unluer E., Sakagami H., Tanc M., Supuran C.T. (2016). Carbonic anhydrase inhibition and cytotoxicity studies of Mannich base derivatives of thymol. J. Enzym. Inhib. Med. Chem..

[162] Raghuvanshi D.S., Verma N., Singh S.V., Khare S., Pal A., Negi A.S. (2019). Synthesis of thymol-based pyrazolines: An effort to perceive novel potent antimalarials. Bioorg. Chem..

[163] Kaur R., Darokar M.P., Chattopadhyay S.K., Krishna V., Ahmad A. (2014). Synthesis of halogenated derivatives of thymol and their antimicrobial activities. Med. Chem. Res..

[164] Getrey L., Krieg T., Hollmann F., Schrader J., Holtmann D. (2014). Enzymatic halogenation of the phenolic monoterpenes thymol and carvacrol with chloroperoxidase. Green Chem..

[165] Sabuzi F., Churakova E., Galloni P., Wever R., Hollmann F., Floris B., Conte V. (2015). Thymol bromination—A comparison between enzymatic and chemical catalysis. Eur. J. Inorg. Chem..

[166] Floris B., Sabuzi F., Coletti A., Conte V. (2017). Sustainable vanadium-catalyzed oxidation of organic substrates with H_2_O_2_. Cat. Today.

[167] Sabuzi F., Pomarico G., Floris B., Valentini F., Galloni P., Conte V. (2019). Sustainable bromination of organic compounds: A critical review. Coord. Chem. Rev..

[168] Galloni P., Conte V., Sabuzi F., Migliore L., Thaller M.C., Matteucci G. (2018). Sustainable process for the preparation of highly pure 4-bromothymol and its application as antimicrobial agent. World Patent.

[169] Ulanowska M., Olas B. (2021). Biological properties and prospects for the application of eugenol-A review. Int. J. Mol. Sci..

[170] Modjinou T., Versace D.-L., Abbad-Andallousi S., Bousserrhine N., Dubot P., Langlois V., Renard E. (2016). Antibacterial and antioxidant bio-based networks derived from eugenol using photo-activated thiol-ene reaction. React. Funct. Polym..

[171] Da Silva F.F.M., Monte F.J.Q., de Lemos T.L.G., do Nascimento P.G.G., de Medeiros Costa A.K., de Paiva L.M.M. (2018). Eugenol derivatives: Synthesis, characterization, and evaluation of antibacterial and antioxidant activities. Chem. Cent. J..

[172] Charan Raja M.R., Velappan A.B., Chellappan D., Debnath J., Mahapatra S.K. (2017). Eugenol derived immunomodulatory molecules against visceral leishmaniasis. Eur. J. Med. Chem..

[173] Rahim N.H.C.A., Asari A., Ismail N., Osman H. (2017). Synthesis and antibacterial study of eugenol derivatives. Asian J. Chem..

[174] Behrouz S., Rad M.N.S., Taghavi Shahraki B., Fathalipour M., Behrouz M., Mirkhani H. (2019). Design, synthesis, and in silico studies of novel eugenyloxy propanol azole derivatives having potent antinociceptive activity and evaluation of their β-adrenoceptor blocking property. Mol. Divers..

[175] Fernandes M.J.G., Pereira R.B., Pereira D.M., Fortes A.G., Castanheira E.M.S., Gonçalves M.S.T. (2020). New eugenol derivatives with enhanced insecticidal activity. Int. J. Mol. Sci..

[176] Lenardão E.J., Jacob R.G., Mesquita K.D., Lara R.G., Webber R., Martinez D.M., Savegnago L., Mendes S.R., Alvesa D., Perin G. (2012). Glycerol as a promoting and recyclable medium for catalyst-free synthesis of linear thioethers: New antioxidants from eugenol. Green Chem. Lett. Rev..

[177] Carrasco H., Raimondi M., Svetaz L., Di Liberto M., Rodriguez M.V., Espinoza L., Madrid A., Zacchino S. (2012). Antifungal activity of eugenol analogues. influence of different substituents and studies on mechanism of action. Molecules.

[178] Rudyanto M., Ekowati J., Widiandani T., Honda T. (2014). Synthesis and brine shrimp lethality test of some benzoxazine and aminomethyl derivatives of eugenol. Int. J. Pharm. Pharm. Sci..

[179] De Carvalho L.I.S., Alvarenga D.J., do Carmo L.C.F., de Oliveira L.G., Silva N.C., Dias A.L.T., Coelho L.F.L., de Souza T.B., Dias D.F., Carvalho D.T. (2017). Antifungal activity of new eugenol-benzoxazole hybrids against *Candida* spp.. J. Chem..

[180] Olea A., Bravo A., Martínez R., Thomas M., Sedan C., Espinoza L., Zambrano E., Carvajal D., Silva-Moreno E., Carrasco H. (2019). Antifungal activity of eugenol derivatives against *Botrytis cinerea*. Molecules.

[181] Gul F., Mohammed Khan K., Adhikari A., Zafar S., Akram M., Khan H., Saeed M. (2016). Antimicrobial and antioxidant activities of a new metabolite from *Quercus incana*. Nat. Prod. Res..

[182] Anjum N.F., Purohit M.N., Yogish Kumar H., Ramya K., Javid S., Salahuddin M.D., Prashantha Kumar B.R. (2020). Semisynthetic derivatives of eugenol and their biological properties: A fleeting look at the promising molecules. J. Biol. Act. Prod. Nat..

[183] Kaufman T.S. (2015). The multiple faces of eugenol. A versatile starting material and building block for organic and bio-organic synthesis and a convenient precursor toward bio-based fine chemicals. J. Braz. Chem. Soc..

[184] Espinoza-Hicks J.C., Zaragoza-Galán G., Chávez-Flores D., Ramos-Sánchez V.H., Tamariz J., Camacho-Dávila A.A. (2018). A convergent total synthesis of the biologically active benzofurans ailanthoidol, egonol and homoegonol from biomass-derived eugenol. Synthesis.

[185] Kamiloglu A.A., Direkel S., Yalazan H., Kantekin H., Acar I. (2020). Octa- and tetra-substituted phthalocyanines with methoxyeugenol group: Synthesis, characterization and in vitro antimicrobial activity. J. Coord. Chem..

[186] Nguyen Thi Thanh C., Truong Thi Cam M., Pham Van T., Nguyen L., Nguyen Ha M., Van Meervelt L. (2017). Synthesis, structure and in vitro cytotoxicity of platinum(II) complexes containing eugenol and a quinolin-8-ol-derived chelator. Acta Cryst..

[187] Naddeo M., Vigliotta G., Pellecchia C., Pappalardo D. (2020). Synthesis of biobased polymacrolactones with pendant eugenol moieties as novel antimicrobial thermoplastic materials. React. Funct. Polym..

[188] Maurya A.K., Agarwal K., Gupta A.C., Saxena A., Nooreen Z., Tandon S., Ahmad A., Bawankule D.U. (2020). Synthesis of eugenol derivatives and its antiinflammatory activity against skin inflammation. Nat. Prod. Res..

[189] D’ Avila Farias M., Oliveira P.S., Pereira Dutra F.S., Jacobsen Fernandes T., de Pereira C.M.P., Quintana de Oliveira S., Moro Stefanello F., Leonetti Lencina C., Gatto Barschak A. (2014). Eugenol derivatives as potential anti-oxidants: Is phenolic hydroxyl necessary to obtain an effect?. J. Pharm. Pharmacol..

[190] Mastelari Martins R., D’Avila Farias M., Nedel F., de Pereira C.M.P., Lencina C., Guerra Lund R. (2016). Antimicrobial and cytotoxic evaluation of eugenol derivatives. Med. Chem. Res..

[191] Makuch E., Nowak A., Günther A., Pełech R., Kucharski L., Duchnik W., Klimowicz A. (2020). Enhancement of the antioxidant and skin permeation properties of eugenol by the esterifcation of eugenol to new derivatives. AMB Expr..

[192] Lone S.A., Wani M.Y., Fru P., Ahmad A. (2020). Cellular apoptosis and necrosis as therapeutic targets for novel eugenol tosylate congeners against *Candida albicans*. Sci. Rep..

[193] Lone S.H., Ahmad A. (2020). Inhibitory effect of novel eugenol tosylate congeners on pathogenicity of *Candida albicans*. BMC Complement. Med. Ther..

[194] Ahmad A., Wani M.Y., Khan A., Manzoor N., Molepo J. (2015). Synergistic interactions of eugenol-tosylate and its congeners with fluconazole against *Candida albicans*. PLoS ONE.

[195] Li J.-Y., Yu Y.-G., Wang Q.-W., Zhang J.-Y., Yang Y.-J., Li B., Zhou X.-Z., Niu J.-R., Wei X.-J., Liu X.-W. (2012). Synthesis of aspirin eugenol ester and its biological activity. Med. Chem. Res..

[196] Ma N., Liu X.-W., Kong X.-J., Li S.-H., Jiao Z.-H., Qin Z., Yang Y.-J., Li J.-Y. (2018). Aspirin eugenol ester regulates cecal contents metabolomic profile and microbiota in an animal model of hyperlipidemia. BMC Vet. Res..

[197] Shen D.S., Yang Y.-J., Kong X.-J., Ma N., Liu X.-W., Li S.-H., Jiao Z.H., Qin Z., Huang M.-Z., Li J.-Y. (2019). Eugenol ester inhibits agonist-induced platelet aggregation in vitro by regulating PI3K/Akt, MAPK and Sirt 1/CD40L pathways. Eur. J. Pharmacol..

[198] Zhang Z.-D., Yang Y.-J., Liu X.-W., Qin Z., Li S.-H., Li J.-Y. (2020). The protective effect of aspirin eugenol ester on paraquat-induced acute liver injury rats. Front. Med..

[199] Redasani V.K., Bari S.B. (2012). Synthesis and evaluation of mutual prodrugs of ibuprofen with menthol, thymol, and eugenol. Eur. J. Med. Chem..

[200] Topal F., Gulcin I., Dastan A., Guney M. (2017). Novel eugenol derivatives: Potent acetylcholinesterase and carbonic anhydrase inhibitors. Int. J. Biol. Macromol..

[201] Bilgiçli H.G., Ergön D., Taslimi P., Tüzün B., Kuru I.A., Zengin M., Gülçin I. (2020). Novel propanolamine derivatives attached to 2-*metoxifenol* moiety: Synthesis, characterization, biological properties, and molecular docking studies. Bioorg. Chem..

[202] Bilgiçli H.G., Kestane A., Taslimi P., Karabay O., Bytyqi-Damoni A., Zengin M., Gulçin I. (2019). Novel eugenol bearing oxypropanolamines: Synthesis, characterization, antibacterial, antidiabetic, and anticholinergic potentials. Bioorg. Chem..

[203] Taia A., Essaber M., Oubella A., Aatif A., Bodiguel J., Jamart-Gregoire B., Itto M.Y.A., Morjani H. (2020). Synthesis, characterization, and biological evaluation of new heterocyclic systems 1, 2, 3-triazole-isoxazoline from eugenol by the mixed condensation reactions. Synth. Commun..

[204] De Oliveira A.S., Gazolla P.A.R., Oliveira A.F.C.d.S., Pereira W.L., Viol L.C.d.S., Maia A.F.d.S., Santos E.G., da Silva I.E.P., de Oliveira Mendes T.A., da Silva A.M. (2019). Discovery of novel West Nile Virus protease inhibitor based on isobenzonafuranone and triazolic derivatives of eugenol and indan-1,3-dione scaffolds. PLoS ONE.

[205] Teixeira R.R., Rodrigues Gazolla P.A., da Silva A.M., Gonçalves Borsodi M.P., Rossi Bergmann B., Salgado Ferreira R., Vaz B.G., Vasconcelos G.A., Lima W.P. (2018). Synthesis and leishmanicidal activity of eugenol derivatives bearing 1,2,3-triazole functionalities. Eur. J. Med. Chem..

[206] De Souza T.B., Caldas I.S., Paula F.R., Rodrigues C.C., Carvalho D.T., Dias D.F. (2020). Synthesis, activity, and molecular modeling studies of 1,2,3-triazole derivatives from natural phenylpropanoids as new trypanocidal agents. Chem. Biol. Drug Des..

[207] Dos Santos T., Coelho C.M., Elias T.C., Siqueira F.S., Nora E.S.S.D., de Campos M.M.A., de Souza G.A.P., Coelho L.F.L., Carvalho D.T. (2019). Synthesis and biological evaluation of new eugenol-derived 1,2,3-triazoles as antimycobacterial agents. J. Braz. Chem. Soc..

[208] De Souza T.B., Raimundo P.O.B., Andrade D.F., Hipólito T.M.M., Silva N.C., Dias A.L.T., Ikegaki M., Rocha R.P., Coelho L.F.L., Veloso M.P. (2015). Synthesis and antimicrobial activity of 6-triazolo-6-deoxy eugenol glucosides. Carbohyd. Res..

[209] Rohane S.H., Chauhan A.J., Fuloria N.K., Fuloria S. (2020). Synthesis and in vitro antimycobacterial potential of novel hydrazones of eugenol. Arab. J. Chem..

[210] Hipólito T.M.M., Bastos G.T.L., Barbosa T.W.L., de Souza T.B., Coelho L.F.L., Dias A.L.T., Rodríguez I.C., dos Santos M.H., Ferreira Dias D., Franco L.L. (2018). Synthesis, activity and docking studies of eugenol-based glucosides as new agents against *Candida* sp.. Chem. Biol. Drug Des..

[211] Braga Resende D., de Abreu Martins H.H., de Souza T.B., Carvalho D.T., Hilsdorf Piccoli R., Schwan R.F., Dias D.R. (2017). Synthesis and sp. evaluation of peracetyl and deacetyl glycosides of eugenol, isoeugenol and dihydroeugenol acting against food contaminating bacteria. Food Chem..

[212] De Souza T.B., Orlandi M., Coelho L.F.L., Malaquias L.C.C., Dias A.L.T., de Carvalho R.R., Silva N.C., Carvalho D.T. (2014). Synthesis and in vitro evaluation of antifungal and cytotoxic activities of eugenol glycosides. Med. Chem. Res..

[213] De Souza T.B., de Oliveira Brito K.M., Silva N.C., Rocha R.P., de Sousa G.F., Duarte L.P., Coelho L.F.L., Dias A.L.T., Veloso M.P., Carvalho D.T. (2016). New eugenol glucoside-based derivative shows fungistatic and fungicidal activity against opportunistic *Candida glabrata*. Chem. Biol. Drug Des..

[214] Fernández-Mar M.I., Mateos R., García-Parrilla M.C., Puertas B., Cantos-Villar E. (2012). Bioactive compounds in wine: Resveratrol, hydroxytyrosol and melatonin: A review. Food Chem..

[215] Mattio L.M., Catinella G., Dallavalle S., Pinto A. (2020). Stilbenoids: A natural arsenal against bacterial pathogens. Antibiotics.

[216] Mattio L.M., Catinella G., Pinto A., Dallavalle S. (2020). Natural and nature-inspired stilbenoids as antiviral agents. Eur. J. Med. Chem..

[217] Deng L.-J., Qi M., Li N., Lei Y.-H., Zhang D.-M., Chen J.-X. (2020). Natural products and their derivatives: Promising modulators of tumor immunotherapy. J. Leukoc. Biol..

[218] Ashrafizadeh M., Rafiei H., Mohammadinejad R., Farkhondeh T., Samarghandian S. (2021). Anti-tumor activity of resveratrol against gastric cancer: A review of recent advances with an emphasis on molecular pathways. Cancer Cell Int..

[219] Ferraz da Costa D.C., Rangel L.P., da Cunha Martins-Dinis M.M.D., da Silva Ferretti G.D., Ferreira V.F., Silva J.L. (2020). Anticancer potential of resveratrol, b-lapachone and their analogues. Molecules.

[220] Li K.-X., Ji M.-J., Sun H.-J. (2021). An updated pharmacological insight of resveratrol in the treatment of diabetic nephropathy. Gene.

[221] Lin M.-H., Hung C.-F., Sung H.-C., Yang S.-C., Yu H.-P., Fang J.-Y. (2021). The bioactivities of resveratrol and its naturally occurring derivatives on skin. Food Drug Anal..

[222] Shaito A., Posadino A.M., Younes N., Hasan H., Halabi S., Alhababi D., Al-Mohannadi A., Abdel-Rahman W.M., Eid A.H., Nasrallah G.K. (2020). Potential adverse effects of resveratrol: A literature review. J. Mol. Sci..

[223] Intagliata S., Modica M.N., Santagati L.M., Montenegro L. (2019). Strategies to improve resveratrol systemic and topical bioavailability: An update. Antioxidants.

[224] Vervandier-Fasseur D., Vang O., Latruffe N. (2017). Special Issue: Improvements for resveratrol efficacy. Molecules.

[225] Nawaz W., Zhou Z., Deng S., Ma X., Ma X., Li C., Shu X. (2017). Therapeutic versatility of resveratrol derivatives. Nutrients.

[226] Singh D., Mendonsa R., Koli M., Subramanian M., Nayak S.K. (2019). Antibacterial activity of resveratrol structural analogues: A mechanistic evaluation of the structure-activity relationship. Toxicol. Appl. Pharmacol..

[227] Pecyna P., Wargula J., Murias M., Kucinska M. (2020). More than resveratrol: New insights into stilbene-based compounds. Biomolecules.

[228] Biasutto L., Mattarei A., Azzolini M., La Spina M., Sassi N., Romio M., Paradisi C., Zoratti M. (2017). Resveratrol derivatives as a pharmacological tool. Ann. N. Y. Acad. Sci.

[229] Latruffe N., Vervandier-Fasseur D. (2018). Strategic syntheses of vine and wine resveratrol derivatives to explore their effects on cell functions and dysfunctions. Diseases.

[230] Peñalver P., Belmonte-Reche E., Adán N., Caro M., Mateos-Martín M.L., Delgado M., González-Rey E., Morales J.C. (2018). Alkylated resveratrol prodrugs and metabolites as potential therapeutics for neurodegenerative diseases. Eur. J. Med. Chem..

[231] Yang M.-F., Yao X., Chen L.-M., Gu J.-Y., Yang Z.-H., Chen H.-F., Zheng X., Zheng Z.-T. (2020). Synthesis and biological evaluation of resveratrol derivatives with anti-breast cancer activity. Arch. Pharmazie.

[232] Ahmadi R., Ebrahimzadeh M.A. (2020). Resveratrol—A comprehensive review of recent advances in anticancer drug design and development. Eur. J. Med. Chem..

[233] Venkateswarlu S., Ramachandra M.S., Sethuramu K., Subbaraju V. (2002). Synthesis and antioxidant activity of hispolon, a yellow pigment from *Inonotus hispidius*. Ind. J. Chem..

[234] Khan M.F., Aktar S., Bin Rashid R., Rashid M.A. (2017). In silico investigation of physicochemical, pharmacokinetic and toxicological properties of hispolon. Pharma Chem..

[235] Sarfraz A., Rasul A., Sarfraz I., Shah M.A., Hussain G., Shafiq N., Masood M., Adem S., Sarker S.D., Li X. (2020). Hispolon: A natural polyphenol and emerging cancer killer by multiple cellular signaling pathways. Environ. Res..

[236] Paul M., Panda M.K., Thatoi H. (2019). Developing Hispolon based novel anticancer therapeutics against human (NF-κβ) using in silico approach of modelling, docking and protein dynamics. J. Biomol. Struct. Dyn..

[237] Rossi M., Caruso F., Costanzini I., Kloer C., Sulovari A., Monti E., Gariboldi M., Marras E., Balaji N.V., Ramani M.V. (2019). X-ray crystal structures, density functional theory and docking on deacetylase enzyme for antiproliferative activity of hispolon derivatives on HCT116 colon cancer. Bioorg. Med. Chem..

[238] Fan H.-C., Hsieh Y.-C., Li L.-H., Chang C.-C., Janoušková K., Ramani M.V., Subbaraju G.V., Cheng K.-T., Chang C.-C. (2020). Dehydroxyhispolon methyl ether, a hispolon derivative, inhibits WNT/-catenin signaling to elicit human colorectal carcinoma cell apoptosis. Int. J. Mol. Sci..

[239] Balaji N.V., Ramani M.V., Viana A.G., Sanglard L.P., White J., Mulabagal V., Lee C., Gana T.J., Egiebor N.O., Subbaraju G.V. (2015). Design, synthesis and in vitro cell-based evaluation of the anti-cancer activities of hispolon analogs. Bioorg. Med. Chem..

[240] Balaji N.V., Babu B.H., Subbaraju G.V., Nagasree K.P., Kumar M.M.K. (2017). Synthesis, screening and docking analysis of hispolon analogs as potential antitubercular agents. Bioorg. Med. Chem. Lett..

[241] Chethna P., Iyer S.S., Gandhi V.V., Kunwar A., Singh B.G., Barik A., Balaji N.V., Ramani M.V., Subbaraju G.V., Priyadarsini K.I. (2018). Toxicity and antigenotoxic effect of hispolon derivatives: Role of structure in modulating cellular redox state and thioredoxin reductase. ACS Omega.

[242] Shaikh S.A.M., Barik A., Singh B.G., Madukuri R.V., Balaji N.V., Subbaraju G.V., Naik D.B., Priyadarsini K.I. (2016). Free radical reactions of isoxazole and pyrazole derivatives of hispolon: Kinetics correlated with molecular descriptors. Free Radical Res..

[243] Wei X., Yang Y., Ge J., Lin X., Liu D., Wang S., Zhang J., Zhou G., Li S. (2020). Synthesis, characterization, DNA/BSA interactions and in vitro cytotoxicity study of palladium(II) complexes of hispolon derivatives. J. Inorg. Biochem..

[244] Bertelli M., Kiani A.K., Paolacci S., Manara E., Baglivo M., Kurti D., Dhuli K., Bushati V., Miertus J., Pangallo D. (2020). Hydroxytyrosol: A natural compound with promising pharmacological activities. J. Biotechnol..

[245] Wei J., Wang S., Pei D., Qu L., Li Y., Chen J., Di D., Gao K. (2018). Antibacterial activity of hydroxytyrosol acetate from olive leaves (*Olea Europaea* L.). Nat. Prod. Res..

[246] Yao F., Yang G., Xian Y., Wang G., Zheng Z., Jin Z., Xie Y., Wang W., Gue J., Lin R. (2019). Protective effect of hydroxytyrosol acetate against inflammation of vascular endothelial cells partly through SIRT6-mediated PKM2 signaling pathway. Food Funct..

[247] Qin C., Hu S., Zhang S., Zhao D., Wang Y., Li L., Peng Y., Shi L., Xu X., Wang C. (2021). Hydroxytyrosol acetate improves the cognitive function of APP/PS1 transgenic mice in ERβ-dependent manner. Mol. Nutr. Food Res..

[248] Aparicio-Soto M., Sánchez-Fidalgo S., González-Benjumea A., Maya I., Fernández-Bolaños J.G., Alarcón-de-la-Lastra C. (2015). Naturally occurring hydroxytyrosol derivatives: Hydroxytyrosyl acetate and 3,4-dihydroxyphenylglycol modulate inflammatory response in murine peritoneal macrophages. Potential utility as new dietary supplements. J. Agric. Food Chem..

[249] Martin D., Garcia-Serrano A., Casado V., Vázquez L., Reglero G., Torres C.F. (2014). Antioxidant activity of phosphatidyl derivatives of hydroxytyrosol in edible oils. Eur. J. Lipid Sci. Technol..

[250] Martin D., Moran-Valero M.I., Casado V., Reglero G., Torres C.F. (2014). Phosphatidyl derivative of hydroxytyrosol. In Vitro intestinal digestion, bioaccessibility, and its effect on antioxidant activity. J. Agric. Food Chem..

[251] Montoya T., Aparicio-Soto M., Castejón M.L., Rosillo M.A., Sánchez-Hidalgo M., Begines P., Fernández-Bolaños J.G., Alarcón-de-la-Lastra C. (2018). Peracetylated hydroxytyrosol, a new hydroxytyrosol derivate, attenuates LPS-induced inflammatory response in murine peritoneal macrophages via regulation of non-canonical inflammasome, Nrf2/HO1 and JAK/STAT signaling pathways. J. Nutr. Biochem..

[252] Rodríguez-Gutiérrez G., Rubio-Senent F., Gómez-Carretero A., Maya I., Fernández-Bolaños J., Duthie G.G., de Roos B. (2019). Selenium and sulphur derivatives of hydroxytyrosol: Inhibition of lipid peroxidation in liver microsomes of vitamin E-deficient rats. Eur. J. Nutr..

[253] Majhi S., Das D. (2021). Chemical derivatization of natural products: Semisynthesis and pharmacological aspects—A decade update. Tetrahedron.

[254] Tofani D., Balducci V., Gasperi T., Incerpi S., Gambacorta A. (2010). Fatty acid hydroxytyrosyl esters: Structure/antioxidant activity relationship by ABTS and in cell-culture dcf assays. J. Agric. Food Chem..

[255] Zhou D.-A., Sun Y.-X., Shahidi F. (2017). Preparation and antioxidant activity of tyrosol and hydroxytyrosol esters. J. Funct. Food..

[256] Balducci V., Incerpi S., Stano P., Tofani D. (2018). Antioxidant activity of hydroxytyrosyl esters studied in liposome models. BBA Biomembr..

[257] Lopes R., Costa M., Ferreira M., Gameiro P., Paiva-Martins F. (2021). A new family of hydroxytyrosol phenolipids for the antioxidant protection of liposomal systems. BBA Biomembr..

[258] Candiracci M., Madrona A., Espartero J.L., Zappia G., Piatti E. (2016). Lipophilic hydroxytyrosol esters significantly improve the oxidative state of human red blood cells. J. Funct. Food..

[259] Belmonte-Reche E., Martínez-García M., Peñalver P., Gómez-Pérez V., Lucas R., Gamarro F., Pérez-Victoria J.M., Morales J.C. (2016). Tyrosol and hydroxytyrosol derivatives as antitrypanosomal and antileishmanial agents. Eur. J. Med. Chem..

[260] Funakohi-Tago M., Sakata T., Fujiwara S., Sakakura A., Sugai T., Tago K., Tamura H. (2018). Hydroxytyrosol butyrate inhibits 6-OHDA-induced apoptosis through activation of the Nrf2/HO-1 axis in SH-SY5Y cells. Eur. J. Pharmacol..

[261] Xie Y., Xu Y., Chen Z., Lu W., Li N., Wang Q., Shao L., Li Y., Yang G., Bian X. (2017). A new multifunctional hydroxytyrosol-fenofibrate with antidiabetic, antihyperlipidemic, antioxidant and antiinflammatory action. Biomed. Pharmacother..

[262] Xie Y.-D., Chen Z.-Z., Li N., Lu W.-F., Xu Y.-H., Lin Y.Y., Shao L.-H., Wang Q.-T., Guo L.-Y., Gao Y.-Q. (2018). Hydroxytyrosol nicotinate, a new multifunctional hypolipidemic and hypoglycemic agent. Biomed. Pharmacother..

[263] Crisante F., Taresco V., Donelli G., Vuotto C., Martinelli A., D’Ilario L., Pietrelli L., Francolini I., Piozzi A., Doninelli G. (2016). Antioxidant hydroxytyrosol-based polyacrylate with antimicrobial and antiadhesive activity versus *Staphylococcus epidermidis*. Advances in Microbiology, Infectious Diseases and Public Health.

[264] Romanucci V., Giordano M., De Tommaso G., Iuliano M., Bernini R., Clemente M., Garcia-Viñuales S., Milardi D., Zarrelli A., Di Fabio G. (2021). Synthesis of new tyrosol-based phosphodiester derivatives: Effect on amyloid β aggregation and metal chelation ability. Chem. Med. Chem..

[265] Marrero A.D., Castilla L., Espartero J.L., Madrona A., Quesada A.R., Medina M.A., Martínez-Poveda B. (2020). A comparative study of the antiangiogenic activity of hydroxytyrosyl alkyl ethers. Food Chem..

[266] Muñoz-Marín J., De La Cruz J.P., Reyes J.J., López-Villodres J.A., Guerrero A., López-Leiva I., Espartero J.L., Labajos M.T., González-Correa J.A. (2013). Hydroxytyrosyl alkyl ether derivatives inhibit platelet activation after oral administration to rats. Food Chem. Toxicol..

[267] Pereira-Caro G., Mateos R., Traka M.H., Bacon J.R., Bongaerts R., Sarriá B., Bravo L., Kroon P.A. (2013). Hydroxytyrosyl ethyl ether exhibits stronger intestinal anticarcinogenic potency and effects on transcript profiles compared to hydroxytyrosol. Food Chem..

[268] Cert R., Madrona A., Espartero J.L., Pérez-Camino M.C. (2015). Antioxidant activity of alkyl hydroxytyrosyl ethers in unsaturated lipids. Food Funct..

[269] Nieto-Domínguez M., de Eugenio L.I., Peñalver P., Belmonte-Reche E., Morales J.C., Poveda A., Jiménez-Barbero J., Prieto A., Plou F.J., Martínez M.J. (2017). Enzymatic synthesis of a novel neuroprotective hydroxytyrosyl glycoside. J. Agric. Food Chem..

[270] Trujillo M., Gallardo E., Madrona A., Bravo L., Sarriá B., González-Correa J.A., Mateos R., Espartero J.L. (2014). Synthesis and antioxidant activity of nitrohydroxytyrosol and its acyl derivatives. J. Agric. Food Chem..

[271] Gallardo E., Palma-Valdés R., Sarriá B., Gallardo I., de la Cruz J.P., Bravo L., Mateos R., Espartero J.L. (2016). Synthesis and antioxidant activity of Alkyl nitroderivatives of hydroxytyrosol. Molecules.

[272] Fernandez-Pastor I., Fernandez-Hernandez A., Rivas F., Martinez A., Garcia-Granados A., Parra A. (2016). Synthesis and antioxidant activity of hydroxytyrosol Alkyl-carbonate derivatives. J. Nat. Prod..

[273] Fernandez-Pastor I., Martínez-García M., Medina-O’Donnell M., Rivas F., Martinez A., Pérez-Victoria J.M., Parra A. (2018). Semisynthesis of ω-hydroxyalkylcarbonate derivatives of hydroxytyrosol as antitrypanosome agents. J. Nat. Prod..

[274] Servili M., Esposto S., Fabiani R., Urbani S., Taticchi A., Mariucci F., Selvaggini R., Montedoro G.F. (2009). Phenolic compounds in olive oil: Antioxidant, health and organoleptic activities according to their chemical structure. Inflammopharmacology.

[275] Khadem S., Marles R.J. (2010). Monocyclic phenolic acids; hydroxy- and polyhydroxybenzoic acids: Occurrence and recent bioactivity studies. Molecules.

[276] Heleno S.A., Martins A., Queiroz M.J.R.P., Ferreira I.C.F.R. (2015). Bioactivity of phenolic acids: Metabolites versus parent compounds: A review. Food Chem..

[277] Muronetz V.I., Barinova K., Kudryavtseva S., Medvedeva M., Melnikova A., Sevostyanova I., Semenyuk P., Stroylova Y., Sova M. (2020). Natural and synthetic derivatives of hydroxycinnamic acid modulating the pathological transformation of amyloidogenic proteins. Molecules.

[278] Kumar N., Goel N. (2019). Phenolic acids: Natural versatile molecules with promising therapeutic applications. Biotechnol. Reports.

[279] Manuja R., Sachdeva S., Jain A., Chaudhary J. (2013). A comprehensive review on biological activities of p-hydroxy benzoic acid and its derivatives. Int. J. Pharm. Sci. Rev. Res..

[280] Vinayagam R., Jayachandran M., Xu B. (2016). Antidiabetic effects of simple phenolic acids: A comprehensive review. Phytother. Res..

[281] Kakkar S., Bais S. (2014). A review on protocatechuic acid and its pharmacological potential. ISRN Pharmacol..

[282] Khan A.K., Rashid R., Nighat F., Sadaf M., Mir S., Khan S., Jabeen N., Murtaza G. (2015). Pharmacological activities of protocatechuic acid. Acta Pol. Pharm. Drug Res..

[283] Al Zahrani N.A., El-Shishtawy R.M., Asiri A.M. (2020). Recent developments of gallic acid derivatives and their hybrids in medicinal chemistry: A review. Eur. J. Med. Chem..

[284] Naira N., Asdaq S.M.B., Heba S., Said A.H.E.l.A. (2016). Gallic acid: A promising lead molecule for drug development. J. App. Pharm..

[285] Ou S., Kwok K.-C. (2004). Ferulic acid: Pharmaceutical functions, preparation and applications in foods. J. Sci. Food Agric..

[286] Zhao Z., Moghadasian M.H. (2008). Chemistry, natural sources, dietary intake and pharmacokinetic properties of ferulic acid: A review. Food Chem..

[287] Brenelli de Paiva L., Goldbeck R., Dantas dos Santos W., Squina F.M. (2013). Ferulic acid and derivatives: Molecules with potential application in the pharmaceutical field. Braz. J. Pharm. Sci..

[288] Pei K., Ou J., Huang C., Ou S. (2015). Derivatives of ferulic acid: Structure, preparation and biological activities. Ann. Res. Rev. Biol..

[289] Zduńska K., Dana A., Kolodziejczak A., Rotsztejn H. (2018). Antioxidant properties of ferulic acid and its possible application. Skin Pharmacol. Physiol..

[290] Jiang R.-W., Lau K.-M., Hon P.-M., Mak T.C.W., Woo K.-S., Fung K.-P. (2005). Chemistry and biological activities of caffeic acid derivatives from *Salvia miltiorrhiza*. Curr. Med. Chem..

[291] Zhang P., Tang Y., Li N.-G., Zhu Y., Duan J.-A. (2014). Bioactivity and chemical synthesis of caffeic acid phenethyl ester and its derivatives. Molecules.

[292] Chen T., Zhu G., Meng X., Zhang X. (2020). Recent developments of small molecules with anti-inflammatory activities for the treatment of acute lung injury. Eur. J. Med. Chem..

[293] Wang J., Mahajani M., Jackson S.L., Yang Y., Chen M., Ferreira E.M., Lin Y., Yan Y. (2017). Engineering a bacterial platform for total biosynthesis of caffeic acid derived phenethyl esters and amides. Metab. Eng..

[294] Widjaja A., Yeh T.-H., Ju Y.-H. (2008). Enzymatic synthesis of caffeic acid phenethyl ester. J. Chin. Inst. Chem. Eng..

[295] Chen H.-C., Chen J.-H., Chang C., Shieh C.-J. (2011). Optimization of ultrasound-accelerated synthesis of enzymatic caffeic acid phenethyl ester by response surface methodology. Ultrasonics Sonochem..

[296] Kurata A., Kitamura Y., Irie S., Takemoto S., Akai Y., Hirota Y., Fujita T., Iwai K., Furusawa M., Kishimoto N. (2010). Enzymatic synthesis of caffeic acid phenethyl ester analogues in ionic liquid. J. Biotechnol..

[297] De Cássia Orlandi Sardi J., Gullo F.P., Almeida Freires I., de Souza Pitangui N., Segalla M.P., Fusco-Almeida A.M., Rosalen P.L., Regasini L.O., Soares Mendes-Giannini M.J. (2016). Synthesis, antifungal activity of caffeic acid derivative esters, and their synergism with fluconazole and nystatin against *Candida* spp.. Diagn. Microbiol. Infect. Dis..

[298] Da Cunha F.M., Duma D., Assreuy J., Buzzi F.C., Niero R., Campos M.M., Calixto J. (2004). Caffeic acid derivatives: In Vitro and In Vivo anti-inflammatory properties. Free Rad. Res..

[299] Shi H., Xie D., Yang R., Cheng Y. (2014). Synthesis of caffeic acid phenethyl ester derivatives, and their cytoprotective and neuritogenic activities in PC12 cells. J. Agric. Food Chem..

[300] Chiang E.-P.I., Tsai S.-Y., Kuo Y.-H., Pai M.-H., Chiu H.-L., Rodriguez R.L., Tang F.-Y. (2014). Caffeic acid derivatives inhibit the growth of colon cancer: Involvement of the PI3-k/akt and ampk signaling pathways. PLoS ONE.

[301] Zhang J., Xu L.-X., Xu X.-S., Li B.-W., Wang R., Fu J.-J. (2014). Synthesis and effects of new caffeic acid derivatives on nitric oxide production in lipopolysaccharide-induced RAW 264.7 macrophages. Int. J. Clin. Exp. Med..

[302] De Lucia D., Lucio O.M., Musio B., Bender A., Listing M., Dennhardt S., Koeberle A., Garscha U., Rizzo R., Manfredini S. (2015). Design, synthesis and evaluation of semi-synthetic triazole-containing caffeic acid analogues as 5-lipoxygenase inhibitors. Eur. J. Med. Chem..

[303] Rajan P., Vedernikova I., Cos P., Vanden Berghe D., Augustyns K., Haemers A. (2001). Synthesis and evaluation of caffeic acid amides as antioxidants. Bioorg. Med. Chem. Lett..

[304] Xie Y., Huang B., Yu K., Shi F., Liu T., Xu W. (2013). Caffeic acid derivatives: A new type of influenza neuraminidase inhibitors. Bioorg. Med. Chem. Lett..

[305] Chao X., He X., Yang Y., Zhou X., Jin M., Liu S., Cheng Z., Liu P., Wang Y., Yu J. (2012). Design, synthesis and pharmacological evaluation of novel tacrine-caffeic acid hybrids as multi-targeted compounds against Alzheimer’s disease. Bioorg. Med. Chem. Lett..

[306] Hezam Al-Ostoot F., Zabiulla S., Grisha S., Hussein Eissa Mohammed Y., Vivek H.K., Ara Khanum S. (2021). Molecular docking and synthesis of caffeic acid analogous and its anti-inflammatory, analgesic and ulcerogenic studies. Bioorg. Med. Chem. Lett..

[307] Zhou D., Weng X. (2019). A novel butylated caffeic acid derivative protects hacat keratinocytes from squalene peroxidation-induced stress. Skin Pharmacol. Physiol..

[308] Chigorimbo-Murefu N.T.L., Riva S., Burton S.G. (2009). Lipase-catalysed synthesis of esters of ferulic acid with natural compounds and evaluation of their antioxidant properties. J. Mol. Catal. B Enzym..

[309] Lee G.-S., Widjaja A., Ju Y.-H. (2006). Enzymatic synthesis of cinnamic acid derivatives. Biotechnol. Lett..

[310] Sandoval G., Quintana P.G., Baldessari A., Ballesteros A.O., Plou F.J. (2015). Lipase-catalyzed preparation of mono- and diesters of ferulic acid. Biocat. Biotransf..

[311] Adeyemi O.S., Atolani O., Banerjee P., Arolasafe G., Preissner R., Etukudoh P., Ibraheem O. (2018). Computational and experimental validation of antioxidant properties of synthesized bioactive ferulic acid derivatives. Int. J. Food Prop..

[312] Ravi Kiran T.N., Sri Alekhya C., Lokesh B.V.S., Madhu Latha A.V.S., Rajendra Prasad Y., Naga Mounika T. (2015). Synthesis, characterization and biological screening of ferulic acid derivatives. J. Cancer Ther..

[313] Nomura E., Kashiwada A., Hosoda A., Nakamura K., Morishita H., Tsuno T., Taniguchi H. (2003). Synthesis of amide compounds of ferulic acid, and their stimulatory effects on insulin secretion in vitro. Bioorg. Med. Chem..

[314] Lee Y.-T., Hsieh Y.-L., Yeh Y.-H., Huang C.-Y. (2015). Synthesis of phenolic amides and evaluation of their antioxidant and anti-inflammatory activity in vitro and in vivo. RSC Adv..

[315] Huang G.-Y., Cui C., Wang Z.-P., Li Y.-Q., Xiong L.-X., Wang L.-Z., Yu S.-J., Li Z.-M., Zhao W.-G. (2013). Synthesis and characteristics of (Hydrogenated) ferulic acid derivatives as potential antiviral agents with insecticidal activity. Chem. Cent. J..

[316] Cui C., Wang Z.-P., Du X., Wang L.-Z., Yu S.-J., Liu X.-H., Li Z.-M., Zhao W.-G. (2013). Synthesis and antiviral activity of hydrogenated ferulic acid derivatives. J. Chem..

[317] Firdaus P., Soekamto N.H., Seniwati P., Islam M.F., Sultan P. (2018). Phenethyl ester and amide of ferulic acids: Synthesis and bioactivity against P388 leukemia murine cells. IOP Conf. Ser. J. Phys. Conf. Ser..

[318] Firdaus H.D.R., Naid T., Seniwati K., Soekamto N.H., Sumarna S., Islam M.F. (2017). Synthesis of piperidine and morpholine amides of ferulic acid and their bioactivity against P-388 leukemia cells. Int. J. Chemtech. Res..

[319] Kumar N., Kumar S., Abbat S., Nikhil K., Sondhi S.M., Bharatam P.V., Roy P., Pruthi V. (2015). Ferulic acid amide derivatives as anticancer and antioxidant agents: Synthesis, thermal, biological and computational studies. Med. Chem. Res..

[320] Serafim T.L., Carvalho F.S., Marques M.P.M., Calheiros R., Silva T., Garrido J., Milhazes N., Borges F., Roleira F., Silva E.T. (2011). Lipophilic caffeic and ferulic acid derivatives presenting cytotoxicity against human breast cancer cells. Chem. Res. Toxicol..

[321] Wang F., Lu W., Zhang T., Dong J., Gao H., Li P., Wang S., Zhang J. (2013). Development of novel ferulic acid derivatives as potent histone deacetylase inhibitors. Bioorg. Med. Chem..

[322] Han B.S., Park C.B., Takasuka N., Naito A., Sekine K., Nomura E., Taniguchi H., Tsuno T., Tsuda H. (2001). A ferulic acid derivative, ethyl 3-(4′-geranyloxy-3-methoxyphenyl)-2-propenoate, as a new candidate chemopreventive agent for colon carcinogenesis in the rat. Jpn. J. Cancer Res..

[323] Gaspar A., Martins M., Silva P., Garrido E.M., Garrido J., Firuzi O., Miri R., Saso L., Borge F. (2010). Dietary phenolic acids and derivatives. Evaluation of the antioxidant activity of sinapic acid and its alkyl esters. J. Agric. Food Chem..

[324] Flausino O.A., Dufau L., Regasini L.O., Petrônio M.S., Silva D.H.S., Rose T., Bolzani V.S., Reboud-Ravaux M. (2012). Alkyl hydroxybenzoic acid derivatives that inhibit HIV-1 protease dimerization. Curr. Med. Chem..

[325] Daréa R.G., Oliveira M.M., Truiti M.C.T., Nakamura C.V., Ximenes V.F., Lautenschlager S.O.S. (2020). Abilities of protocatechuic acid and its alkyl esters, ethyl and heptyl protocatechuates, to counteract UVB-induced oxidative injuries and photoaging in fibroblasts L929 cell line. J. Photochem. Photobiol. B Biol..

[326] Savi L.A., Leal P.C., Vieira T.O., Rosso R., Nunes R.J., Yunes R.A., Creczynski-Pasa T.B., Barardic C.R.M., Simões C.M.O. (2005). Evaluation of anti-herpetic and antioxidant activities, and cytotoxic and genotoxic effects of synthetic Alkyl-esters of gallic acid. Arzneim. Forsch. Drug Res..

[327] Locatelli C., Filippin-Monteiro F.B., Creczynski-Pasa T.B. (2013). Alkyl esters of gallic acid as anticancer agents: A review. Eur. J. Med. Chem..

[328] Arunkumar S., Ilango K., Mandikandan R.S., Ramalakshmi N. (2009). Synthesis and anti-inflammatory activity of some novel pyrazole derivatives of gallic acid. J. Chem..

[329] Belkheiri N., Bouguerne B., Bedos-Belval F., Duran H., Bernis C., Salvayre R., Nègre-Salvayre A., Baltas M. (2010). Synthesis and antioxidant activity evaluation of a syringic hydrazones family. Eur. J. Med. Chem..

[330] Koufaki M. (2016). Vitamin E derivatives: A patent review (2010–2015). Expert Opin. Ther. Pat..

[331] Kozubek A., Tyman J.H.P. (1999). Resorcinolic lipids, the natural non-isoprenoid phenolic amphiphiles and their biological activity. Chem. Rev..

[332] Stasiuk M., Kozubek A. (2010). Biological activity of phenolic lipids. Cell. Mol. Life Sci..

[333] Meshginfar N., Tavakoli H., Dornan K., Hosseinian F., Hosseinian F. (2021). Phenolic lipids as unique bioactive compounds: A comprehensive review on their multifunctional activity toward the prevention of Alzheimer’s disease. Crit. Rev. Food Sci. Nutr..

[334] Teixeira dos Santos A., Coelho Bernardo Guerra G., Marques J.I., Torres-Rêgo M., Ferreira Alves J.S., Carvalho Vasconcelos R., Fernandes de Souza Araújo D., Silva Abreu L., Gomes de Carvalho T., Cavalcante de Araújo D.R. (2020). Potentialities of cashew nut (*Anacardium occidentale*) by-product for pharmaceutical applications: Extraction and purification technologies, safety, and anti-inflammatory and anti-arthritis activities. Rev. Bras. Farm..

[335] Hamdy Roby M.H., Soto-Hernandex M., Palma-Tenango M., Garcia-Mateos M.d.R. (2017). Synthesis and characterization of phenolic lipids. Phenolic Compounds—Natural Sources, Importance and Applications.

[336] Reddy Jala R.C., Hu P., Yang T., Jiang Y., Zheng Y., Xu X. (2012). Lipases as biocatalysts for the synthesis of structured lipids. Methods Mol. Biol..

[337] Torres de Pinedo A., Penalver P., Rondon D., Morales J.C. (2005). Efficient lipase-catalyzed synthesis of new lipid antioxidants based on a catechol structure. Tetrahedron.

[338] Sabally K., Karboune S., St-Louis R., Kermasha S. (2006). Lipase-catalyzed transesterification of dihydrocaffeic acid with flaxseed oil for the synthesis of phenolic lipids. J. Biotechnol..

[339] Karboune S., St-Louis R., Kermasha S. (2008). Enzymatic synthesis of structured phenolic lipids by acidolysis of flaxseed oil with selected phenolic acids. J. Mol. Catal. B Enzym..

[340] Safari M., Karboune S., St-Louis R., Kermasha S. (2006). Enzymatic synthesis of structured phenolic lipids by incorporation of selected phenolic acids into triolein. Biocatal. Biotransf..

[341] Sabally K., Karboune S., St-Louis R., Kermasha S. (2007). Lipase-catalyzed synthesis of phenolic lipids from fish liver oil and dihydrocaffeic acid. Biocat. Biotransf..

[342] Aziz S., St-Louis R., Yaylayan V., Kermasha S. (2012). Chromatographic separation of synthesized phenolic lipids from krill oil and dihydroxyphenyl acetic acid. J. Am. Oil. Chem. Soc..

[343] Sorour N., Karboune S., Saint-Louis R., Kermasha S. (2012). Lipase-catalyzed synthesis of structured phenolic lipids in solvent-free system using flaxseed oil and selected phenolic acids as substrates. J. Biotechnol..

[344] Sorour N., Karboune S., Saint-Louis R., Kermasha S. (2012). Enzymatic synthesis of phenolic lipids in solvent-free medium using flaxseed oil and 3,4-dihydroxyphenyl acetic acid. Proc. Biochem..

[345] Ciftci D., Saldaña M.D.A. (2012). Enzymatic synthesis of phenolic lipids using flaxseed oil and ferulic acid in supercritical carbon dioxide media. J. Supercrit. Fluids.

[346] Reddy K.K., Shanker K.S., Ravinder T., Prasad R.B.N., Kanjilal S. (2010). Chemo-enzymatic synthesis and evaluation of novel structured phenolic lipids as potential lipophilic antioxidants. Eur. J. Lip. Sci. Techn..

[347] Balakrishna M., Shanker Kaki S., Karuna M.S.L., Sarada S.C., Kumar C.G., Prasad R.B.N. (2016). Synthesis and in vitro antioxidant and antimicrobial studies of novel structured phosphatidylcholines with phenolic acids. Food Chem..

[348] Pande G., Akoh C.C. (2016). Enzymatic synthesis of tyrosol-based phenolipids: Characterization and effect of Alkyl chain unsaturation on the antioxidant activities in bulk oil and oil-in-water emulsion. J. Am. Oil Chem. Soc..

[349] Kaki S.S., Grey C., Adlercreutz P. (2012). Bioorganic synthesis, characterization and antioxidant activity of esters of natural phenolics and a-lipoic acid. J. Biotechnol..

[350] Micillo R., Sirés-Campos J., García-Borrón J.C., Panzella L., Napolitano A., Olivares C. (2018). Conjugation with dihydrolipoic acid imparts caffeic acid ester potent inhibitory effect on dopa oxidase activity of human tyrosinase. Int. J. Mol. Sci..

[351] Johny J., Kontham V., Kaki Shiva S., Veeragoni D., Misra S. (2019). Bioorganic synthesis, characterization and evaluation of a natural phenolic lipid. Biotechnol. Rep..

[352] Sari M., Chung Y., Agatha F., Kim H.K. (2019). Evaluation of antioxidant and antimicrobial activity of phenolic lipids produced by the transesterification of 4-hydroxyphenylacetic acid and triglycerides. Appl. Biol. Chem..

[353] Dynczuki Navarro S., Adilson B., Meza A., Pesarini J.R., da Silva Gomes R., Bilhar Karaziack C., Cunha-Laura A.L., Duenhas Monreal A.C., Wanderson R., Lacerda V. (2014). A new synthetic resorcinolic lipid 3-Heptyl-3,4,6-trimethoxy-3*H*-isobenzofuran-1-one: Evaluation of toxicology and ability to potentiate the mutagenic and apoptotic effects of cyclophosphamide. Eur. J. Med. Chem..

[354] Kaki S.S., Kunduru K.R., Kanjilal S., Prasad R.B.N. (2015). Synthesis and characterization of a novel phenolic lipid for use as potential lipophilic antioxidant and as a prodrug of butyric acid. J. Oleo Sci..

[355] Buonanno F., Catalani E., Cervia D., Proietti Serafini F., Picchietti S., Fausto A.M., Giorgi S., Lupidi G., Rossi F.V., Marcantoni E. (2019). Bioactivity and structural properties of novel synthetic analogues of the protozoan toxin climacostol. Toxins.

[356] Yarra M., Kaki S.S., Prasad R.B.N., Mallampalli K.S.L., Yedla P., Ganesh K.C. (2016). Synthesis of novel (*Z*)-methyl-12-aminooctadec-9-enoate-based phenolipids as potential antioxidants and chemotherapeutic agents. Eur. J. Lip. Sci. Technol..

[357] Xie Y., Yang Y., Li S., Xu Y., Lu W., Chen Z., Yang G., Li Y., Cao Y., Bian X. (2017). Phenylsulfonylfuroxan NO-donor phenols: Synthesis and multifunctional activities evaluation. Bioorg. Med. Chem..

[358] Roleira F.M.F., Siquet C., Orrù E., Garrido E.M., Garrido J., Milhazes N., Podda G., Paiva-Martins F., Reis S., Carvalho R.A. (2010). Borges, Lipophilic phenolic antioxidants: Correlation between antioxidant profile, partition coefficients and redox properties. Bioorg. Med. Chem..

[359] Anankanbil S., Perez B., Widzisz K.M., Guo Z., Fernandes I., Mateus N., Wang Z. (2018). A new group of synthetic phenolic-containing amphiphilic molecules for multipurpose applications: Physico-chemical characterization and cell-toxicity study. Sci. Rep..

[360] Logrado L.P.L., Santos C.O., Romeiro L.A.S., Costa A.M., Ferreira J.R.O., Cavalcanti B.C., de Moraes M.O., Costa-Lotufo L.V., Pessoa C., dos Santos M.L. (2010). Synthesis and cytotoxicity screening of substituted isobenzofuranones designed from anacardic acids. Eur. J. Med. Chem..

[361] Torres de Pinedo A., Penalver P., Perez-Victoria I., Rondon D., Morales J.C. (2007). Synthesis of new phenolic fatty acid esters and their evaluation as lipophilic antioxidants in an oil matrix. Food Chem..

[362] Jin W., Zjawiony J.K. (2006). 5-Alkylresorcinols from *Merulius incarnates*. J. Nat. Prod..

[363] Parikka K., Wahala K. (2009). An expedient synthesis of 5-n-alkylresorcinols and novel 5-n-alkylresorcinol haptens. Beilst. J. Org. Chem..

[364] Menezes J.C.J.M.D.S., Kamat S.P., Cavaleiro J.A.S., Gaspar A., Garrido J., Borges F. (2011). Synthesis and antioxidant activity of long chain alkyl hydroxycinnamates. Eur. J. Med. Chem..

[365] Kashyap D., Kumar Garg V., Singh Tuli H., Yerer M.B., Sak K., Kumar Sharma A., Kumar M., Aggarwal V., Singh Sandhu S. (2019). Fisetin and quercetin: Promising flavonoids with chemopreventive potential. Biomolecules.

[366] Anand David A.V., Arulmoli R., Parasuraman S. (2016). Overviews of biological importance of quercetin: A bioactive flavonoid. Pharmacogn. Rev..

[367] Abbas M., Saeed F., Anjum F.M., Afzaal M., Tufail T., Bashir M.S., Ishtiaq A., Hussain S., Suleria H.A.R. (2017). Natural polyphenols: An overview. Int. J. Food Prop..

[368] Hirpara K.V., Aggarwal P., Mukherjee A.J., Joshi N., Burman A.C. (2009). Quercetin and its derivatives: Synthesis, pharmacological uses with special emphasis on anti-tumor properties and prodrug with enhanced bio-availability. Anti-Canc. Ag. Med. Chem..

[369] Saladino R., Gualandi G., Farina A., Crestini C., Nencioni L., Palamara A.T. (2008). Advances and challenges in the synthesis of highly oxidised natural phenols with antiviral, antioxidant and cytotoxic activities. Curr. Med. Chem..

[370] Dudnik A., Gaspar P., Neves A.R., Forster J. (2018). Engineering of microbial cell factories for the production of plant polyphenols with health-beneficial properties. Curr. Pharm. Des..

[371] Veluri R., Weir T.L., Pal Bais H., Stermitz F.R., Vivanco J.M. (2004). Phytotoxic and antimicrobial activities of catechin derivatives. J. Agric. Food Chem..

[372] Park K.D., Cho S.J. (2010). Synthesis and antimicrobial activities of 3-*O*-alkyl analogues of (+)-catechin: Improvement of stability and proposed action mechanism. Eur. J. Med. Chem..

[373] Kumar D., Poornima M., Kushwaha R.N., Won T.-J., Ahn C., Ganesh Kumar C., Jang K., Shin D.-S. (2015). Antimicrobial and docking studies of (-)-catechin derivatives. J. Kor. Soc. Appl. Biol. Chem..

[374] Fukuhara K., Ohno A., Nakanishi I., Imai K., Nakamura A., Anzai K., Miyata N., Okuda H. (2009). Novel ninhydrin adduct of catechin with potent antioxidative activity. Tetrahedron Lett..

[375] Yen C.-T., Nakagawa-Goto K., Hwang T.-L., Wu P.-C., Morris-Natschke S.L., Lai W.-C., Bastow K.F., Chang F.-R., Wub Y.-C., Lee K.-H. (2010). Antitumor agents. 271: Total synthesis and evaluation of brazilein and analogs as anti-inflammatory and cytotoxic agents. Bioorg. Med. Chem. Lett..

[376] Pan C., Zeng X., Guan Y., Jiang X., Li L., Zhang H. (2011). Design and synthesis of brazilin-like compounds. Synlett.

[377] Kostova I. (2005). Synthetic and natural coumarins as cytotoxic agents. Curr. Med. Chem. Anti Cancer Agents.

[378] Stefanachi A., Leonetti F., Pisani L., Catto M., Carotti A. (2018). Coumarin: A natural, privileged and versatile scaffold for bioactive compounds. Molecules.

[379] Khoobi M., Foroumadi A., Emami S., Safavi M., Dehghan G., Alizadeh B.H., Ramazani A., Ardestani S.K., Shafiee A. (2011). Coumarin-based bioactive compounds: Facile synthesis and biological evaluation of coumarin-fused 1,4-thiazepines. Chem. Biol. Drug Des..

[380] Symeonidis T., Chamilos M., Hadjipavlou-Litina D.J., Kallitsakis M., Litinas K.E. (2009). Synthesis of hydroxycoumarins and hydroxybenzo[*f*]- or [*h*]coumarins as lipid peroxidation inhibitors. Bioorg. Med. Chem. Lett..

[381] Perez-Vizcaino F., Duarte J., Jimenez R., Santos-Buelga C., Osuna A. (2009). Antihypertensive effects of the flavonoid quercetin. Pharm. Rep..

[382] Massi A., Bortolini O., Ragno D., Bernardi T., Sacchetti G., Tacchini M., De Risi C. (2017). Research progress in the modification of quercetin leading to anticancer agents. Molecules.

[383] Halevasa E., Pekou A., Papi R., Mavroidi B., Hatzidimitriou A.G., Zahariou G., Litsardakis G., Sagnou M., Pelecanou M., Pantazaki A.A. (2020). Synthesis, physicochemical characterization and biological properties of two novel Cu(II) complexes based on natural products curcumin and quercetin. J. Inorg. Biochem..

[384] Sharma A., Kashyap D., Sak K., Singh Tuli H., Sharma A.K. (2018). Therapeutic charm of quercetin and its derivatives: A review of research and patents. Pharm. Pat. Anal..

[385] Teles Y.C.F., Souza M.S.R., de Souza M.F.V. (2018). Sulphated flavonoids: Biosynthesis, structures, and biological activities. Molecules.

[386] Koirala N., Pandey R.P., Parajuli P., Jung H.J., Sohng J.K. (2014). Methylation and subsequent glycosylation of 7,8-dihydroxyflavone. J. Biotechnol..

[387] Antonopoulou I., Varriale S., Topakas E., Rova U., Christakopoulos P., Faraco V. (2016). Enzymatic synthesis of bioactive compounds with high potential for cosmeceutical application. Appl. Microbiol. Biotechnol..

[388] Mecenas A.S., Malafaia C.R.A., Sangenito L.S., Simas D.L.R., de Barros Machado T., Amaral A.C.F., Santos A.L.S.D., Freire D.M.G., Leal I.C.R. (2018). Rutin derivatives obtained by transesterification reactions catalyzed by Novozym 435: Antioxidant properties and absence of toxicity in mammalian cells. PLoS ONE.

[389] Li X., Mai W., Chen D. (2014). Chemical study on protective effect against hydroxyl-induced dna damage and antioxidant mechanism of myricitrin. J. Chin. Chem. Soc..

[390] Deng X., Wang Z., Liu J., Xiong S., Xiong R., Cao X., Chen Y., Zheng X., Tang G. (2017). Design, synthesis and biological evaluation of flavonoid salicylate derivatives as potential anti-tumor agents. RSC Adv..

[391] Poerwono H., Sasaki S., Hattori Y., Higashiyama K. (2010). Efficient microwave-assisted prenylation of pinostrobin and biological evaluation of its derivatives as antitumor agents. Bioorg. Med. Chem. Lett..

[392] Marliyana S.D., Mujahidin D., Syah Y.M. (2018). pinostrobin derivatives from prenylation reaction and their antibacterial activity against clinical bacteria. IOP Conf. Ser. Mater. Sci. Eng..

[393] Tian J.-L., Liu T.-L., Xue J.-J., Hong W., Zhang Y., Zhang D.-X., Cui C.-C., Liu M.-C., Niu S.-L. (2019). Flavanoids derivatives from the root bark of *Broussonetia papyrifera* as a tyrosinase inhibitor. Ind. Crop. Prod..

[394] Thuan N.H., Malla S., Trung N.T., Dhakal D., Pokhrel A.R., Chu L.L., Sohng J.K. (2017). Microbial production of astilbin, a bioactive rhamnosylated flavanonol, from taxifolin. World J. Microbiol. Biotechnol..

[395] Thapa S.B., Pandey R.P., Bashyal P., Tokutaro Y., Sohng J.Y. (2019). Cascade biocatalysis systems for bioactive naringenin glucosides and quercetin rhamnoside production from sucrose. Appl. Microbiol. Biotechnol..

[396] Ben Kaab S., Lins L., Hanafi M., Bettaieb Rebey I., Deleu M., Fauconnier M.-L., Ksouri R., Jijakli M.H., De Clerck C. (2020). *Cynara cardunculus* crude extract as a powerful natural herbicide and insight into the mode of action of its bioactive molecules. Biomolecules.

[397] Preetha A., Sherin G.T., Ajaikumar B.K., Chitra S., Kuzhuvelil B.H., Bokyung S., Sheeja T.T., Krishna M., Priyadarsini I.K., Rajasekharan K.N. (2008). Biological activities of curcumin and its analogues (Congeners) made by man and Mother Nature. Biochem. Pharm..

[398] Bukhari S.N.A., Bin Jantan I., Jasamai M., Ahmad W., Bin Amjad W. (2013). Synthesis and biological evaluation of curcumin analogues. J. Med. Sci..

[399] Amalraj A., Pius A., Gopi S., Gopi S. (2017). Biological activities of curcuminoids, other biomolecules from turmeric and their derivatives—A review. J. Trad. Compl. Med..

[400] Oglah M.K., Mustafa Y.F., Bashir M.K., Jasim M.H. (2020). Curcumin and its derivatives: A review of their biological activities. Sys. Rev. Pharm..

[401] Rodrigues F.C., Anil Kumar N.V., Thakur G. (2019). Developments in the anticancer activity of structurally modified curcumin: An up-to-date review. Eur. J. Med. Chem..

[402] Noureddin S.S., El-Shishtawy R.M., Al-Footy K.O. (2019). Curcumin analogues and their hybrid molecules as multifunctional drugs. Eur. J. Med. Chem..

[403] Mbese Z., Khwaza V., Aderibigbe B.A. (2019). Curcumin and its derivatives as potential therapeutic agents in prostate, colon and breast cancers. Molecules.

[404] Zangui M., Atkin S.L., Majeed M., Sahebkar A. (2019). Current evidence and future perspectives for curcumin and its analogues as promising adjuncts to oxaliplatin: State-of-the-art. Pharmacol. Res..

[405] Selvam C., Prabu S.L., Jordan B.C., Purushothaman Y., Umamaheswari A., Hosseini Zare M.S., Thilagavathi R. (2019). Molecular mechanisms of curcumin and its analogs in colon cancer prevention and treatment. Life Sci..

[406] Lo Cascio F., Marzullo P., Kayed R., Palumbo Piccionello A. (2021). Curcumin as scaffold for drug discovery against neurodegenerative diseases. Biomedicines.

[407] Chainoglou E., Hadjipavlou-Litina D. (2020). Curcumin in health and diseases: Alzheimer’s disease and curcumin analogues, derivatives, and hybrids. Int. J. Mol. Sci..

[408] Mansouri K., Rasoulpoor S., Daneshkhah A., Abolfathi S., Salari N., Mohammadi M., Rasoulpoor S., Shabani S. (2020). Clinical effects of curcumin in enhancing cancer therapy: A systematic review. BMC Cancer.

[409] Abdellah A.M., Sliem M.A., Bakr M., Amin R.M. (2018). Green synthesis and biological activity of silver–curcumin nanoconjugates. Future Med. Chem..

[410] Koo H.J., Shin S., Choi J.Y., Lee K.H., Kim B.T., Choe Y.S. (2015). Introduction of methyl groups at C2 and C6 positions enhances the antiangiogenesis activity of curcumin. Sci. Rep..

[411] Edwards R.L., Luis P.B., Varuzza P.V., Joseph A.I., Presley S.H., Chaturvedi R., Schneider C. (2017). The anti-inflammatory activity of curcumin is mediated by its oxidative metabolites. J. Biol. Chem..

[412] Joseph A.I., Edwards R.L., Luis P.B., Presley S.H., Porter N.A., Schneider C. (2018). Stability and anti-inflammatory activity of the reduction-resistant curcumin analog, 2,6-dimethyl-curcumin. Org. Biomol. Chem..

[413] De Cássia Orlandi Sardi J., Polaquini C.R., Almeida Freires I., Câmara de Carvalho Galvão L., Goldoni Lazarini J., Silva Torrezan G., Regasini L.O., Rosalen P.L. (2017). Antibacterial activity of diacetylcurcumin against *Staphylococcus aureus* results in decreased biofilm and cellular adhesion. J. Med. Microbiol..

[414] Escobedo-Martínez C., Guzmán-Gutiérrez S.L., Carrillo-López M.I., Deveze-Álvarez M.A., Trujillo-Valdivia A., Meza-Morales W., Enríquez R.G. (2019). Diacetylcurcumin: Its potential antiarthritic effect on a freund’s complete adjuvant-induced murine model. Molecules.

[415] Somparn P., Phisalaphong C., Nakornchai S., Unchern S., Morales N.P. (2007). Comparative antioxidant activities of curcumin and its demethoxy and hydrogenated derivatives. Biol. Pharm. Bull..

[416] Rosa A., Atzeri A., Deiana M., Melis M.P., Incani A., Minassi A., Cabboi B., Appendino G. (2014). Prenylation preserves antioxidant properties and effect on cell viability of the natural dietary phenol curcumin. Food Res. Int..

[417] Abonia R., Laali K.K., Raja Somu D., Bunge S.D., Wang E.C. (2020). A flexible strategy for modular synthesis of curcuminoid-BF2/curcuminoid pairs and their comparative anti-proliferative activity in human cancer cell lines. Chem. Med. Chem..

[418] Buyandelger U., Walker D.G., Taguchi H., Yanagisawa D., Tooyama I. (2020). Novel fluorinated derivative of curcumin negatively regulates thioredoxin-interacting protein expression in retinal pigment epithelial and macrophage cells. Biochem. Biophys. Res. Commun..

[419] Mishra S., Karmodiya K., Surolia N., Surolia A. (2008). Synthesis and exploration of novel curcumin analogues as anti-malarial agents. Bioorg. Med. Chem..

[420] Radha A., Rukhmini S.D., Vilasini S., Sakunthala P.R., Sreedharan B., Velayudhan M.P., Abraham A. (2012). Bioactive derivatives of curcumin attenuate cataract formation in vitro. Chem. Biol. Drug. Des..

[421] Wolosewicz K., Podgorska K., Rutkowska E., Lazny R. (2019). Synthesis of dicarbonyl curcumin analogues containing the tropane scaffold. Eur. J. Org. Chem..

[422] Liu Y., Dargusch R., Maher P., Schubert D. (2008). A broadly neuroprotective derivative of curcumin. J. Neurochem..

[423] Mahera P., Akaishi T., Schubert D., Abe K. (2010). A pyrazole derivative of curcumin enhances memory. Neurobiol. Aging.

[424] Akaishi T., Yamamoto S., Abe K. (2020). The synthetic curcumin derivative CNB-001 attenuates thrombin-stimulated microglial inflammation by inhibiting the ERK and p38 MAPK pathways. Biol. Pharm. Bull..

[425] Khaldi-Khellafi N., Makhloufi-Chebli M., Oukacha-Hikem D., Bouaziz S.T., Lamara K.O., Idir T., Benazzouz-Touami A., Dumas F. (2019). Green synthesis, antioxidant and antibacterial activities of 4-aryl-3,4-dihydropyrimidinones/thiones derivatives of curcumin. Theoretical calculations and mechanism study. J. Mol. Struct..

[426] Huang J., Fu J., Liu B., Wang R., You T. (2020). A Synthetic curcuminoid analog, (*2*E,6*E*)-2,6-bis(2-(trifluoromethyl)benzylidene)cyclohexanone, ameliorates impaired wound healing in streptozotocin-induced diabetic mice by increasing miR-146a. Molecules.

[427] Pignanelli C., Ma D., Noel M., Ropat J., Mansour F., Curran C., Pupulin S., Larocque K., Wu J., Liang G. (2017). Selective targeting of cancer cells by oxidative vulnerabilities with novel curcumin analogs. Sci. Rep..

[428] Parashar K., Sood S., Mehaidli A., Curran C., Vegh C., Nguyen C., Pignanelli C., Wu J., Liang G., Wang Y. (2019). Evaluating the anti-cancer efficacy of a synthetic curcumin analog on human melanoma cells and its interaction with standard chemotherapeutics. Molecules.

[429] Hisamuddin N., Shaik Mossadeq W.M., Sulaiman M.R., Abas F., Leong S.W., Kamarudin N., Ong H.M., Ahmad Azmi A.F., Ayumi R.R., Talib M. (2019). Anti-edematogenic and anti-granuloma activity of a synthetic curcuminoid analog, 5-(3,4-dihydroxyphenyl)-3-hydroxy-1-(2-hydroxyphenyl)penta-2,4-dien-1-one, in mouse models of inflammation. Molecules.

[430] Lee E.S., Kwon M.-H., Kim H.M., Woo H.B., Ahn C.M., Chung C.H. (2020). Curcumin analog CUR5–8 ameliorates nonalcoholic fatty liver disease in mice with high-fat diet-induced obesity. Metab. Clin. Exp..

[431] Kazantzis K.T., Koutsonikoli K., Mavroidi B., Zachariadis M., Alexiou P., Pelecanou M., Politopoulos K., Alexandratou E., Sagnou M. (2020). Curcumin derivatives as photosensitizers in photodynamic therapy: Photophysical properties and in vitro studies with prostate cancer cells. Photochem. Photobiol. Sci..

[432] Yanagisawa D., Shirai N., Amatsubo T., Taguchi H., Hirao K., Urushitani M., Morikawa S., Inubushi T., Kato M., Kato F. (2010). Relationship between the tautomeric structures of curcumin derivatives and their Aβ-binding activities in the context of therapies for Alzheimer’s disease. Biomaterials.

[433] Taguchi H., Yanagisawa D., Morikawa S., Hirao K., Shirai N., Tooyama I. (2015). Synthesis and tautomerism of curcumin derivatives and related compounds. Aust. J. Chem..

[434] Yanagisawa D., Taguchi H., Morikawa S., Kato T., Hirao K., Shirai N., Tooyama I. (2015). Novel curcumin derivatives as potent inhibitors of amyloid β aggregation. Biochem. Biophys. Rep..

[435] Yanagisawa D., Kato T., Taguchi H., Shirai N., Hirao K., Sogabe T., Tomiyama T., Gamo K., Hirahara Y., Kitada M. (2021). Keto form of curcumin derivatives strongly binds to Aβ oligomers but not fibrils. Biomaterials.

[436] Ferrari E., Pignedoli F., Imbriano C., Marverti G., Basile V., Venturi E., Saladini M. (2011). Newly synthesized curcumin derivatives: Crosstalk between chemico-physical properties and biological activity. J. Med. Chem..

[437] Ahmed M., Qadir M.A., Shafiq M.I., Muddassar M., Samra Z.Q., Hameed A. (2019). Synthesis, characterization, biological activities and molecular modeling of Schiff bases of benzene sulfonamides bearing curcumin scaffold. Arab. J. Chem..

[438] Liang G., Li X., Chen L., Yang S., Wu X., Studer E., Gurley E., Hylemon P.B., Ye F., Li Y. (2008). Synthesis and anti-inflammatory activities of mono-carbonyl analogues of curcumin. Bioorg. Med. Chem. Lett..

[439] Liang G., Shao L., Wang Y., Zhao C., Chu Y., Xiao J., Zhao Y., Li X., Yang S. (2009). Exploration and synthesis of curcumin analogues with improved structural stability both in vitro and in vivo as cytotoxic agents. Bioorg. Med. Chem..

[440] Vanchinathan K., Bhagavannarayana G., Muthu K., Meenakshisundaram S.P. (2011). Synthesis, crystal growth and characterization of 1,5-diphenylpenta-1,4- dien-3-one: An organic crystal. Phys. B Condens. Matter..

[441] Patel H., Mothia B., Patel J., Fasanya O., Sooda K., Javid F., Wyatt P.B. (2020). Cytotoxicity of some synthetic bis(arylidene) derivatives of cyclic ketones towards cisplatin-resistant human ovarian carcinoma cells. Med. Chem. Res..

[442] Da Silva C.C., Silveira Pacheco B., das Neves R.N., Dié Alves M.S., Sena-Lopes Ǻ., Moura S., Borsuk S., de Pereira C.M.P. (2019). Antiparasitic activity of synthetic curcumin monocarbonyl analogues against *Trichomonas vaginalis*. Biomed. Pharmacother..

[443] Tavaf Z., Dangolani S.K., Yousefi R., Panahi F., Shahsavani M.B., Khalafi-Nezhad A. (2020). Synthesis of new curcumin derivatives as influential antidiabetic α-glucosidase and α-amylase inhibitors with anti-oxidant activity. Carbohydr. Res..

[444] Nakamae I., Morimoto T., Shima H., Shionyu M., Fujiki H., Yoneda-Kato N., Yokoyama T., Kanaya S., Kakiuchi K., Shirai T. (2019). Curcumin derivatives verify the essentiality of ROS upregulation in tumor suppression. Molecules.

[445] Ali N., Yeap S.K., Abu N., Lim K.L., Ky H., Pauzi A.Z.M., Ho W.Y., Tan S.W., Alan-Ong H.K., Zareen S. (2017). Synthetic curcumin derivative DK1 possessed G2/M arrest and induced apoptosis through accumulation of intracellular ROS in MCF-7 breast cancer cells. Cancer Cell Signal.

[446] Yazmin H., Muhammad Nazirul Mubin A., Nurul Fattin Che R., Swee Keong Y., Nurul Elyani M., Mas Jaffri M., Noraini N., Afizan-Nik Abd Rahman N.M., Yong C.Y., Nadeem Akhtar M. (2018). DK1 induces apoptosis via mitochondria-dependent signaling pathway in human colon carcinoma cell lines in vitro. Int. J. Mol. Sci..

[447] Aziz M.N.M., Hussin Y., Rahim N.F.C., Nordin N., Mohamad N.E., Yeap S.K., Yong C.Y., Masarudin M.J., Cheah Y.K., Abu N. (2018). Curcumin analog DK1 induces apoptosis in human osteosarcoma cells in vitro through mitochondria-dependent signaling pathway. Molecules.

[448] Obregón-Mendoza M.A., Estévez-Carmona M.M., Hernández-Ortega S., Soriano-García M., Ramírez-Apan M.T., Orea L., Pilotzi H., Gnecco D., Cassani J., Enríquez R.G. (2017). Retro-curcuminoids as mimics of dehydrozingerone and curcumin: Synthesis, nmr, X-ray, and cytotoxic activity. Molecules.

[449] De Freitas Silva M., Ferreira Coelho L., Mitestainer Guirelli I., Machado Pereira R., Ferreira da Silva G.Á., Graravelli G.Y., de Oliveira Horvath R., Caixeta Nogueira E.S., Ionta M., Viegas C. (2018). Synthetic resveratrol-curcumin hybrid derivative inhibits mitosis progression in estrogen positive mcf-7 breast cancer cells. Toxicol. Vitro.

[450] Spaeth A., Graeler A., Maisch T., Plaetzer K. (2018). CureCuma–cationic curcuminoids with improved properties and enhanced antimicrobial photodynamic activity. Eur. J. Med. Chem..

